# Genome-Wide Investigation of Maximum Habitual Alcohol Intake in US Veterans in Relation to Alcohol Consumption Traits and Alcohol Use Disorder

**DOI:** 10.1001/jamanetworkopen.2022.38880

**Published:** 2022-10-27

**Authors:** Joseph D. Deak, Daniel F. Levey, Frank R. Wendt, Hang Zhou, Marco Galimberti, Henry R. Kranzler, J. Michael Gaziano, Murray B. Stein, Renato Polimanti, Joel Gelernter

**Affiliations:** 1 Yale School of Medicine, New Haven, Connecticut; 2 VA Connecticut Healthcare Center, West Haven, Connecticut; 3 University of Pennsylvania Perelman School of Medicine, Philadelphia; 4 Crescenz VA Medical Center, Philadelphia, Pennsylvania; 5 Massachusetts Veterans Epidemiology and Research Information Center (MAVERIC), Boston Veterans Affairs Healthcare System, Boston; 6 Department of Medicine, Divisions of Aging and Preventative Medicine, Brigham and Women’s Hospital, Boston, Massachusetts; 7 Department of Medicine, Harvard Medical School, Boston, Massachusetts; 8 University of California, San Diego, La Jolla; 9 VA San Diego Healthcare System, San Diego, California

## Abstract

**Importance:**

Alcohol genome-wide association studies (GWASs) have generally focused on alcohol consumption and alcohol use disorder (AUD); few have examined habitual drinking behaviors like maximum habitual alcohol intake (MaxAlc).

**Objectives:**

To identify genetic loci associated with MaxAlc and to elucidate the genetic architecture across alcohol traits.

**Design, Setting, and Participants:**

This MaxAlc genetic association study was performed among Million Veteran Program participants enrolled from January 10, 2011, to September 30, 2020. Ancestry-specific GWASs were conducted in participants with European (n = 218 623) and African (n = 29 132) ancestry, then meta-analyzed (N = 247 755). Linkage-disequilibrium score regression was used to estimate single nucleotide variant (SNV)–heritability and genetic correlations (*r*_g_) with other alcohol and psychiatric traits. Genomic structural equation modeling (gSEM) was used to evaluate genetic associations between MaxAlc and other alcohol traits. Mendelian randomization was used to examine potential causal relationships between MaxAlc and liver enzyme levels. MTAG (multitrait analysis of GWAS) was used to analyze MaxAlc and problematic alcohol use (PAU) jointly.

**Exposures:**

Genetic associations.

**Main Outcomes and Measures:**

MaxAlc was defined from the following survey item: “in a typical month, what is/was the largest number of drinks of alcohol you may have had in one day?” with ordinal responses from 0 to 15 or more drinks.

**Results:**

GWASs were conducted on sample sizes of as many as 247 455 US veterans. Participants were 92.68% male and had mean (SD) age of 65.92 (11.70) years. The MaxAlc GWAS resulted in 15 genome-wide significant loci. Top associations in European-ancestry and African-ancestry participants were with known functional variants in the *ADH1B *gene, namely rs1229984 (*P* = 3.12 × 10^−101^) and rs2066702 (*P* = 6.30 × 10^−17^), respectively. Novel associations were also found. SNV-heritability was 6.65% (SE, 0.41) in European-ancestry participants and 3.42% (SE, 1.46) in African-ancestry participants. MaxAlc was positively correlated with PAU (*r*_g_ = 0.79; *P* = 3.95 × 10^−149^) and AUD (*r*_g_ = 0.76; *P* = 1.26 × 10^−127^) and had negative *r*_g_ with the UK Biobank “alcohol usually taken with meals” (*r*_g_ = −0.53; *P* = 1.40 × 10^−50^). For psychiatric traits, MaxAlc had the strongest genetic correlation with suicide attempt (*r*_g_ = 0.40; *P* = 3.02 × 10^−21^). gSEM supported a 2-factor model with MaxAlc loading on a factor with PAU and AUD and other alcohol consumption measures loading on a separate factor. Mendelian randomization supported an association between MaxAlc and the liver enzyme gamma-glutamyltransferase (β = 0.012; *P* = 2.66 × 10^−10^). MaxAlc MTAG resulted in 31 genome-wide significant loci.

**Conclusions and Relevance:**

The findings suggest that MaxAlc closely aligns genetically with PAU traits. This study improves understanding of the mechanisms associated with normative alcohol consumption vs problematic habitual use and AUD as well as how MaxAlc relates to psychiatric and medical conditions genetically and biologically.

## Key Points

**Question:**

What is the genetic architecture of maximum habitual alcohol intake (MaxAlc), and how does it compare with other alcohol consumption measures?

**Findings:**

This genetic association study of MaxAlc in 247 455 European- and African-ancestry individuals identified 15 genome-wide significant loci, including multiple novel associations. MaxAlc was genetically correlated with measures of alcohol-related problems, demonstrated significantly different correlations with psychiatric traits compared with other alcohol consumption traits, and loaded on a factor with alcohol problem traits, while alcohol consumption measures loaded on a separate factor.

**Meaning:**

These findings suggest that MaxAlc is genetically different from consumption measures in relation to problematic alcohol use.

## Teaser

This genetic association study identifies genetic loci associated with maximum habitual alcohol intake and assesses the genetic architecture across alcoholic traits.

## Introduction

Progress has been made in understanding the genetic architecture of many alcohol-related traits,^1,2^ including alcohol use disorder (AUD),^3,4^ forms of problematic alcohol use (PAU),^4,5^ and measures of alcohol consumption.^3,5,6^ A major contribution that genome-wide association studies (GWASs) have made to understanding the genetics of alcohol traits is the finding that the genetic architecture of AUD differs from alcohol use measures.^2^ Genetic correlation analyses have overturned the idea that alcohol use traits differ from AUD only in degree of use by showing consistently that they differ in nature too.^3,4,5^ Quantity-frequency measures like the Alcohol Use Disorders Identification Test–Consumption (AUDIT-C) score correlate genetically with positive metabolic outcomes but not psychiatric disorders, while AUD correlates genetically with psychiatric disorders.^3,4^ Although these findings have been replicated, they are not well understood and warrant further study.^7^ Few well-powered efforts have examined the genetic influences across levels of habitual alcohol consumption—an important area of investigation given that higher levels of alcohol intake often lead to increased negative alcohol outcomes.

We aim to improve understanding of the genetic architecture of habitual alcohol consumption by greatly increasing the sample size of GWAS of maximum habitual alcohol intake (MaxAlc)^8^ in European and African ancestry participants in the Million Veteran Program (MVP) and examining the similarities and differences between MaxAlc and other alcohol traits. MaxAlc can be distinguished from similar phenotypes that measure the maximum number of drinks consumed in a lifetime drinking episode, in that MaxAlc captures habitual maximum alcohol intake (ie, “in a typical month”), while lifetime maximum drinks may capture consumption on only a single occasion, often being influenced by milestone drinking events (eg, 21st birthday celebrations).^9^ Because MaxAlc is more representative of routine behaviors, it is likely more clinically meaningful and representative of behavior that could result in more negative alcohol-related outcomes than occasional binge-drinking or lower levels of alcohol consumption.^10^

To our knowledge, the current study is the largest GWAS of MaxAlc to date. The larger sample enables us to estimate the heritability of MaxAlc and examine its genetic correlations with traits from large studies of alcohol consumption and alcohol-related problems. We used genomic structural equation modeling (gSEM) to evaluate the overarching genetic relationships between MaxAlc and other alcohol traits. To investigate the association of MaxAlc with long-term alcohol-related disease, we conducted mendelian randomization (MR) to examine potential relationships between MaxAlc and liver enzyme levels. Together, these analyses provide a better understanding of the genetic basis of regular heavy drinking. We hypothesized that MaxAlc would be more genetically similar to AUD than to other alcohol consumption measures.

## Method

### Participants and Phenotyping

MVP participants are US military veterans enrolled through the Department of Veteran Affairs (VA) health care system and studied in the United States.^11^ MaxAlc was defined using information from the MVP Lifestyle Survey, a self-report survey composed of questions from validated instruments,^12^ question: “in a typical month, what is/was the largest number of drinks of alcohol you may have had in one day?” with ordinal response options ranging from 0 to 15 or more drinks (eFigure 1 in Supplement 1). We removed individuals with second-degree or closer relatives pairwise based on a kinship coefficient of greater than 0.0884, prioritizing the retention of the individual with the higher reported MaxAlc value (the heavier drinking member of the pair of related individuals). The study was approved by the Central VA institutional review board (IRB) and site-specific IRBs, including Yale University School of Medicine and VA Connecticut, and was conducted in accordance with all relevant ethical regulations. Written informed consent was obtained from all participants. This study followed the Strengthening the Reporting of Genetic Association Studies (STREGA) reporting guideline.^13^ Genotyping and imputation for MVP are described in the eAppendix in Supplement 1.

### Ancestry-Specific GWAS and Cross-Ancestry GWAS Meta-analysis

Individual GWASs were conducted on the ordinal trait in the respective MVP samples of participants with European and African ancestry using a linear regression model implemented in PLINK 2.0.^14^ Age, sex, and the first 10 within-ancestry genetic principal components were included as covariates. Ancestry-specific GWASs were then meta-analyzed across ancestries using inverse-variance weighting in METAL.^15^ 1KG Phase 3 was used as a reference panel to determine European and African ancestry linkage disequilibrium (LD) structure.^16^ Independent GWAS loci were identified using an LD threshold of *r*^2^ = 0.1 to determine lead single nucleotide variant (SNVs) at each genome-wide significant locus.^17^ Variants were assigned to the nearest gene based on physical position (<10 kilobase from the assigned gene). Additional gene-mapping techniques using expression quantitative trait locus and 3-dimensional chromatin interactions data were also performed and are described in the eAppendix in Supplement 1.

### Gene, Gene Set, and Tissue-Specific Gene Expression Analysis

We conducted gene, gene set, and gene expression analyses. These are described in the eAppendix in Supplement 1.

### Statistical Analysis

#### SNV-Heritability and Genetic Correlations

LD score regression (LDSC)^18^ was used to estimate SNV-heritability (h^2^_SNV_) and to characterize genetic correlations (*r*_g_) with a set of alcohol, psychiatric, and medical traits. Genetic correlations were examined for MaxAlc with GWASs of AUD,^4^ PAU,^4^ drinks per week (DPW),^6^ a UK Biobank (UKB) measure of “when you drink alcohol, is it usually with meals?” (UKB Field ID 1618), and the AUDIT total (AUDIT-T), problems (AUDIT-P), and consumption (AUDIT-C) scales^3,5^ in European-ancestry participants. The AUDIT is a 10-item screening-tool for AUD, with the first 3 items assessing alcohol consumption (AUDIT-C) and the final 7 items assessing alcohol-related problems (AUDIT-P).^19^ Included traits are described in greater detail in the eAppendix in Supplement 1. Measures of AUDIT-C from 2 samples that have demonstrated differences in substance use problems and demographic composition, MVP and UKB, were included to examine distinctions between the measure across these populations. COV-LDSC^20^ was used to estimate MaxAlc h^2^_SNV_ in African-ancestry participants.

Large-scale GWASs of AUD and PAU, unlike alcohol consumption measures, have shown positive genetic correlations with psychiatric disorders.^3,4^ GWASs have not yet been used to examine patterns of genetic correlation between psychopathology and heavier alcohol consumption (eg, MaxAlc) in comparison with AUD and other consumption measures. Thus, we performed genetic correlation analyses between the included alcohol traits and GWASs of anxiety,^21^ depression,^22^ posttraumatic stress disorder (PTSD),^23^ schizophrenia,^24^ and suicide attempt.^25^

#### gSEM of Alcohol Traits

gSEM^26^ was used to evaluate the shared polygenic architecture of MaxAlc, alcohol use problems, and other alcohol consumption measures using GWAS summary statistics. Exploratory factor analysis (EFA) was used to evaluate 1-, 2-, and 3-factor model fit. Confirmatory factor analysis (CFA) was then performed to evaluate model convergence and factor loadings using conventional model fit indices.^26^ CFA models were estimated using diagonally weighted least squares estimation and a smoothed genetic covariance matrix to guard against nonpositive definite scenarios. The 1KG Phase 3 European ancestry reference panel was used to calculate LD.^16^

#### MR Analyses

We used MR to examine potential relationships between MaxAlc and alcohol-related liver enzyme levels with available GWAS data (ALP [alkaline phosphatase], ALT [alanine transaminase], and GGT [gamma-glutamyl transferase])^27^ using inverse-variance weighted (IVW), weighted median, and MR-Egger methods in TwoSampleMR^28^ and MR-PRESSO.^29^ MR analysis was limited to liver enzymes demonstrating a significant genetic correlation with MaxAlc. To ensure sufficient genetic instruments to test for causal relationships, we included LD-independent MaxAlc loci reaching a suggestive significance threshold of *P* < 5.0 × 10 ^−5^ (171 SNVs). Multiple methods were used to evaluate method-specific biases to determine consistent effect estimates. Sensitivity analyses, leave-one-out analyses, and outlier removal were all evaluated. The MR-Egger intercept test was used to evaluate the presence of horizontal pleiotropy. MR-PRESSO^29^ was used to identify genetic instrument outliers that show horizontal pleiotropy for removal. MR–Robust Adjusted Profile Score (MR-RAPS)^30^ was used to test for biased effect estimates due to the inclusion of suggestive significance SNVs as genetic instruments.

#### Multitrait Analysis of MaxAlc and PAU

A joint analysis of European ancestry MaxAlc and PAU^4^ GWAS summary statistics was performed in multitrait analysis of GWAS (MTAG).^31^ The MTAG analysis is described in the eAppendix in Supplement 1.

## Results

MVP participants of European and African ancestry with MaxAlc phenotypic and genotypic data (European ancestry, 218 623; African ancestry, 29 132) were included in the GWAS analyses, resulting in a total sample of 247 755 participants. The total sample was 92.68% male and had a mean (SD) age of 65.92 (11.70) years. Overall, 36.92% reported MaxAlc at the binge-drinking threshold or greater^32^ (female participants, ≥4 drinks; male participants, ≥5 drinks) (eTable 1 in Supplement 2 and eFigure 2 in Supplement 1).

### Ancestry-Specific GWAS and Cross-Ancestry GWAS Meta-analysis

**Figure 1.**
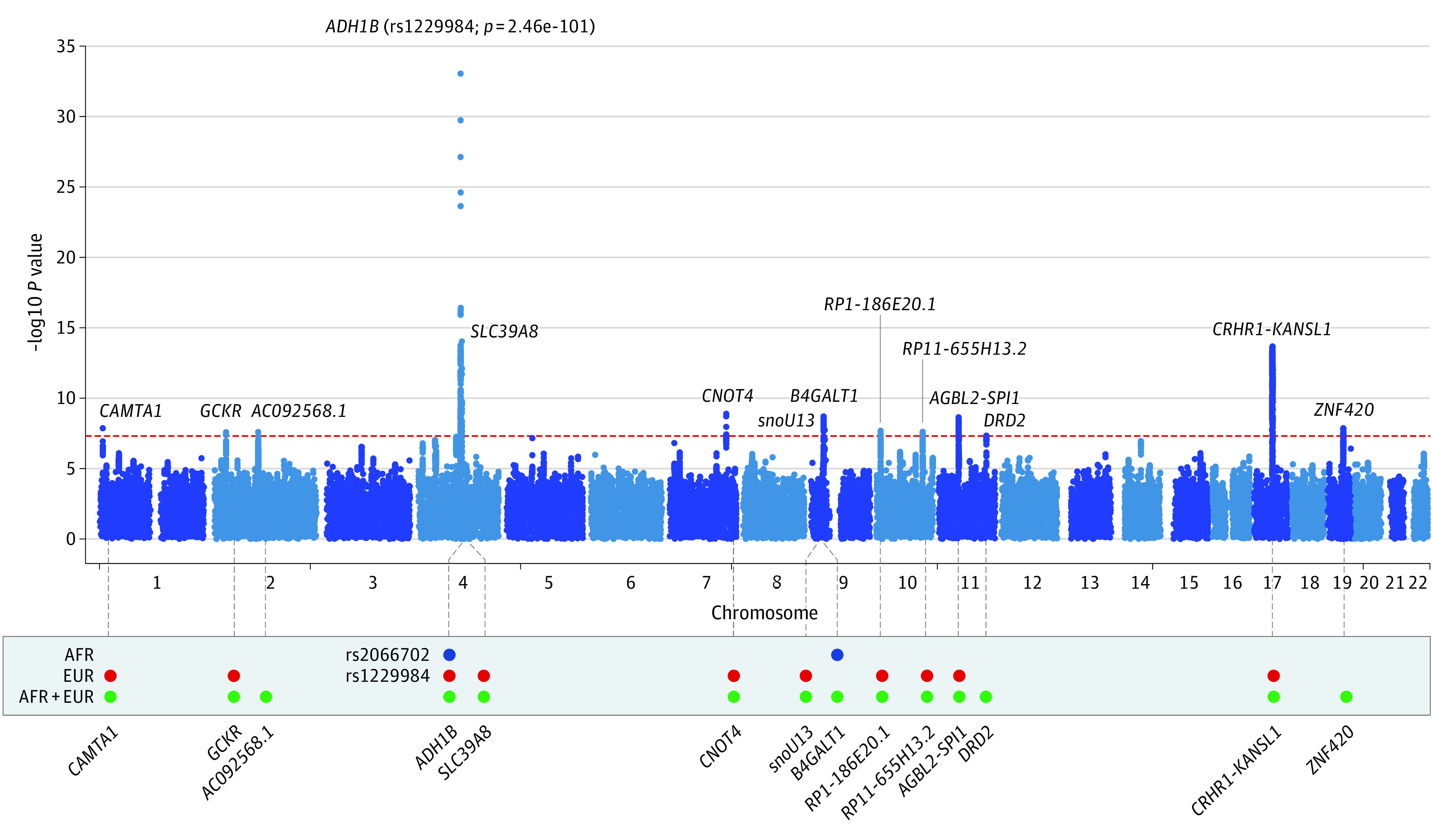
Manhattan Plot of Maximum Habitual Alcohol Intake GWAS in 247 755 Individuals of African and European Ancestry Red-dashed line indicates genome-wide significance (*P* = 5.00 × 10^−8^). In the lower panel, blue circles indicate genome-wide significance in the African-ancestry analysis (AFR); red circles indicate genome-wide significance in the European-ancestry analysis (EUR); and green circles indicate genome-wide significance in the cross-ancestry meta-analysis (AFR+EUR).

The MaxAlc GWAS resulted in 15 LD-independent genome-wide significant (*P* ≤ 5.0 × 10^−08^) loci across the respective analyses (Figure 1 and Table 1). There were 10 genome-wide significant loci in the European-ancestry analysis, 2 genome-wide significant loci in the African-ancestry analysis, and 3 additional genome-wide significant loci in the cross-ancestry meta-analysis that did not reach genome-wide significance in the ancestry-specific analyses.

**Table 1.** Genome-Wide Significant Maximum Habitual Alcohol Intake Loci in the Cross-Ancestry GWAS, European GWAS, African Ancestry GWAS, and MaxAlc MTAG GWAS

Chr	Position	Marker name	A1	A2	Nearest gene	Direction or MAF^a^	*P* value	SNVs, No.	GWAS SNVs, No.	IS SNVs, No.	Lead SNVs, No.
**Cross-ancestry GWAS**											
4	100239319	rs1229984	C	T	*ADH1B*	-?	2.46 × 10^−101^	529	393	22	9
4	103188709	rs13107325	T	C	*SLC39A8*	–	9.40 × 10^−15^	23	23	4	1
17	44305199	rs570285046	C	T	*CRHR1*	++	2.12 × 10^−14^	3590	3538	29	3
7	135162897	rs2551763	T	C	*CNOT4*	-?	1.29 × 10^−9^	29	18	1	1
9	30359634	rs13297433	A	G	*snoU13*	++	2.01 × 10^−9^	76	73	2	1
11	47392114	rs7928419	A	G	*SPI1*	++	2.29 × 10^−9^	196	172	3	1
1	7830262	rs2153733	C	T	*CAMTA1*	+?	1.36 × 10^−8^	1	1	1	1
19	37504953	rs826323	A	G	*ZNF420*	++	1.39 × 10^−8^	162	153	1	1
9	33094361	rs56175925	A	G	*B4GALT1*	?+	1.82 × 10^−8^	5	5	1	1
10	10704659	rs34165302	C	CA	*RP1-186E20.1*	-?	2.08 × 10^−8^	87	35	1	1
10	110572259	rs1577857	G	T	*RP11-655H13.2*	+?	2.47 × 10^−8^	100	82	1	1
2	104056769	rs6752675	A	G	*AC092568.1*	–	2.57 × 10^−8^	196	170	1	1
2	27730940	rs1260326	C	T	*GCKR*	–	2.63 × 10^−8^	9	9	1	1
11	113392994	rs2514218	T	C	*DRD2*	–	4.59 × 10^−8^	25	23	1	1
**European-ancestry GWAS**											
4	100239319	rs1229984	C	T	*ADH1B*	0.04	3.12 × 10^−101^	420	275	14	8
4	103188709	rs13107325	T	C	*SLC39A8*	0.08	6.78 × 10^−15^	24	24	2	1
17	43810896	rs77804065	T	C	*CRHR1*	0.23	6.04 × 10^−14^	3839	3533	4	2
11	47676170	rs7107356	G	A	*AGBL2*	0.5	1.43 × 10^−10^	422	313	3	1
7	135162897	rs2551763	T	C	*CNOT4*	0.41	1.30 × 10^−9^	43	19	1	1
1	7830262	rs2153733	C	T	*CAMTA1*	0.19	1.36 × 10^−8^	35	31	1	1
10	10714452	rs145961009	C	CTT	*RP1-186E20.1*	0.22	1.73 × 10^−8^	133	42	1	1
9	30346448	rs4879424	C	T	*snoU13*	0.15	2.18 × 10^−8^	190	169	1	1
10	110572259	rs1577857	G	T	*RP11-655H13.2*	0.26	2.47 × 10^−8^	122	102	1	1
2	27730940	rs1260326	C	T	*GCKR*	0.42	4.53 × 10^−8^	18	18	1	1
**African-ancestry GWAS**											
4	100229017	rs2066702	A	G	*ADH1B*	0.19	6.30 × 10^−17^	109	103	7	1
9	33094361	rs56175925	A	G	*B4GALT1*	0.02	1.83 × 10^−8^	6	5	1	1
**MTAG**											
4	100239319	rs1229984	T	C	*ADH1B*	0.04	3.35 × 10^−176^	1291	646	26	9
4	103188709	rs13107325	T	C	*SLC39A8*	0.08	6.01 × 10^−36^	75	26	6	1
2	27730940	rs1260326	T	C	*GCKR*	0.41	2.72 × 10^−21^	261	98	4	1
4	39423789	rs4975013	A	G	*KLB*	0.39	1.44 × 10^−19^	64	27	4	1
11	113364691	rs17602038	T	C	*DRD2*	0.37	2.62 × 10^−16^	70	36	3	2
11	47406592	rs11039216	T	C	*RP11-750H9.5*	0.47	1.07 × 10^−15^	519	245	5	1
17	44083402	rs1991556	A	G	*MAPT*	0.23	2.25 × 10^−15^	3854	2089	2	1
10	110572259	rs1577857	T	G	*RP11-655H13.2*	0.26	6.44 × 10^−15^	494	304	2	1
2	104361420	rs9308868	A	G	*AC013727.1*	0.49	4.88 × 10^−13^	518	329	3	1
14	58765903	rs61974485	T	C	*ARID4A*	0.28	1.02 × 10^−12^	117	5	1	1
2	45139904	rs472140	T	C	*RP11-89K21.1*	0.42	1.11 × 10^−11^	50	5	2	1
7	135119417	rs2551774	A	G	*CNOT4*	0.41	1.50 × 10^−11^	43	14	1	1
10	10688361	rs7910201	A	G	*RP1-186E20.1*	0.25	6.36 × 10^−11^	168	69	2	1
16	53834607	rs7188250	T	C	*FTO*	0.41	8.06 × 10^−10^	106	82	1	1
7	153487944	rs2098112	A	G	*DPP6*	0.47	8.99 × 10^−10^	112	2	1	1
19	49244219	rs2307018	A	C	*IZUMO1*	0.43	9.98 × 10^−10^	59	21	1	1
1	66503517	rs1354063	T	C	*PDE4B*	0.47	1.21 × 10^−9^	224	81	3	1
15	47681384	rs8033799	A	C	*CTD-2050N2.1*	0.21	1.86 × 10^−9^	157	65	2	1
5	153364650	rs2245405	A	G	*FAM114A2*	0.45	2.24 × 10^−9^	70	32	1	1
22	41789408	rs4822028	A	G	*TEF*	0.23	7.97 × 10^−9^	762	503	2	2
12	81602449	rs1921035	A	G	*ACSS3*	0.48	8.44 × 10^−9^	100	55	1	1
2	144225215	rs13024996	A	C	*RP11-570L15.2*	0.36	8.56 × 10^−9^	29	7	1	1
13	97019647	rs1925104	T	G	*HS6ST3*	0.33	1.37 × 10^−8^	100	56	1	1
8	21831385	rs1484162	A	G	*XPO7*	0.41	1.49 × 10^−8^	56	15	1	1
2	138197269	rs10496771	T	C	*THSD7B*	0.25	1.56 × 10^−8^	222	84	1	1
20	18546199	rs6045466	T	C	*LINC00493*	0.47	1.92 × 10^−8^	150	102	1	1
2	58042241	rs1402398	A	G	*CTD-2026C7.1*	0.39	2.00 × 10^−8^	268	55	2	1
7	117576675	rs6466637	A	G	*AC007568.1*	0.28	2.00 × 10^−8^	39	22	1	1
7	73015369	rs13240065	A	G	*MLXIPL*	0.12	2.95 × 10^−8^	66	46	1	1
2	204123832	rs56059523	T	C	*CYP20A1*	0.11	3.17 × 10^−8^	18	5	1	1
3	18793340	rs9835977	T	C	*AC144521.1*	0.30	3.57 × 10^−8^	79	44	1	1

Abbreviations: +, positive; –, negative; ?, not present; A, allele; Chr, chromosome; GWAS, genome-wide association studies; IS, independently significant; MAF, minor allele frequency; MTAG, multitrait analysis of genome-wide association studies; SNV, single nucleotide variant.

^a^ For the cross-ancestry GWAS, direction is shown; for European-ancestry GWAS, African-ancestry GWAS, and MTAG, MAF is shown.

The top association in the European-ancestry analysis was with the well-replicated functional *ADH1B* (OMIM 103720) rs1229984 variant (*P* = 3.12 × 10^−101^) (Table 1; eFigure 3 in Supplement 1). Additional genome-wide significant loci in the European ancestry analysis included *SLC39A8* (OMIM 608732; rs13107325; *P* = 6.78 × 10^−15^), *CRHR1* (OMIM 122561; rs77804065; *P* = 6.04 × 10^−14^), *AGBL2* (OMIM 617345; rs7107356; *P* = 1.43 × 10^−10^), and *GCKR* (OMIM 600842; rs1260326; *P* = 4.53 × 10^−8^). All loci in the European ancestry analysis remained genome-wide significant in the cross-ancestry meta-analysis, although there were some differences in the lead SNVs identified (Figure 1 and Table 1).

The top association in the African-ancestry analysis was with the known African-ancestry–specific functional locus at *ADH1B* (rs2066702; *P* = 6.30 × 10^−17^) (Table 1; eFigure 4 in Supplement 1). A second genome-wide significant association was found on chromosome 9 nearest *B4GALT1* (OMIM 137060; rs56175925; *P* = 1.83 × 10^−8^) (Figure 1 and Table 1).

In the cross-ancestry meta-analysis, 3 loci not identified in the ancestry-specific GWAS were genome-wide significant: rs826323, which maps to *ZNF420* (OMIM 617216; *P* = 1.39 × 10^−8^) on chromosome 19; rs6752675 on chromosome 2 nearest *AC092568**.1* (*P* = 2.57 × 10^−8^); and the *DRD2* (OMIM 126450) rs2514218 variant on chromosome 11 (*P* = 4.59 × 10^−8^) (Figure 1 and Table 1).

### Gene, Gene Set, and Tissue-Specific Gene Expression Analysis

Gene, gene set, and gene expression results are reported in the Supplement. Results can be found in eFigures 5 to 8 in Supplement 1 and eTables 2 to 7 in Supplement 2.

### SNV-Heritability and Genetic Correlations

LDSC estimated an observed-scale MaxAlc h^2^_SNV_ of 6.65% (SE, 0.41) in European-ancestry participants. LDSC estimates indicated that genome-wide inflation (λ_GC_ = 1.26) was likely indicative of the polygenic architecture of MaxAlc, as supported by an LDSC intercept of 0.99 (SE, 0.004) and attenuation ratio less than 0. For African-ancestry participants, COV-LDSC estimated h^2^_SNV _of 3.42% (SE, 1.46), a λ_GC _of 1.03, LDSC intercept of 0.99 (SE, 0.007), and attenuation ratio less than 0.

**Figure 2.**
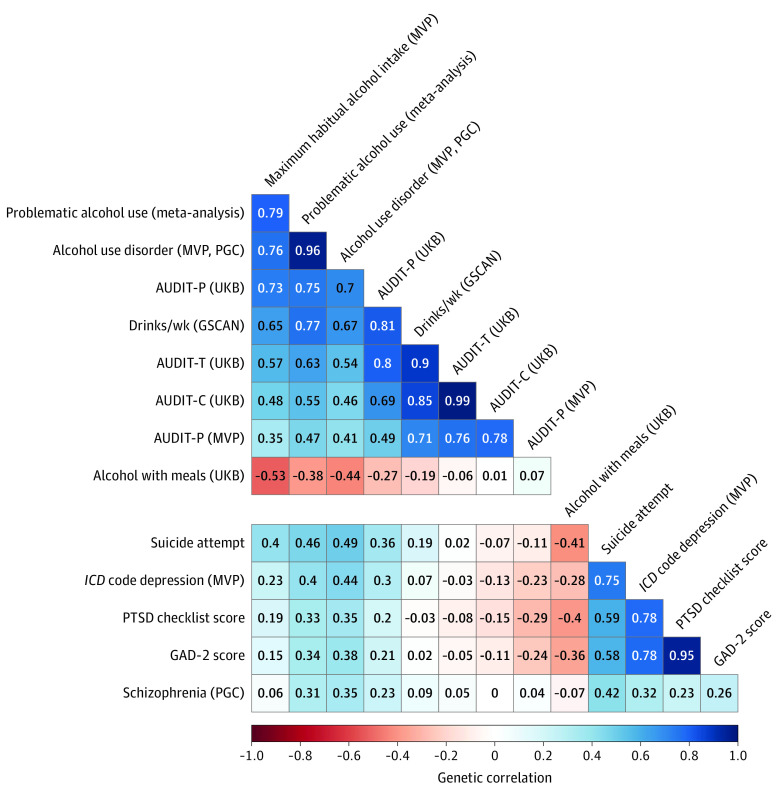
Genetic Correlations Between Million Veteran Program (MVP) Maximum Habitual Alcohol Intake, Alcohol Traits, and Psychiatric Outcomes AUDIT-C indicates Alcohol Use Disorders Identification Test–Consumption; AUDIT-P, Alcohol Use Disorders Identification Test–Problems; AUDIT-T, Alcohol Use Disorders Identification Test–Total; GAD-2, 2-item Generalized Anxiety Disorder; GSCAN, GWAS and Sequencing Consortium of Alcohol and Nicotine use; *ICD*, *International Classification of Diseases*; PGC, Psychiatric Genomics Consortium; PTSD, posttraumatic stress disorder; and UKB, UK Biobank.

In European-ancestry participants, LDSC estimated a high *r*_g_ for MaxAlc with PAU (*r*_g_ = 0.79; *P* = 3.95 × 10^−149^) and AUD (*r*_g_ = 0.76; *P* = 1.26 × 10^−127^), a lower *r*_g_ with consumption traits (MVP AUDIT-C, *r*_g_ = 0.35; *P* = 8.49 × 10^−15^; DPW, *r*_g_ = 0.65; *P* = 1.26 × 10^−103^), and a negative *r*_g_ with “alcohol usually taken with meals” (*r*_g_ = −0.53; *P* = 1.40 × 10^−50^) (Figure 2; eTable 8 in Supplement 2). Among the psychiatric traits examined, MaxAlc had the highest *r*_g_ with suicide attempt (*r*_g_ = 0.40; *P* = 3.02 × 10^−21^) (Figure 2; eTable 9 in Supplement 2). The MaxAlc *r*_g_ with suicide attempt was significantly higher than the *r*_g_ between suicide attempt and all other consumption measures but was not significantly different than the *r*_g_ between suicide attempt and AUD or PAU (eTable 9 in Supplement 2). The genetic correlations of MaxAlc with depression (*r*_g_ = 0.23; *P* = 8.40 × 10^−16^), PTSD (*r*_g_ = 0.19; *P* = 4.75 × 10^−5^), and anxiety (*r*_g_ = 0.15; *P* = 5.60 × 10^−3^) were also significantly different from those observed for alcohol consumption traits. No significant differences between MaxAlc and other consumption measures were observed for schizophrenia. Alcohol trait correlations with the core psychiatric traits included in the current study are reported in eTable 9 in Supplement 2.

### gSEM

EFA and CFA were conducted using gSEM for MaxAlc and a range of previously published alcohol traits ranging from AUD to alcohol consumption (AUDIT-C and DPW). Using EFA to examine different models showed that a common factor model did not adequately capture the associations across all alcohol traits (cumulative variance explained, 0.59) (eTable 10 in Supplement 2). The cumulative variance explained by the 2-factor model was 0.82 (factor 1, 0.42; factor 2, 0.40), with high sum of squared (SS) loadings for both factor 1 (SS, 2.91) and factor 2 (SS, 2.83). The 3-factor model explained less variance than the 2-factor model (cumulative variance, 0.80; factor 1, 0.35; factor 2, 0.32; factor 3, 0.14) and had SS loadings of 2.42 (factor 1), 2.23 (factor 2), and 0.98 (factor 3). Based on these findings, factor 3 was not worth retaining given that the 3-factor model accounted for less variance and the SS loading of factor 3 was less than 1.0. The 2-factor model best fit the data and was evaluated further using CFA (eTable 10 in Supplement 2).

**Figure 3.**
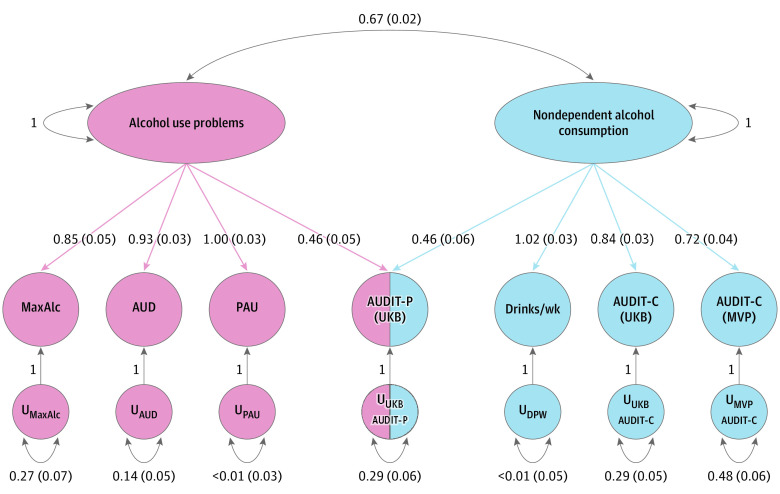
Genomic Structural Equation Modeling of Million Veteran Program (MVP) Maximum Habitual Alcohol Intake (MaxAlc) and Other Alcohol Use Traits Factor 1 (pink) was interpreted to capture alcohol use problems and factor 2 (blue), nondependent alcohol consumption. Values within the figure are loadings with SEs. AUD indicates alcohol use disorder; AUDIT-C, Alcohol Use Disorders Identification Test–Consumption; AUDIT-P, Alcohol Use Disorders Identification Test–Problems; PAU, problematic alcohol use; U, residual variance; UKB, UK Biobank.

CFA converged on a 2-factor model (correlation, 0.67) and demonstrated strong fit across the included alcohol traits (comparative fit index [CFI], 0.98; χ^2^ = 238.38; Aikake information criterion [AIC], 270.38; standardized root mean square residual [SRMR], 0.05) (Figure 3; eTable 11 in Supplement 2). MaxAlc loaded most strongly on factor 1 (loading, 0.85 [SE, 0.05]) along with AUD (loading, 0.93 [SE, 0.03]) and PAU (loading, 1.00 [SE, 0.03]). MVP AUDIT-C (loading, 0.72 [SE, 0.04]), UKB AUDIT-C (loading, 0.84 [SE, 0.03]), DPW (loading, 1.02 [SE, 0.03]), and AUDIT-P (loading, 0.46 [SE, 0.06]) loaded most strongly on factor 2, although AUDIT-P also loaded equally well on factor 1 (loading, 0.46 [SE, 0.05]).

### MR Analyses

Of the 3 liver enzyme levels examined (ALP, ALT, and GGT), only GGT was significantly genetically correlated with MaxAlc (*r*_g_ = 0.18; SE, 0.03; *P* = 1.17 × 10^−7^) (eTable 12 in Supplement 2). We thus focused MR analyses on the potential relationship between MaxAlc and GGT. Following the removal of 13 genetic variant outliers identified by MR-PRESSO, IVW methods indicated a small but statistically significant association between MaxAlc and GGT liver levels (β = 0.012; SE, 0.002; *P* = 2.66 × 10^−10^) (eFigures 9, 10, 11, and 12 in Supplement 1 and eTable 13 in Supplement 2). MR-RAPS results indicated that the effect estimates were not biased by the inclusion of SNVs of suggestive significance (eTable 14 in Supplement 2). Even after accounting for all SNV outliers, unexplained heterogeneity remained, potentially due to demographic differences between the 2 data sets used for the MR analysis (eTable 13 in Supplement 2).

### Multitrait Analysis of MaxAlc and PAU

The MaxAlc MTAG analysis provided an increase in the European ancestry MaxAlc sample size from 218 623 (GWAS mean χ^2^ = 1.29) to an equivalent sample size of 353 981 (MTAG mean χ^2^ = 1.47), resulting in 31 independent genome-wide significant MaxAlc loci. The top association was with *ADH1B *rs1229984 variant (*P* = 3.35 × 10^−176^)(Table 1; eFigure 13 in Supplement 1). Results for the MTAG analysis of PAU also showed increased locus discovery (eFigure 14 in Supplement 1) and are reported in eTables 15, 16, and 17 in Supplement 1). Circos plots for chromosomes containing identified loci in the respective MaxAlc MTAG and MaxAlc GWAS are reported in eFigures 15 and 16 in Supplement 1.

## Discussion

These findings highlight the additional information that can be gleaned from GWAS of habitual alcohol consumption traits that augment prior conclusions from GWAS of other alcohol consumption measures. We identified genome-wide significant associations with both novel and well-replicated alcohol use loci of relevance for heavy alcohol use (Table 2), observed differences in the genetic architecture of AUD and MaxAlc from that of other alcohol consumption traits, and provided insight into the genetic architecture of MaxAlc and its association to other psychiatric and medical conditions.

**Table 2.** Million Veteran Program MaxAlc Loci Summary Table^a^

Position	MaxAlc wave 2 GWS	Ancestry	Nearest gene	Novel locus	MaxAlc wave 1 GWS	Alcohol use	PAU	AUDIT-P	AUDIT-C	DPW	Locus previously GWS for other related outcomes
4:100239319	rs1229984	E	*ADH1B*	No	Yes	Yes	Yes	Yes	Yes	Yes	NA
4:100229017	rs2066702	A	*ADH1B*	No	Yes	Yes	No	No	No	No	NA
4:103188709	rs13107325	E	*SLC39A8*	No	No	Yes	Yes	Yes	Yes	Yes	Sleep, schizophrenia
17:43810896	rs77804065	E	*CRHR1*	N	Yes	No	No	No	No	Yes	Neuroticism, feeling guilty
11:47676170	rs7107356	E	*AGBL2*	Yes	No	No	No	No	No	No	Neuroticism, well-being, prefers bitter alcohol beverage
7:135162897	rs2551763	E	*CNOT4*	Yes	No	No	No	No	No	No	Educational attainment
1:7830262	rs2153733	E	*CAMTA1*	Yes	No	No	No	No	No	No	Morningness
19: 37504953	rs826323	C	*ZNF420*	Yes	No	No	No	No	No	No	Body shape, body mass index
10:10714452	rs145961009	E	*RP1-186E20.1*	Yes	No	No	No	No	No	No	NA
9:33094361	rs56175925	A	*B4GALT1*	Yes	No	No	No	No	No	No	Aspartate aminotransferase levels
9:30346448	rs4879424	E	*snoU13*	Yes	No	No	No	No	No	No	NA
10:110572259	rs1577857	E	*RP11-655H13.2*	No	Yes	No	Yes	No	No	No	NA
2:104056769	rs6752675	C	*AC092568.1*	Yes	No	No	No	No	No	No	NA
2:27730940	rs1260326	E	*GCKR*	No	No	Yes	Yes	No	Yes	Yes	NA
11:113392994	rs2514218	C	*DRD2*	No	No	Yes	Yes	No	No	No	Depression, neuroticism, well-being

Abbreviations: A, African ancestry; AUDIT-C, Alcohol Use Disorders Identification Test–Consumption; AUDIT-P, Alcohol Use Disorders Identification Test–Problems; C, cross-ancestry; DPW, drinks per week; E, European ancestry; GWS, genome-wide significance; MaxAlc, maximum habitual alcohol intake; NA, not applicable; PAU, problematic alcohol use.

^a^ GWS set at *P* = 5.0 × 10^−8^.

The strongest MaxAlc associations were with the 2 functional *ADH1B* loci—rs1229984 in the European-ancestry analysis and rs2066702 in the African-ancestry analysis. These *ADH1B* loci, among the few that survived from the candidate gene era,^1,33,34^ were also identified as risk loci for alcohol dependence in the respective ancestry groups in a genome-wide significant context.^35^ *ADH1B* has been associated with many alcohol traits studied using GWAS, including our previous MaxAlc study.^8^

MaxAlc loci at *ADH1B, CRHR1* (rs77804065), and *RP11-655H13.2* (rs1577857), previously identified in European-ancestry or African-ancestry participants, were confirmed in the present study, but the locus at *XPO7* (rs7821592), previously identified for MaxAlc in European-ancestry participants,^8^ fell below genome-wide significance; although *XPO7* was genome-wide significant in the MaxAlc MTAG analysis. Additional genome-wide significant associations included loci previously associated with alcohol consumption,^3,5,6^ PAU,^4^ and AUD^3^ in *SLC39A8* (rs13107325), *GCKR* (rs1260326), and *DRD2* (rs2514218). A summary of genome-wide significant associations from the MaxAlc GWAS, including previous associations with other alcohol traits and relevant outcomes, is presented.

We also identified novel associations (Table 2). For example, the MaxAlc European-ancestry GWAS identified a genome-wide significant association (rs7107356) near *AGBL2* (AGBL carboxypeptidase 2) on chromosome 11. *AGBL2* was previously identified as a locus for bitter alcoholic beverage taste preference.^36^ Decreased perception of bitterness can result in an increase in alcohol consumption,^37^ and genetic influences for bitter taste preference may influence alcoholic beverage intake.^38^

A novel finding in the MaxAlc African-ancestry GWAS was the genome-wide significant association near *B4GALT1* (BETA-1,4, galactrotransferase 1) on chromosome 9. *B4GALT1* is a member of the galactosyltransferase gene family and encodes an enzyme related to both glycoconjugate and lactose biosynthesis.^39^ Glycoconjugate metabolism occurring in the liver is often altered in the presence of chronic alcohol use, and glycoconjugate-related biomarkers are often considered markers of excessive alcohol use (differentiating alcohol-related vs non–alcohol-related tissue damage).^40^ These findings show how MaxAlc GWAS can provide novel insight into the biology of alcohol use, including in non–European-ancestry populations.

LDSC and gSEM results provided insight into differences and similarities in genetic architecture across a broad spectrum of alcohol consumption measures and AUD. Based on genetic correlations, MaxAlc is genetically more similar to AUD and PAU than to measures of alcohol consumption and is distinct from less problematic drinking styles such as the UK Biobank measure of “having alcohol with meals.” MaxAlc, similar to AUD and PAU, demonstrated significant differences in genetic correlation with many psychiatric outcomes compared with the largely negative or near zero genetic correlations between psychopathology and other alcohol consumption measures. The strongest genetic correlation between MaxAlc and psychiatric traits was with suicide attempt, which is consistent with the positive phenotypic association between heavy alcohol consumption and suicide behaviors.^41^

Our gSEM showed that a 2-factor solution best fit the data, including 1 factor that we suggest captures alcohol use problems and a second factor that captures nondependent alcohol consumption. MaxAlc loaded most strongly on the alcohol use problems factor, along with AUD, PAU, and AUDIT-P (which loaded equally on both factors). Thus, MaxAlc, a measure of habitual alcohol consumption, aligns with traits that capture alcohol use problems rather than episode-limited measures of quantity or frequency of alcohol use. The genetic association between alcohol consumption and AUD thus appears dependent on the steadiness and heaviness of alcohol consumption, which in turn appears to reflect dependent use.

The genetic study of MaxAlc is also positioned to study the impact of habitual alcohol use on alcohol-related disease, such as liver function. Understanding the causal role of problematic alcohol use on alcohol-related medical conditions is important. Previous genetic studies have focused primarily on examining the associations of AUD diagnosis with disease,^42^ although we know that harmful levels of drinking can negatively impact health outcomes even if not meeting AUD diagnostic criteria.^43^ The MR findings from the current study suggested significant genetic overlap and a small causal role of habitual drinking on GGT levels. Clinically, GGT elevations can be pronounced in individuals with a history of heavy drinking and those with alcohol-related cirrhosis and can be valuable for detecting alcohol-induced liver disease.^44^ Genetically informed studies of MaxAlc can help understand the biological and medical consequences of habitual drinking.

### Limitations

The current study has limitations. The MVP is a predominately male US military veteran sample, and the results reflect this demographic. Additionally, MaxAlc is a trait rather than a state measure, an advantage for genetic studies, but retrospective reports such as MaxAlc may be subject to longitudinal changes and reporting bias.^45^ MaxAlc also has sample size restrictions for non–European-ancestry participants, as is currently true for many genetic studies of complex traits.

## Conclusions

The present study is valuable in understanding relevant mechanisms involved as normative alcohol consumption approaches habitual problematic use, and ultimately, AUD.^46^ These findings suggest that MaxAlc is genetically different from other measures of alcohol consumption and that genetic studies of heavier drinking can be scientifically and medically informative in relation to broader substance use problems, the presence of psychiatric comorbidities, and identifying relationships with alcohol-related disease. These efforts provide promise for the continued advancement of our understanding of genetic influences across the spectrum of alcohol consumption levels and AUD and how these levels of alcohol use relate to other psychiatric and health conditions.

## Notes

Supplement 1. **eAppendix. **Supplemental Methods
eReferences.
**eFigure 1.** Screenshot of the MVP MaxAlc Survey Item Captured From Million Veteran Program Lifestyle Survey **eFigure 2.** Distribution of MVP Participant’s MaxAlc Survey Item Responses **eFigure 3.** Manhattan Plot of European Ancestry MaxAlc GWAS **eFigure 4.** Manhattan Plot of African Ancestry MaxAlc GWAS **eFigure 5.** Manhattan Plot of European Ancestry MaxAlc Gene-Based Results **eFigure 6.** Tissue Enrichment in European Ancestry MaxAlc Analysis **eFigure 7.** Manhattan Plot of African Ancestry MaxAlc Gene-Based Results **eFigure 8.** Tissue Enrichment in African Ancestry MaxAlc Analysis **eFigure 9.** Mendelian Randomization, Scatter Plot **eFigure 10.** Mendelian Randomization, Forest Plot **eFigure 11.** Mendelian Randomization, Leave-one-out Plot **eFigure 12.** Mendelian Randomization, Funnel Plot **eFigure 13.** Manhattan Plot of MaxAlc MTAG GWAS Results **eFigure 14.** Manhattan Plot of PAU MTAG GWAS Results **eFigure 15.** Circos Plots for Chromosomes Containing Genome-Wide Significant Loci for the MaxAlc GWAS **eFigure 16.** Circos Plots for Chromosomes Containing Genome-Wide Significant Loci for the MaxAlc MTAG GWAS

Supplement 2. **eTable 1. **MaxAlc MVP Participant Demographics Table **eTable 2.** MaxAlc European-Ancestry Gene Based Results **eTable 3.** MaxAlc European-Ancestry Gene Set Results **eTable 4.** MaxAlc European-Ancestry Tissue Enrichment Results **eTable 5.** MaxAlc African-Ancestry Gene Based Results **eTable 6.** MaxAlc African-Ancestry Gene Set Results **eTable 7.** MaxAlc African-Ancestry Tissue Enrichment Results **eTable 8.** MaxAlc Alcohol Trait Genetic Correlation Results **eTable 9.** MaxAlc Alcohol Psychiatric Trait Genetic Correlation Results **eTable 10.** Genomic-SEM Exploratory Factor Analysis (EFA) Results **eTable 11.** Genomic-SEM Confirmatory Factor Analysis (CFA) Results **eTable 12.** Mendelian Randomization (MR) MaxAlc Liver Enzyme Genetic Correlation Results **eTable 13.** Mendelian Randomization (MR) Results **eTable 14.** Mendelian Randomization Robust Adjusted Profile Score (MR-RAPs) Results **eTable 15.** MaxAlc Multi-trait Analysis of GWAS Results (with Problematic Alcohol Use) **eTable 16.** Problematic Alcohol Use Multitrait Analysis of GWAS Results (with MaxAlc) **eTable 17.** MaxAlc and Problematic Alcohol Use Multitrait Analysis of GWAS Results Comparison

Supplement 3.
Nonauthor Collaborators
