# Supplementary material for: Genome-Wide Investigation of Maximum Habitual Alcohol Intake in US Veterans in Relation to Alcohol Consumption Traits and Alcohol Use Disorder
Source: JAMA Netw Open. 2022 Oct 27;5(10):e2238880. doi: 10.1001/jamanetworkopen.2022.38880 (PMC9614582; doi:10.1001/jamanetworkopen.2022.38880)
Supplement: Supplement 1. — eAppendix. Supplemental Methods eReferences. eFigure 1. Screenshot of the MVP MaxAlc Survey Item Captured From Million Veteran Program Lifestyle Survey eFigure 2. Distribution of MVP Participant’s MaxAlc Survey Item Responses eFigure 3. Manhattan Plot of European Ancestry MaxAlc GWAS eFigure 4. Manhattan Plot of African Ancestry MaxAlc GWAS eFigure 5. Manhattan Plot of European Ancestry MaxAlc Gene-Based Results eFigure 6. Tissue Enrichment in European Ancestry MaxAlc Analysis eFigure 7. Manhattan Plot of African Ancestry MaxAlc Gene-Based Results eFigure 8. Tissue Enrichment in African Ancestry MaxAlc Analysis eFigure 9. Mendelian Randomization, Scatter Plot eFigure 10. Mendelian Randomization, Forest Plot eFigure 11. Mendelian Randomization, Leave-one-out Plot eFigure 12. Mendelian Randomization, Funnel Plot eFigure 13. Manhattan Plot of MaxAlc MTAG GWAS Results eFigure 14. Manhattan Plot of PAU MTAG GWAS Results eFigure 15. Circos Plots for Chromosomes Containing Genome-Wide Significant Loci for the MaxAlc GWAS eFigure 16. Circos Plots for Chromosomes Containing Genome-Wide Significant Loci for the MaxAlc MTAG GWAS [file jamanetwopen-e2238880-s001.pdf]

Supplemental Online Content

Deak JD, Levey DF, Wendt FR, et al; Million Veteran Program. Genome-wide investigation of maximum habitual alcohol intake in US veterans in relation to alcohol consumption traits and alcohol use disorder. *JAMA Netw Open.* 2022;5(10):e2238880.
doi:10.1001/jamanetworkopen.2022.38880

eAppendix. Supplemental Methods

eReferences.

eFigure 1. Screenshot of the MVP MaxAlc Survey Item Captured From Million Veteran Program Lifestyle Survey

eFigure 2. Distribution of MVP Participant's MaxAlc Survey Item Responses

eFigure 3. Manhattan Plot of European Ancestry MaxAlc GWAS

eFigure 4. Manhattan Plot of African Ancestry MaxAlc GWAS

eFigure 5. Manhattan Plot of European Ancestry MaxAlc Gene-Based Results

eFigure 6. Tissue Enrichment in European Ancestry MaxAlc Analysis

eFigure 7. Manhattan Plot of African Ancestry MaxAlc Gene-Based Results

eFigure 8. Tissue Enrichment in African Ancestry MaxAlc Analysis

eFigure 9. Mendelian Randomization, Scatter Plot

eFigure 10. Mendelian Randomization, Forest Plot

eFigure 11. Mendelian Randomization, Leave-one-out Plot

eFigure 12. Mendelian Randomization, Funnel Plot

eFigure 13. Manhattan Plot of MaxAlc MTAG GWAS Results

eFigure 14. Manhattan Plot of PAU MTAG GWAS Results

eFigure 15. Circos Plots for Chromosomes Containing Genome-Wide Significant Loci for the MaxAlc GWAS

eFigure 16. Circos Plots for Chromosomes Containing Genome-Wide Significant Loci for the MaxAlc MTAG GWAS

This supplemental material has been provided by the authors to give readers additional information about their work.

© 2022 Deak JD et al. *JAMA Network Open.*

eAppendix. Supplemental Methods

Description of included alcohol traits

Maximum habitual alcohol intake (MaxAlc) is defined from the question: "in a typical month, what is/was the largest number of drinks of alcohol you have had in one day." Ordinal response options ranged from 0 to ≥ 15 drinks. Some response options included a range of drinks (e.g., 5-6 drinks, ≥15 drinks) so individuals were categorized and analyzed with other participants reporting a MHAI within the same drinking range. In addition to MaxAlc, multiple additional genome-wide association studies (GWAS) of alcohol traits ranging from quantity and frequency of alcohol consumption to alcohol use disorder (AUD) were analyzed. This included a GWAS meta-analysis of AUD(1) that contained previously unpublished AUD cases and controls from the Million Veteran Program(MVP) defined by International Classification of Diseases (ICD) codes for AUD diagnosis combined with previously published AUD data(2) and alcohol dependence (AD) data(3). The GWAS of problematic alcohol use (PAU)(1) included the previously described AUD and AD data but also incorporated data from the alcohol use disorders identification test (AUDIT) subscale assessing alcohol problems (AUDIT-P) justified by strong genetic correlations across these PAU traits.

The AUDIT is a 10-item questionnaire developed to assess quickly for hazardous patterns of alcohol consumption and related alcohol problems(4). The AUDIT questionnaire can be split into multiple sub scores, including the first 3 items assessing patterns of alcohol consumption (the AUDIT-C), the final 7 items assessing alcohol-related problems (the AUDIT-P), and an overall total score aggregated across all 10 items (the AUDIT-T)(4). Separate GWAS of AUDIT-C scores were included from the MVP(2) and the UK Biobank(5), the latter of which also included GWAS of AUDIT-P and AUDIT-T scores in addition to the AUDIT-C(5).

The GWAS of drinks per week (DPW) was included from GSCAN (GWAS & Sequencing Consortium of Alcohol and Nicotine use)(6) that analyzed the average number of alcoholic beverages consumed in a week. The UK Biobank item of "alcohol usually taken with meals" (UK Biobank Field ID 1618) defined from the question: "when you drink alcohol, is it usually with meals."

Genotyping, imputation, and quality control

Genotyping and imputation for MVP have been described previously(2,7-11). Briefly, genotyping was performed using a custom Affymetrix Axiom Array covering ~720,000 SNPs. Imputation for SNPs was then performed using MiniMac4(12) and an African Genome Resources (AGR) reference panel curated by the Sanger Institute. Indels and complex variants were imputed separately using the 1000 Genomes Project phase 3 (1KG Phase 3) reference panel(13). Imputed variants with info score <0.30, genotype hard call missingness ≥0.20, and minor allele frequency <0.001 were removed. Eigenstrat(14) was used to conduct a principal components (PC) analysis to determine genetic ancestry using the 1kGP Phase 3 reference panel(13). MVP participants of European (EUR) and African (AFR) ancestry were identified by the first 10 PCs for inclusion in the current study.

Gene, gene-set, and tissue-specific gene expression analysis

FUMA (Functional Mapping and Annotation)(15) was used to conduct gene-based, gene-set, and tissue-specific gene expression analyses using MAGMA (Multi-marker Analysis of GenoMic Annotation)(16). SNPs were mapped to 18,723 genes for EUR and 19,024 for AFR. Ancestry-specific Bonferroni corrections were used to determine GWS (EUR: p=0.05/18,723=2.67x10^{-06}; AFR: p=0.05/19,024 = 2.63x10^{-06}) for gene-based tests. MAGMA

was also used to conduct analyses of gene sets classified based on gene function and biological processes (MsigDB). Tissue-specific gene expression was also analyzed using tissue transcriptomic profile data from GTEx v8(17).

In EUR, 27 genes reached Bonferroni-corrected GWS (p=2.67x10^{-06}) in the gene-based test (**eFigure5; eTable2**). The top gene-based association was with *KANSL1* (p=8.67x10^{-14}) on chromosome 17. Thirteen additional genes, including *CRHR1*, map in close proximity to *KANSL1* in a well-known inversion region on chromosome 17 and were also GWS. No gene sets were GWS in the EUR analysis (**eTable3**). Multiple brain regions were enriched in the EUR tissue-expression analysis including cerebellum (p=8.25x10^{-06}), cerebellar hemisphere (p=8.46x10^{-06}), frontal cortex (p=5.05x10^{-05}), brain cortex (p=2.19x10^{-04}), anterior cingulate cortex (p=2.19x10^{-04}), hypothalamus (p=2.36x10^{-04}), and the nucleus accumbens basal ganglia (p=5.46x10^{-04})(**eFigure6; eTable4**).

In AFR, *ADH1B* (p=3.28x10^{-08}) and *METAP1* (p=7.11x10^{-07}) on chromosome 4 were GWS in the gene-based test (**eFigure7; eTable5**). The AFR gene-set analysis resulted in two GWS gene sets: GO_bp:go_retinoic_acid_metabolic_process (p=2.62x10^{-08};$p_{bonferr}$=4.05x10^{-04}) and GO_mf:go_alcohol_dehydrogenase_activity_zinc_dependent (p=1.95x10^{-07};$p_{bonferr}$=3.01x10^{-03}) (**eTable6**). There were no significant tissue expression findings in the AFR MHAI analysis (**eFigure8; eTable7**).

Functional characterization of identified genetic risk loci

Genetic variants were characterized using two gene-mapping approaches: (1) by using expression quantitative trait locus (eQTL) data from GTEx v8(17) and BRAINEAC(18); and (2)

© 2022 Deak JD et al. *JAMA Network Open.*

using 3D chromatin interactions (Hi-C)(19). Both approaches were implemented in the FUMA platform (Functional Mapping and Annotation)(15).

Gene-mapping using eQTL data was performed using GTEx v8 gene expression data including gene expression data for: amygdala, anterior cingulate cortex BA24, cerebellar hemisphere, cerebellum, cortex, frontal cortex BA9, hippocampus, hypothalamus, nucleus accumbens basal ganglia, putamen basal ganglia, spinal cord cervical c-1, and substantia nigra(17). BRAINEAC(18) tissues included: cerebellar cortex, frontal cortex, hippocampus, inferior olivary nucleus, occipital cortex, putamen, substantia nigra, temporal cortex, thalamus, and intralobular white matter. Hi-C chromatin interaction data included PsychENCODE(20) EP links and PsychENCODE promoter anchored loops, and FUMA-based datasets for Hi-C in adult cortex, dorsolateral prefrontal cortex, and hippocampus. Circos plots for chromosomes containing genome-wide significant MHAI loci are presented in **eFigures 15-16**.

Multi-trait analysis of MHAI and problematic alcohol use (PAU)

A multi-trait analysis of GWAS (MTAG)(16) was performed using summary statistics from the EUR MHAI GWAS and the previously published GWAS of PAU(1). MHAI and PAU were strongly genetically correlated (rg=0.79). MTAG leverages the high degree of genetic correlation between related traits to generate trait-specific effect estimates for each genetic variant, and thus, can enhance statistical power for trait-specific genetic discovery through the inclusion of multiple GWAS summary statistics while also accounting for any sample overlap(21).

The MTAG analysis was restricted to SNPs in common to both the EUR MHAI GWAS (N=218,623) and the PAU GWAS ($N_{effective}$=300,789), with a minor allele frequency > 0.01, and

© 2022 Deak JD et al. *JAMA Network Open.*

occurring in at least 50% of the effective sample size of the GWAS summary statistics. Because information from both sets of GWAS summary statistics are informative for the other included trait, effectively serving to boost the power of each respective study through the inclusion of the other, MTAG generates both MaxAlc-specific and PAU-specific effect estimates for all included SNPs. Thus, MTAG results are presented for both the MHAI trait and the PAU trait.

The top association in the MHAI MTAG analysis was with *ADH1B* rs1229984 (p=3.35x10^{-176}). Many additional variants of interest were also identified, including the *XPO7* gene (rs1484162; p=1.49x10^{-08}) that was genome-wide significant in the initial MHAI GWAS(2); however, dropped below GWS in the EUR MHAI GWAS in the current study (**eFigure13; Table 1; eTable15 [includes MTAG effect estimates]**).

The MTAG analysis for PAU resulted in a jump in sample size from a maximum effective sample size of N=300,789 (GWAS mean χ^2=1.35) to an equivalent sample size of N=422,491 (MTAG mean χ^2=1.49). The PAU-specific MTAG resulted in 42 independent GWS PAU risk loci. The top PAU association was also with *ADH1B* rs1229984 (p=2.38x10^{-182}) (**eFigure14; eTable16**). Overlap between the respective MHAI and PAU MTAG analyses are reported in **eTable17**.

eReferences

1. Zhou H, Sealock JM, Sanchez-Roige S, et al. Genome-wide meta-analysis of problematic alcohol use in 435,563 individuals yields insights into biology and relationships with other traits. *Nature Neuroscience*. 2020;23(7):809-818. doi:10.1038/s41593-020-0643-5.
2. Kranzler HR, Zhou H, Kember RL, et al.. Genome-wide association study of alcohol consumption and use disorder in 274,424 individuals from multiple populations. *Nature Communications*. 2019;10(1). doi:10.1038/s41467-019-09480-8.
3. Walters RK, Polimanti R, Johnson EC, et al.. Transancestral GWAS of alcohol dependence reveals common genetic underpinnings with psychiatric disorders. Nature Neuroscience. 2018;21(12):1656-1669. doi:10.1038/s41593-018-0275-1.
4. Saunders JB, Aasland OG, Babor TF, De La Fuente JR, Grant M. Development of the Alcohol Use Disorders Identification Test (AUDIT): WHO Collaborative Project on Early Detection of Persons with Harmful Alcohol Consumption-II. *Addiction*. 1993;88(6):791-804. doi:10.1111/j.1360-0443.1993.tb02093.x.
5. Sanchez-Roige S, Palmer AA, Fontanillas P, et al. Genome-Wide Association Study Meta-Analysis of the Alcohol Use Disorders Identification Test (AUDIT) in Two Population-Based Cohorts. *American Journal of Psychiatry*. 2019;176(2):107-118. doi:10.1176/appi.ajp.2018.18040369.
6. Liu M, Jiang Y, Wedow R, et al. Association studies of up to 1.2 million individuals yield new insights into the genetic etiology of tobacco and alcohol use. *Nature Genetics*. 2019;51(2):237-244. doi:10.1038/s41588-018-0307-5.

© 2022 Deak JD et al. *JAMA Network Open.*

7. Gaziano JM, Concato J, Brophy M, et al. Million Veteran Program: A mega-biobank to study genetic influences on health and disease. *Journal of Clinical Epidemiology*. 2016;70:214-223. doi:10.1016/j.jclinepi.2015.09.016.

8. Gelernter J, Sun N, Polimanti R, et al. Genome-wide Association Study of Maximum Habitual Alcohol Intake in >140,000 U.S. European and African American Veterans Yields Novel Risk Loci. *Biological Psychiatry*. 2019;86(5):365-376. doi:10.1016/j.biopsych.2019.03.984.

9. Levey DF, Gelernter J, Polimanti R, et al. Reproducible Genetic Risk Loci for Anxiety: Results From ~200,000 Participants in the Million Veteran Program. *American Journal of Psychiatry*. 2020;177(3):223-232. doi:10.1176/appi.ajp.2019.19030256.

10. Levey DF, Stein MB, Wendt FR, et al. Bi-ancestral depression GWAS in the Million Veteran Program and meta-analysis in >1.2 million individuals highlight new therapeutic directions. *Nature Neuroscience*. 2021;24(7):954-963. doi:10.1038/s41593-021-00860-2.

11. Stein MB, Levey DF, Cheng Z, et al. Genome-wide association analyses of post-traumatic stress disorder and its symptom subdomains in the Million Veteran Program. *Nature Genetics*. 2021;53(2):174-184. doi:10.1038/s41588-020-00767-x.

12. Das S, Forer L, Schönherr S, et al. Next-generation genotype imputation service and methods. *Nature Genetics*. 2016;48(10):1284-1287. doi:10.1038/ng.3656.

13. The 1000 Genomes Project Consortium. A global reference for human genetic variation. *Nature*. 2015;526(7571):68-74. doi:10.1038/nature15393.

© 2022 Deak JD et al. *JAMA Network Open.*

14. Price AL, Patterson NJ, Plenge RM, Weinblatt ME, Shadick NA, Reich D. Principal components analysis corrects for stratification in genome-wide association studies. *Nature Genetics*. 2006;38(8):904-909. doi:10.1038/ng1847.

15. Watanabe K, Taskesen E, Van Bochoven A, Posthuma D. Functional mapping and annotation of genetic associations with FUMA. *Nature Communications*. 2017;8(1). doi:10.1038/s41467-017-01261-5.

16. De Leeuw CA, Mooij JM, Heskes T, Posthuma D. MAGMA: Generalized Gene-Set Analysis of GWAS Data. *PLOS Computational Biology*. 2015;11(4):e1004219. doi:10.1371/journal.pcbi.1004219.

17. GTEx Consortium. Genetic effects on gene expression across human tissues. *Nature*. 2017;550(7675):204-213. doi:10.1038/nature24277.

18. Ramasamy A, Trabzuni D, Guelfi S, Varghese V, Smith C, Walker R (2014): Genetic variability in the regulation of gene expression in ten regions of the human brain. *Nature Neuroscience*. 17: 1418–1428.

19. Anthony, Hu M, Jung I, et al.. A Compendium of Chromatin Contact Maps Reveals Spatially Active Regions in the Human Genome. *Cell Reports*. 2016;17(8):2042-2059. doi:10.1016/j.celrep.2016.10.061.

20. Akbarian S, Liu C, Knowles JA, Vaccarino FM, Farnham PJ, Crawford GE (2015): The PsychENCODE project. *Nature Neuroscience*. 18: 1707–1712.

21. Turley P, Walters RK, Maghzian O, et al. Multi-trait analysis of genome-wide association summary statistics using MTAG. *Nature Genetics*. 2018;50(2):229-237. doi:10.1038/s41588-017-0009-4.

© 2022 Deak JD et al. *JAMA Network Open.*

eFigure 1. Screenshot of the MVP MHAI Survey Item Captured From Million Veteran Program Lifestyle Survey

19. In a typical month, what is/was the largest number of drinks of alcohol (beer, wine and/or liquor) you may have had in one day?

- ☐ None
- ☐ 1 drink
- ☐ 2 drinks
- ☐ 3 drinks
- ☐ 4 drinks
- ☐ 5 - 6 drinks
- ☐ 7 - 9 drinks
- ☐ 10 - 14 drinks
- ☐ 15 or more drinks

© 2022 Deak JD et al. *JAMA Network Open.*

eFigure 2. Distribution of MVP Participant's MHAI Survey Item Responses

(a) all included participants; (b) AFR participants only; (c) EUR participants only.

(a)

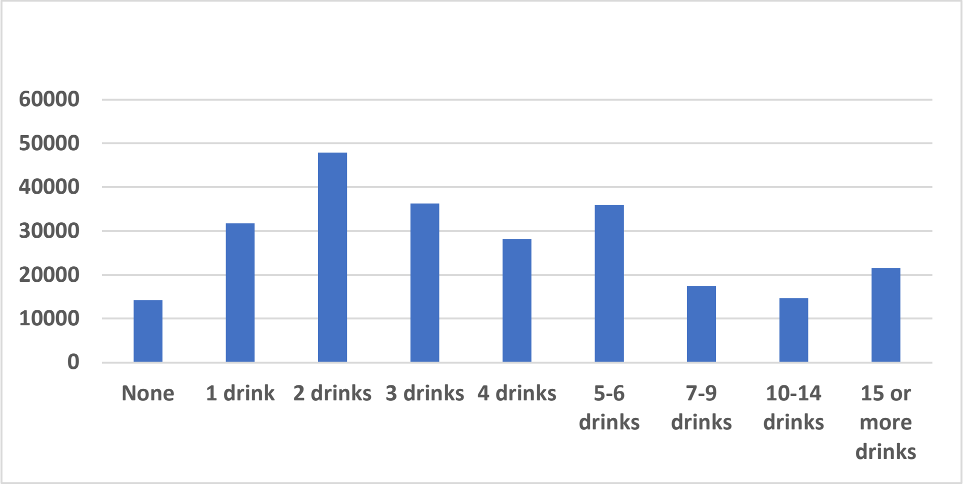

(b)

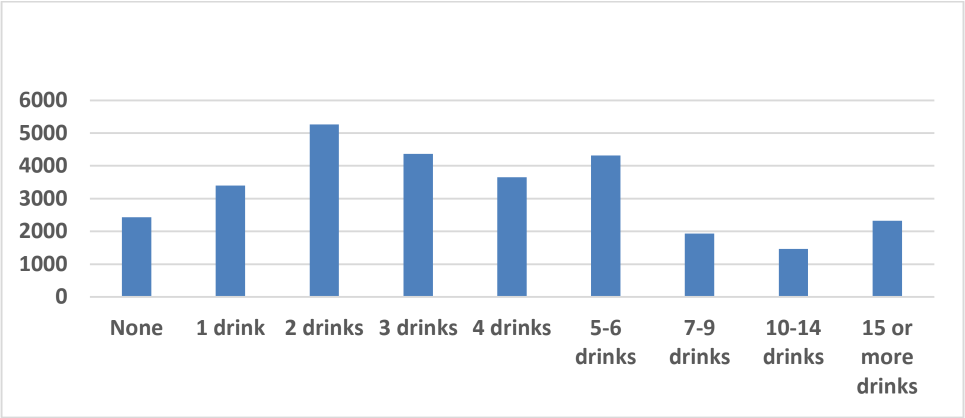

(c)

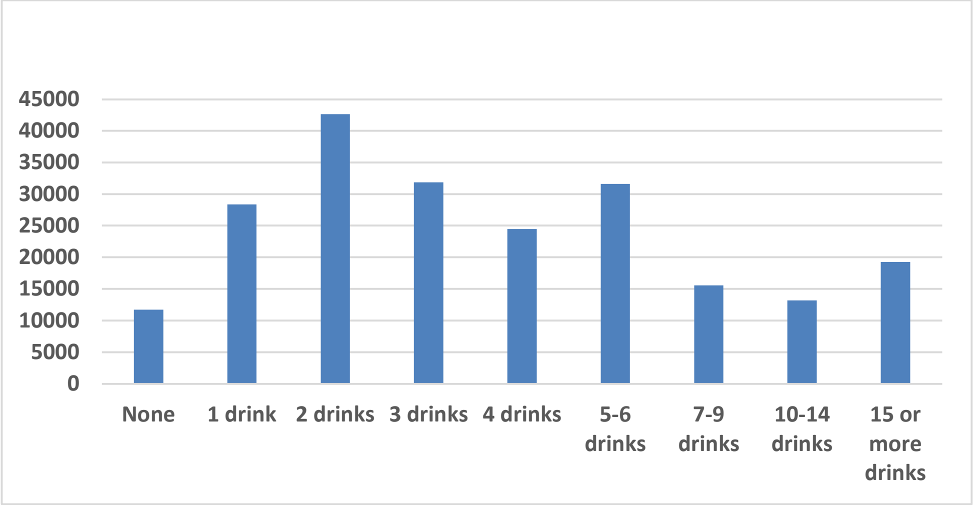

© 2022 Deak JD et al. *JAMA Network Open.*

eFigure 3. Manhattan Plot of European Ancestry MHAI GWAS

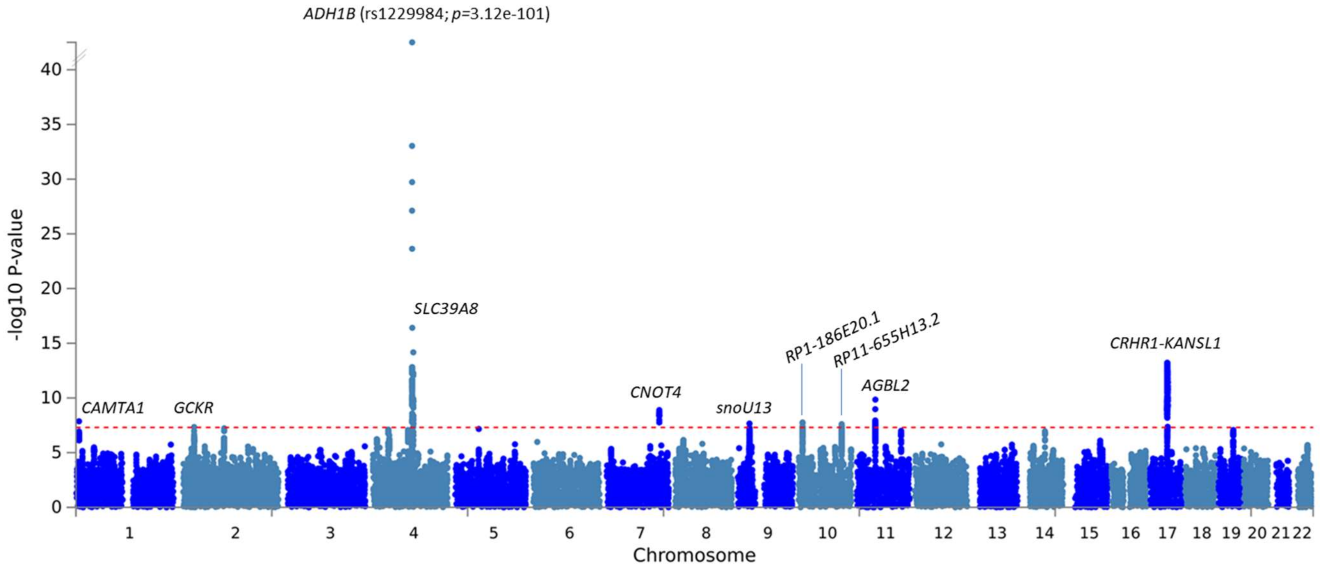

© 2022 Deak JD et al. *JAMA Network Open.*

eFigure 4. Manhattan Plot of African Ancestry MHAI GWAS

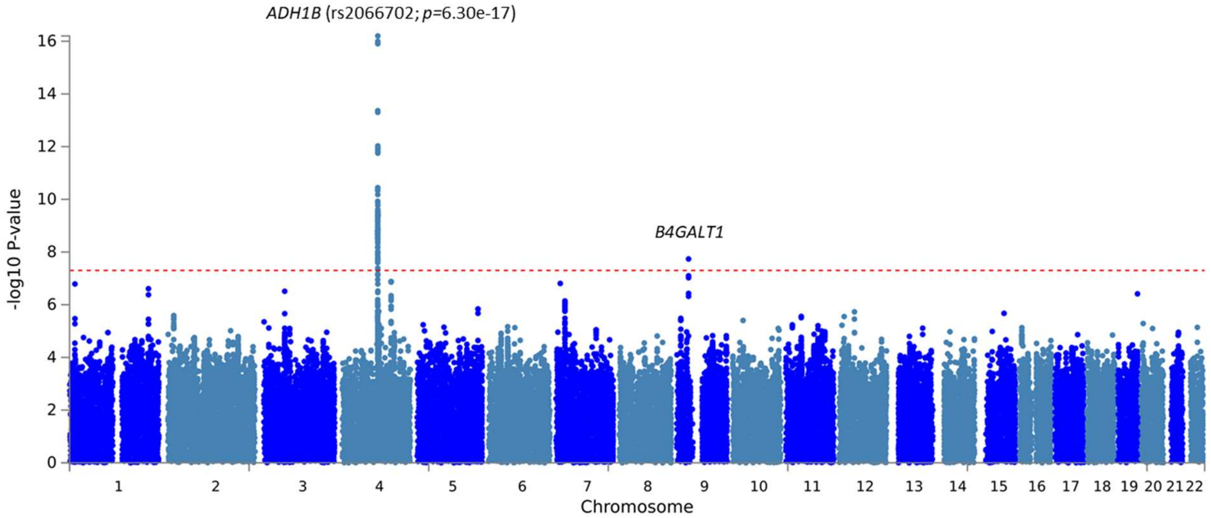

© 2022 Deak JD et al. *JAMA Network Open.*

eFigure 5. Manhattan Plot of European Ancestry MHAI Gene-Based Results

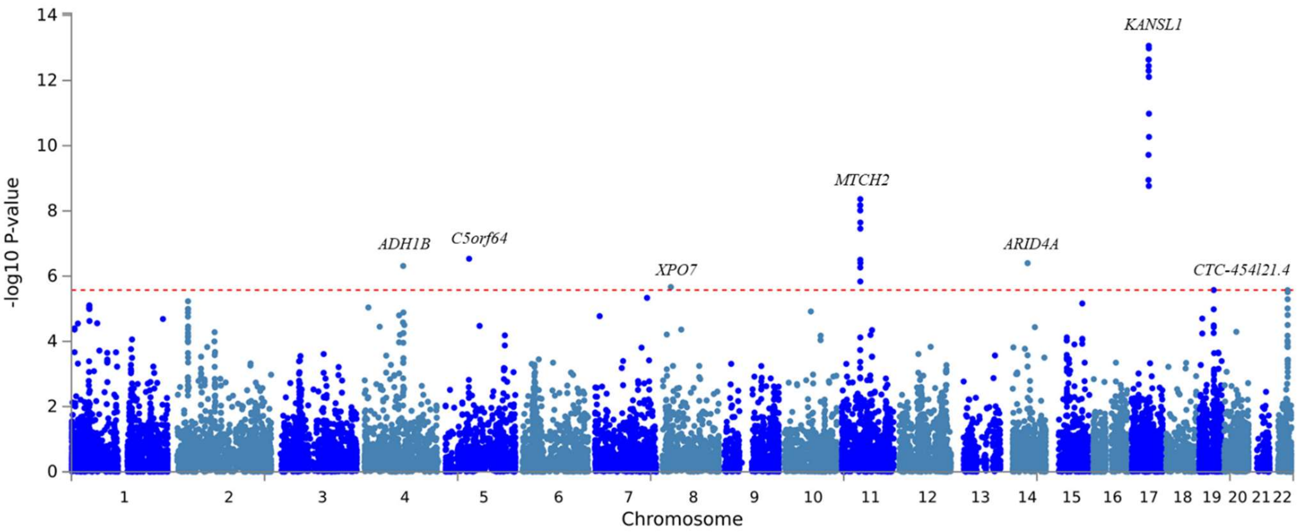

© 2022 Deak JD et al. *JAMA Network Open.*

eFigure 6. Tissue Enrichment in European Ancestry MHAI Analysis

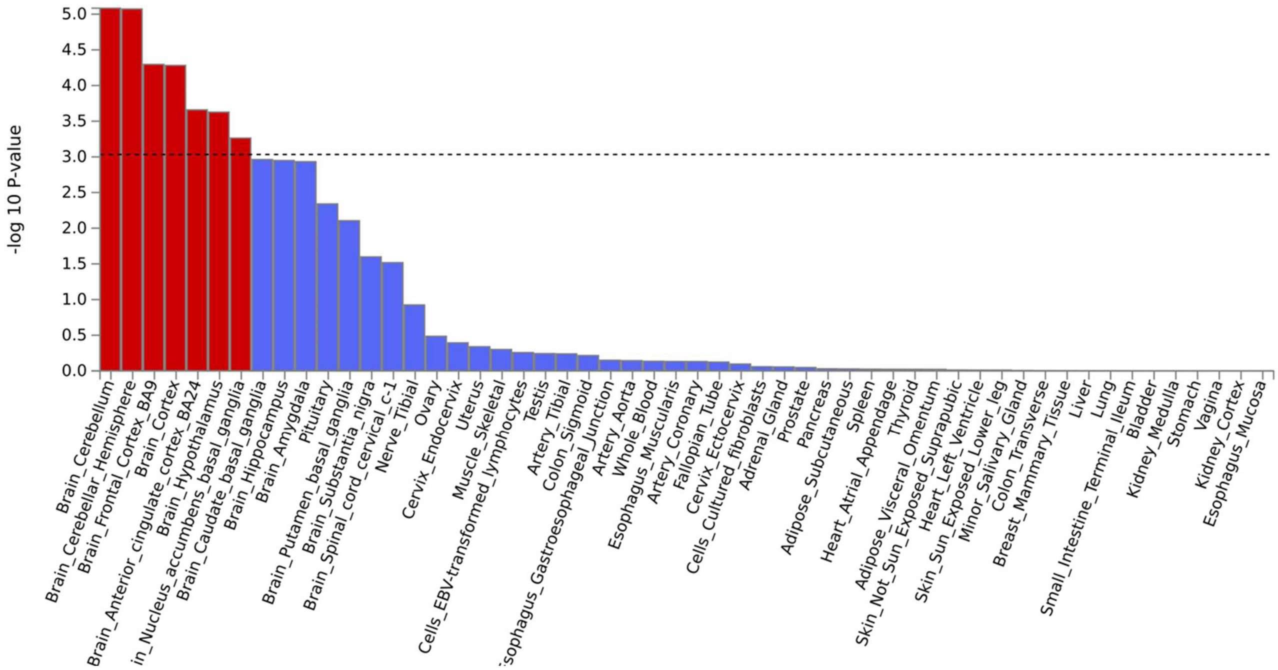

© 2022 Deak JD et al. *JAMA Network Open.*

eFigure 7. Manhattan Plot of African Ancestry MHAI Gene-Based Results

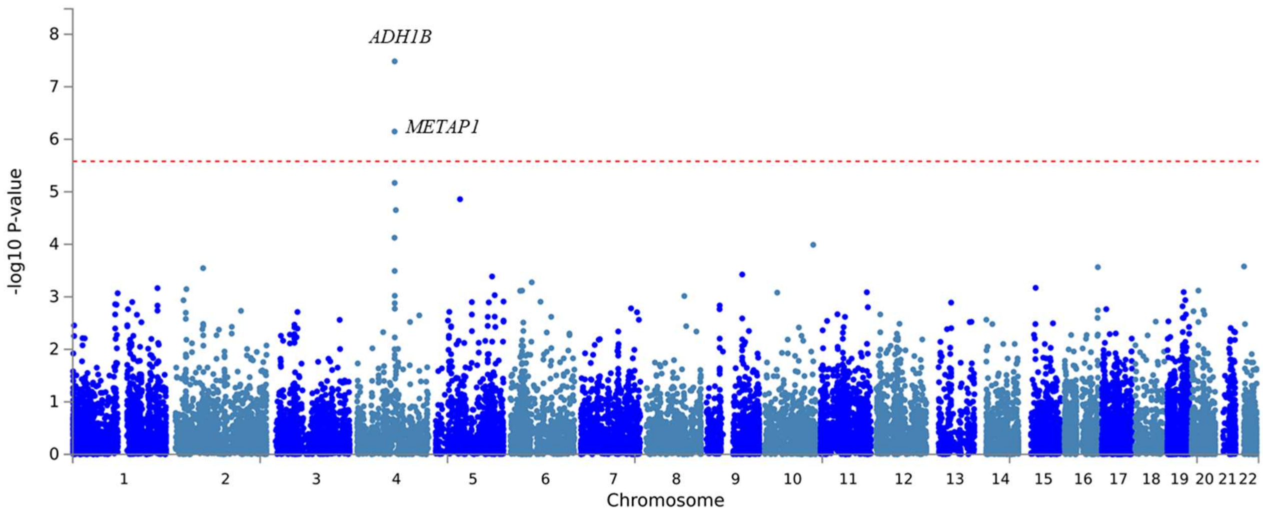

© 2022 Deak JD et al. *JAMA Network Open.*

eFigure 8. Tissue Enrichment in African Ancestry MHAI Analysis

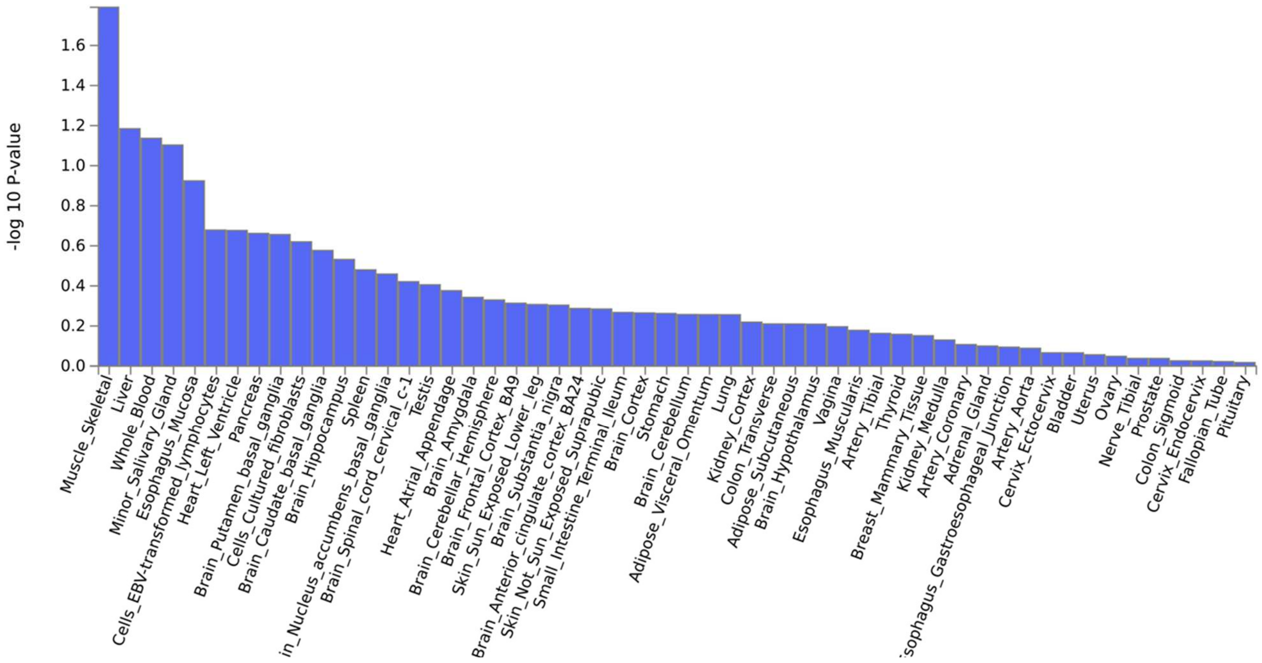

© 2022 Deak JD et al. *JAMA Network Open.*

eFigure 9. Mendelian Randomization, Scatter Plot

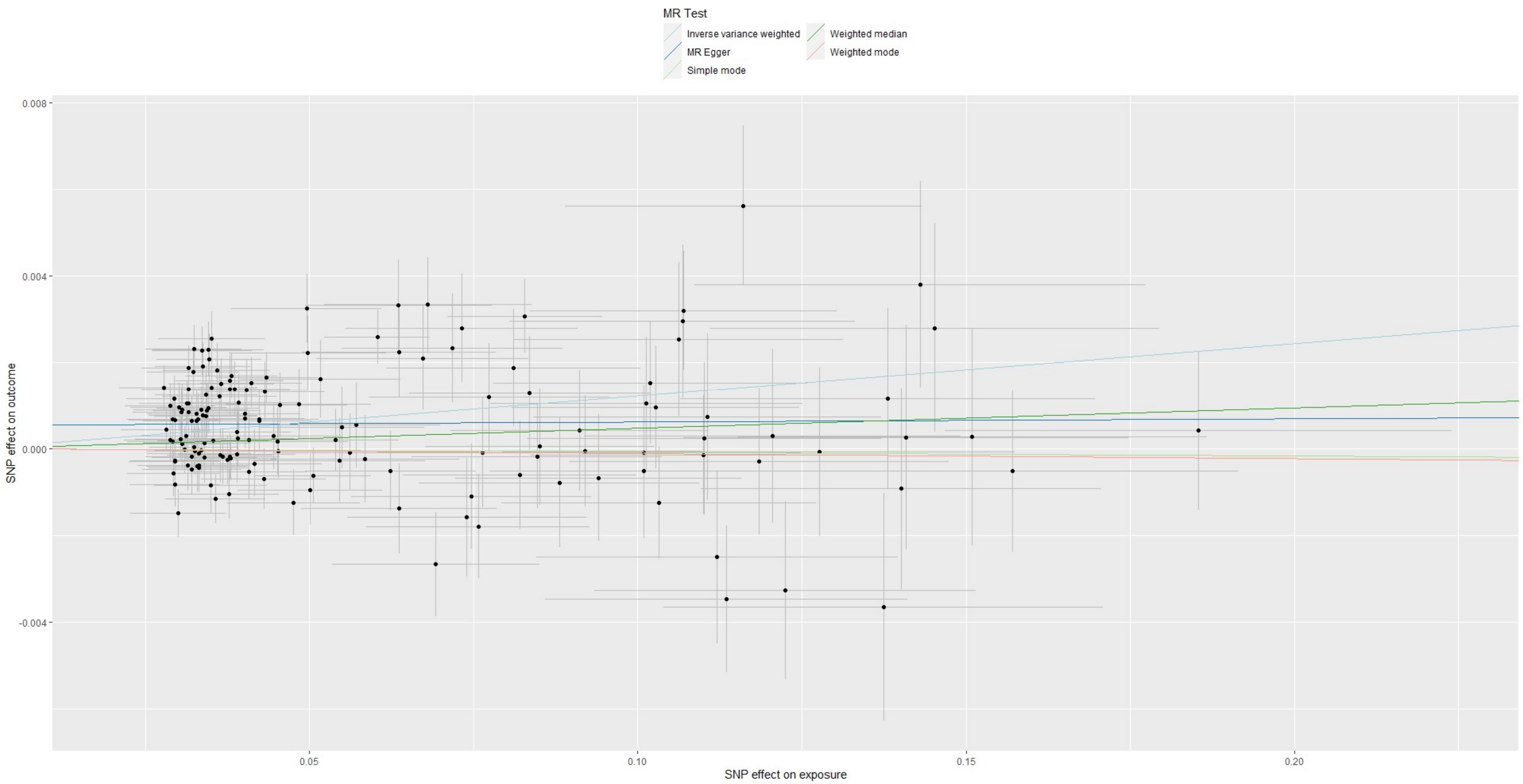

© 2022 Deak JD et al. *JAMA Network Open.*

eFigure 10. Mendelian Randomization, Forest Plot

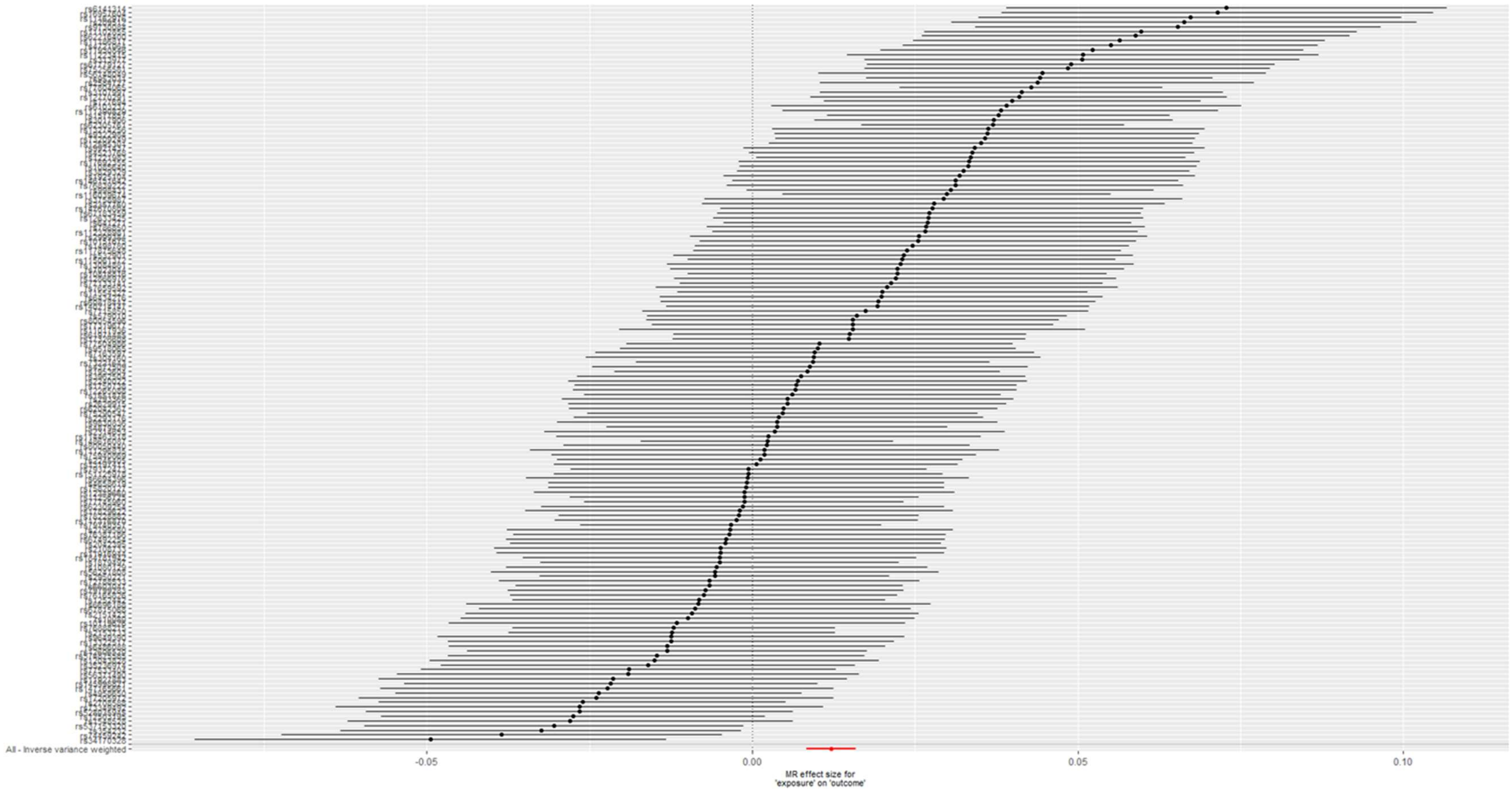

© 2022 Deak JD et al. *JAMA Network Open.*

eFigure 11. Mendelian Randomization, Leave-one-out Plot

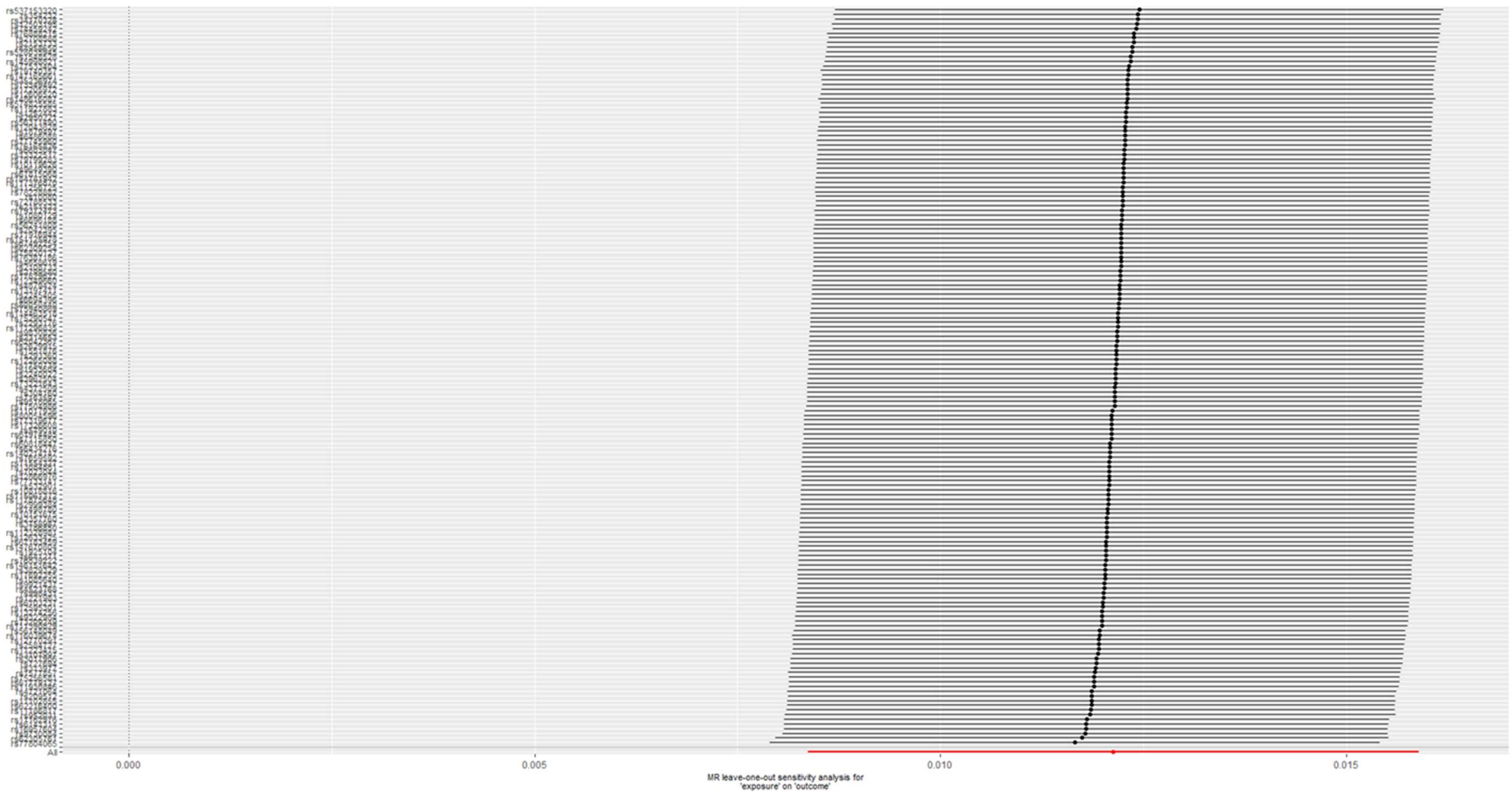

© 2022 Deak JD et al. *JAMA Network Open.*

eFigure 12. Mendelian Randomization, Funnel Plot

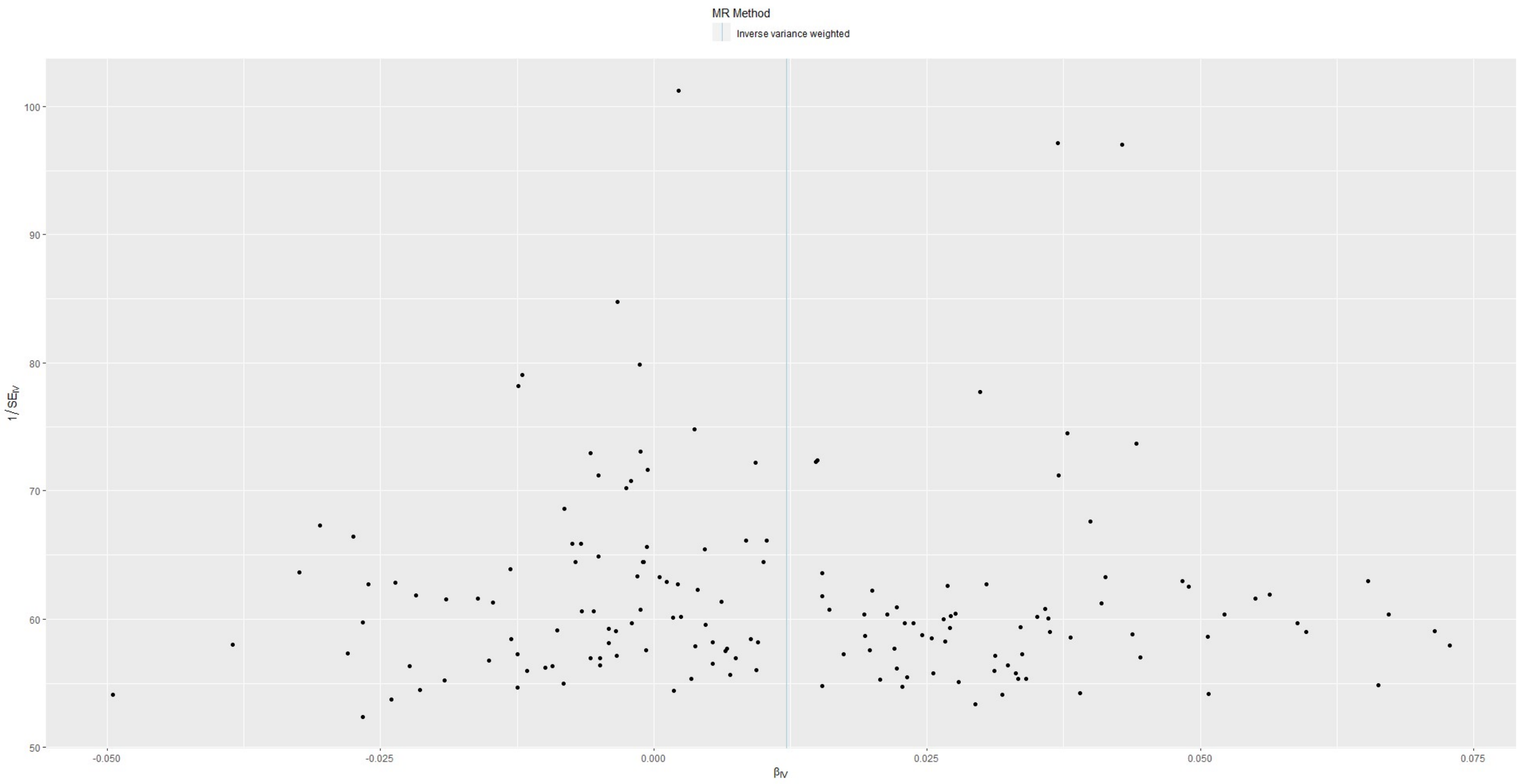

© 2022 Deak JD et al. *JAMA Network Open.*

eFigure 13. Manhattan Plot of MHAI MTAG GWAS Results

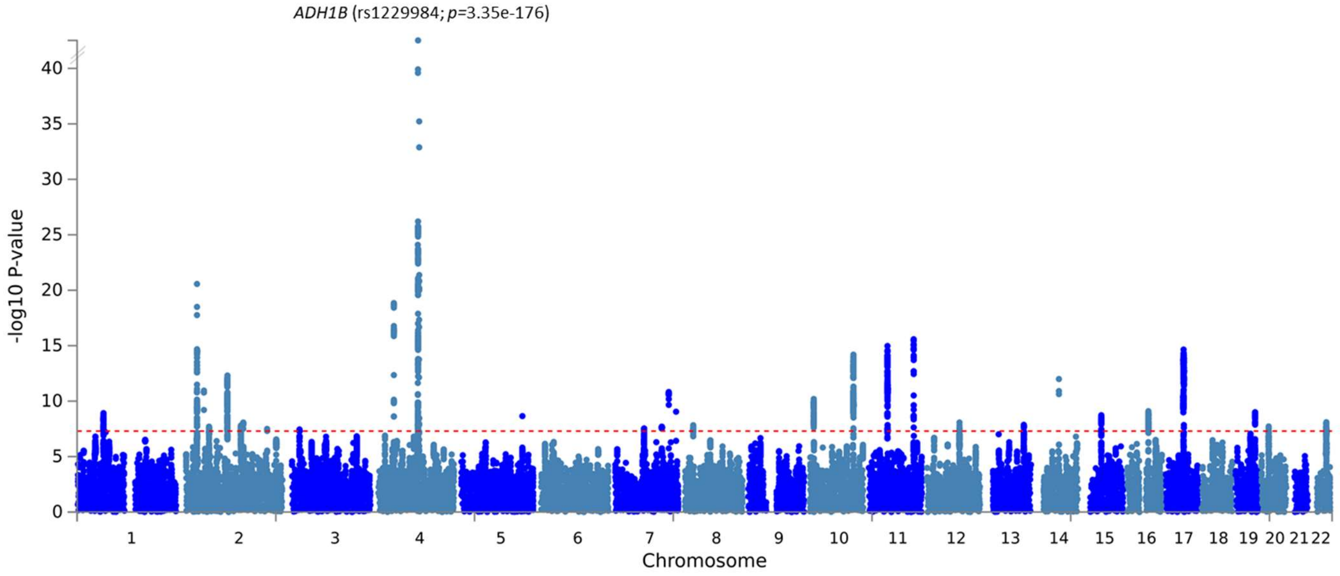

© 2022 Deak JD et al. *JAMA Network Open.*

eFigure 14. Manhattan Plot of PAU MTAG GWAS Results

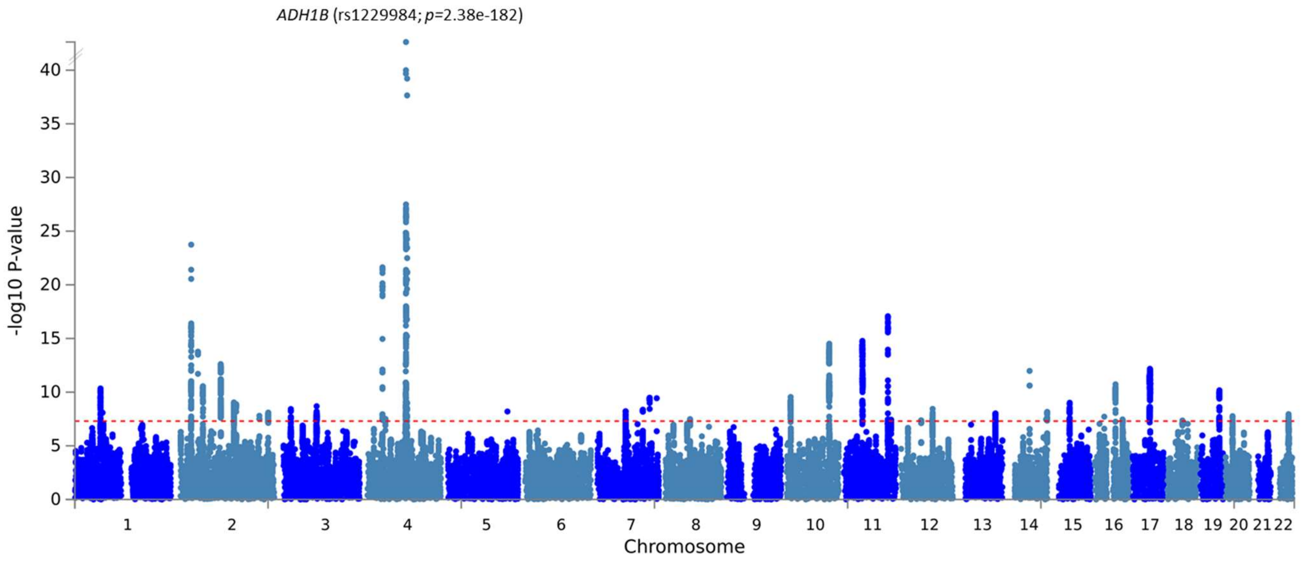

© 2022 Deak JD et al. *JAMA Network Open.*

eFigure 15. Circos Plots for Chromosomes Containing Genome-Wide Significant Loci for the MHAI GWAS

Note. **Outer most layer** is a Manhattan plot of genome-wide association study (GWAS) single-nucleotide polymorphisms (SNPs) with $p \leq 0.05$. SNPs are plotted by chromosomal position along the *x*-axis with their corresponding $-\log^{-10}$ *p*-value on the *y*-axis. Linkage-disequilibrium (LD) between the identified lead SNP and surrounding SNPs is indicated from $r^2 > 0.8$ (red), $r^2 > 0.6$ (orange), $r^2 > 0.4$ (green), $r^2 > 0.2$ (blue). SNPs that are not in LD with the lead SNP ($r^2 \leq 0.02$) are gray. **Second layer (chromosome ring):** Chromosomal regions containing identified genomic risk loci are colored in blue. The names of genes implicated based upon variant associations with brain tissue expression quantitative trait loci (eQTLs) are colored green. The names of genes implicated based upon 3D chromatin interactions (Hi-C) are colored orange. Genes that are mapped based upon both eQTLs and Hi-C associations are colored red. **Third layer (chromosome ring):** Variants mapped to genes based upon associations with brain tissue eQTLs are linked in green. Variants mapped to genes based upon Hi-C data are linked in orange.

© 2022 Deak JD et al. *JAMA Network Open.*

(a) Chromosome 1

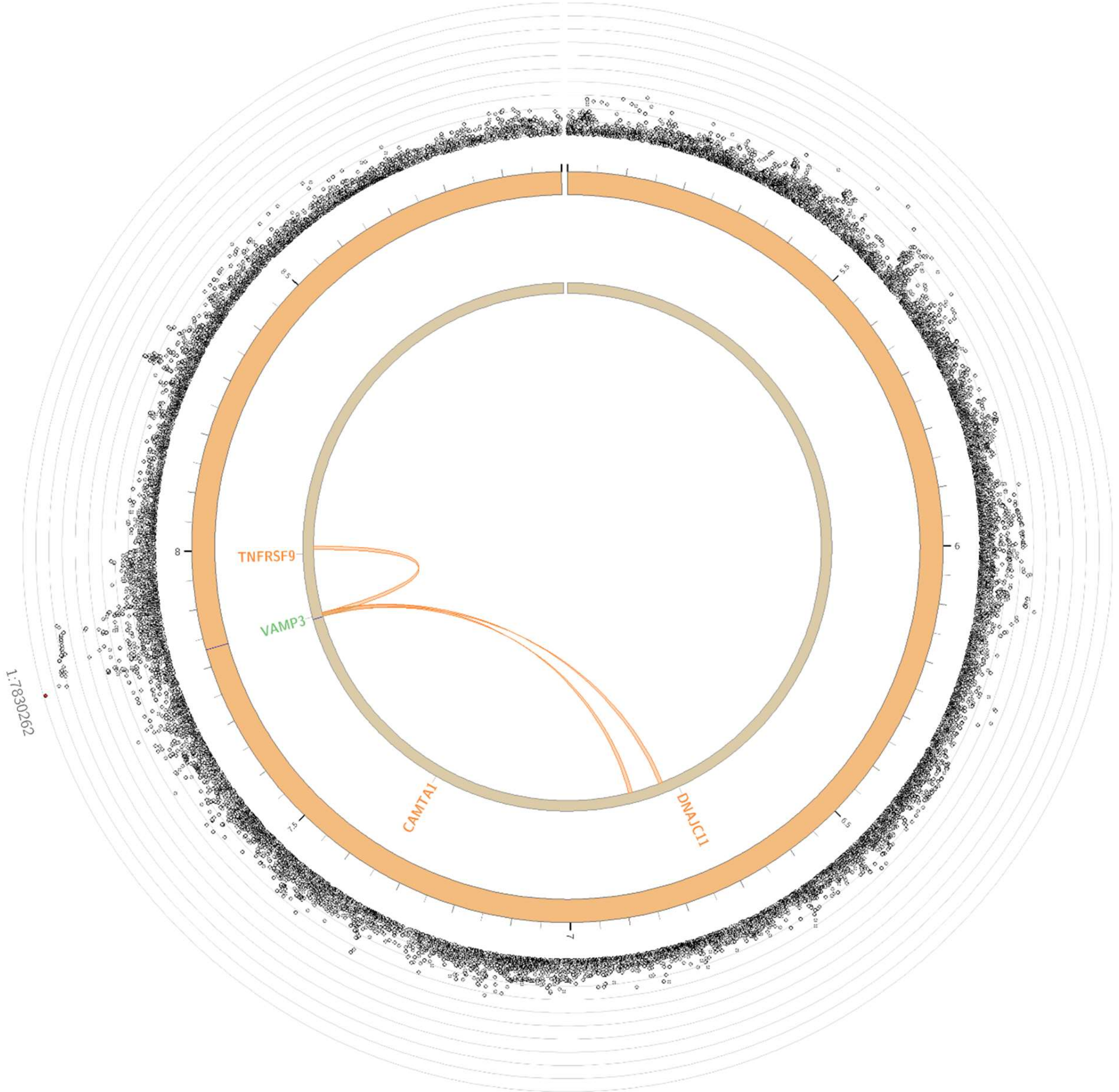

© 2022 Deak JD et al. *JAMA Network Open.*

(b) Chromosome 2

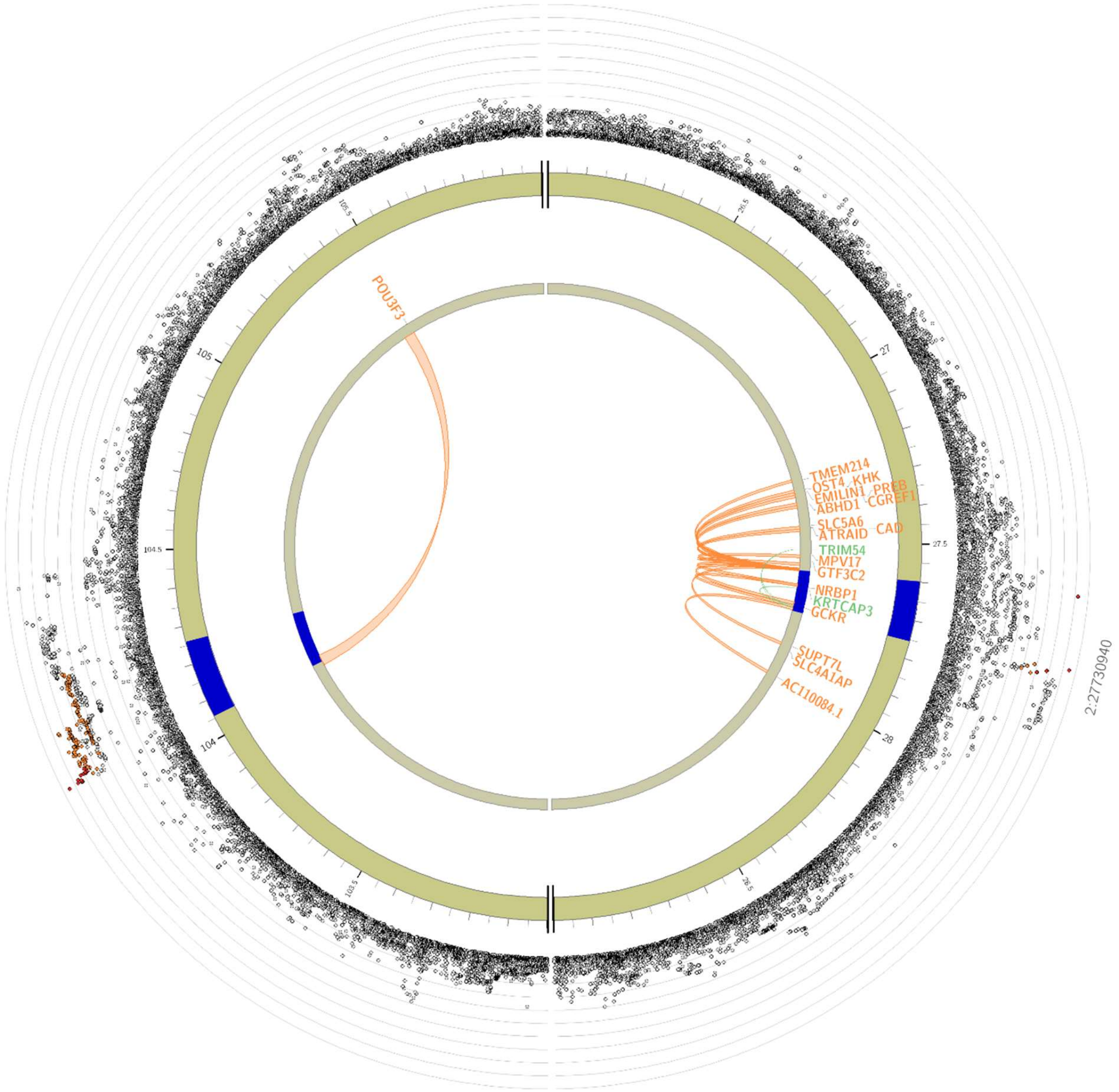

© 2022 Deak JD et al. *JAMA Network Open.*

(c) Chromosome 4

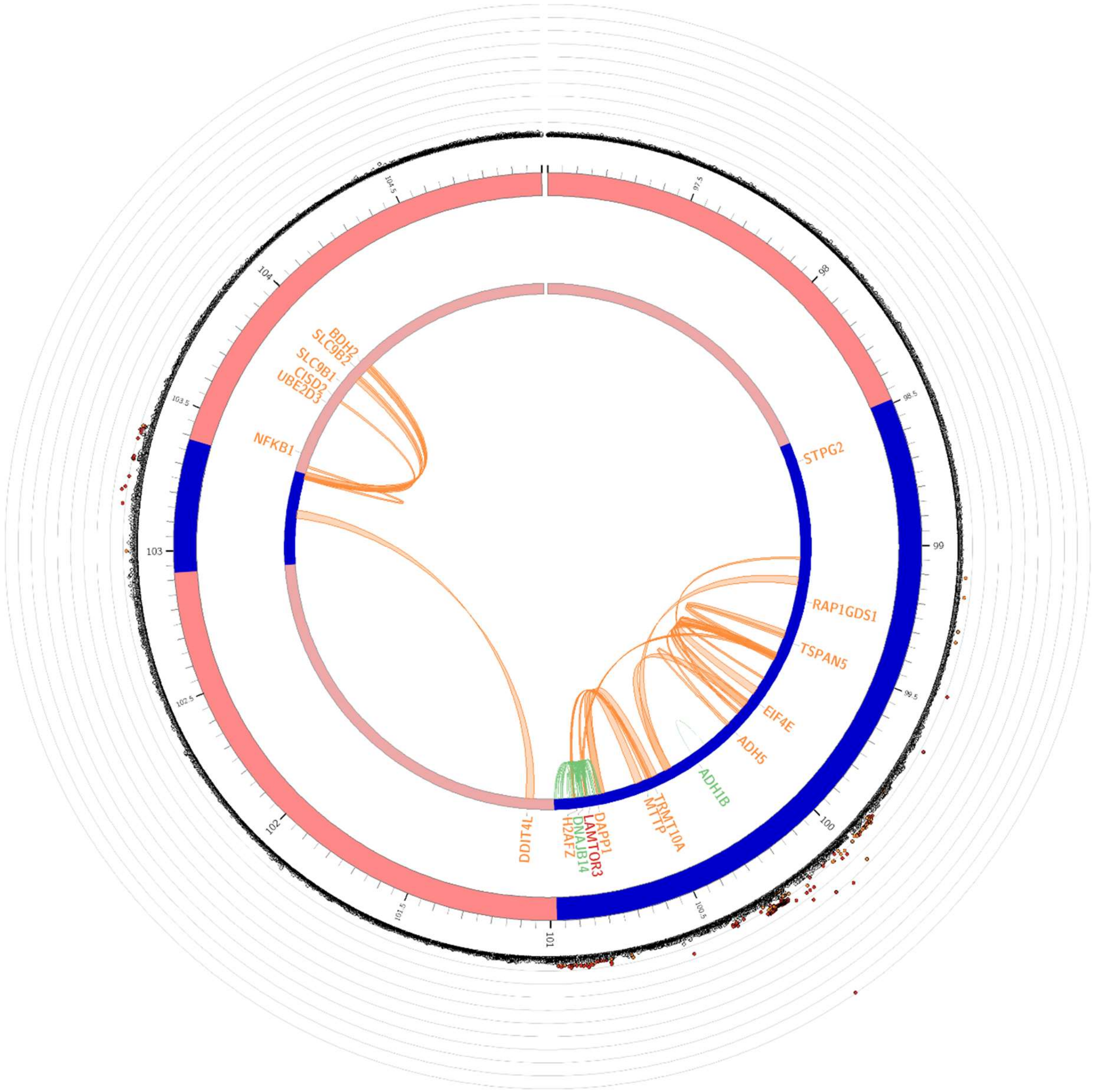

© 2022 Deak JD et al. *JAMA Network Open.*

(d) Chromosome 7

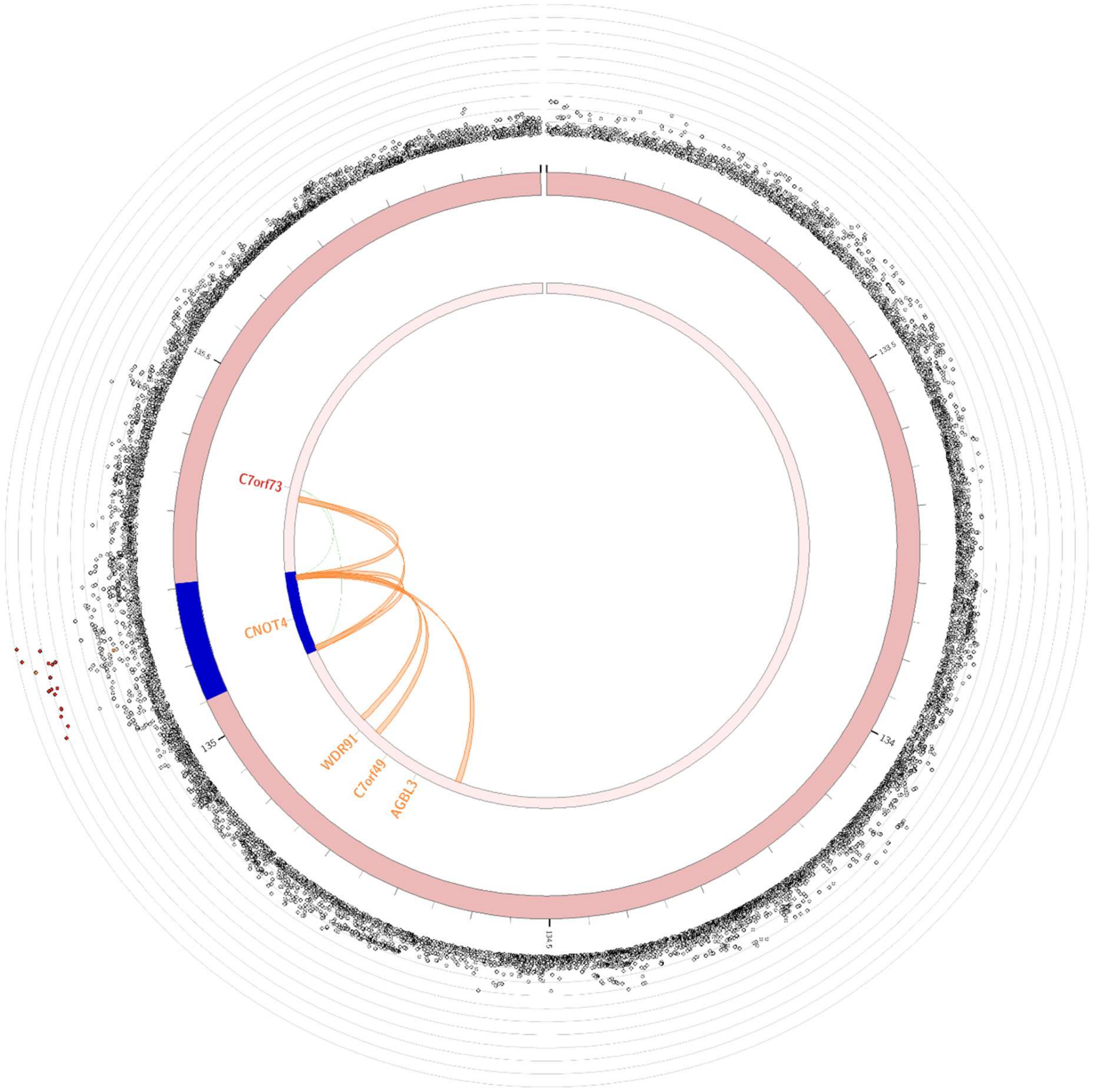

© 2022 Deak JD et al. *JAMA Network Open.*

(e) Chromosome 9

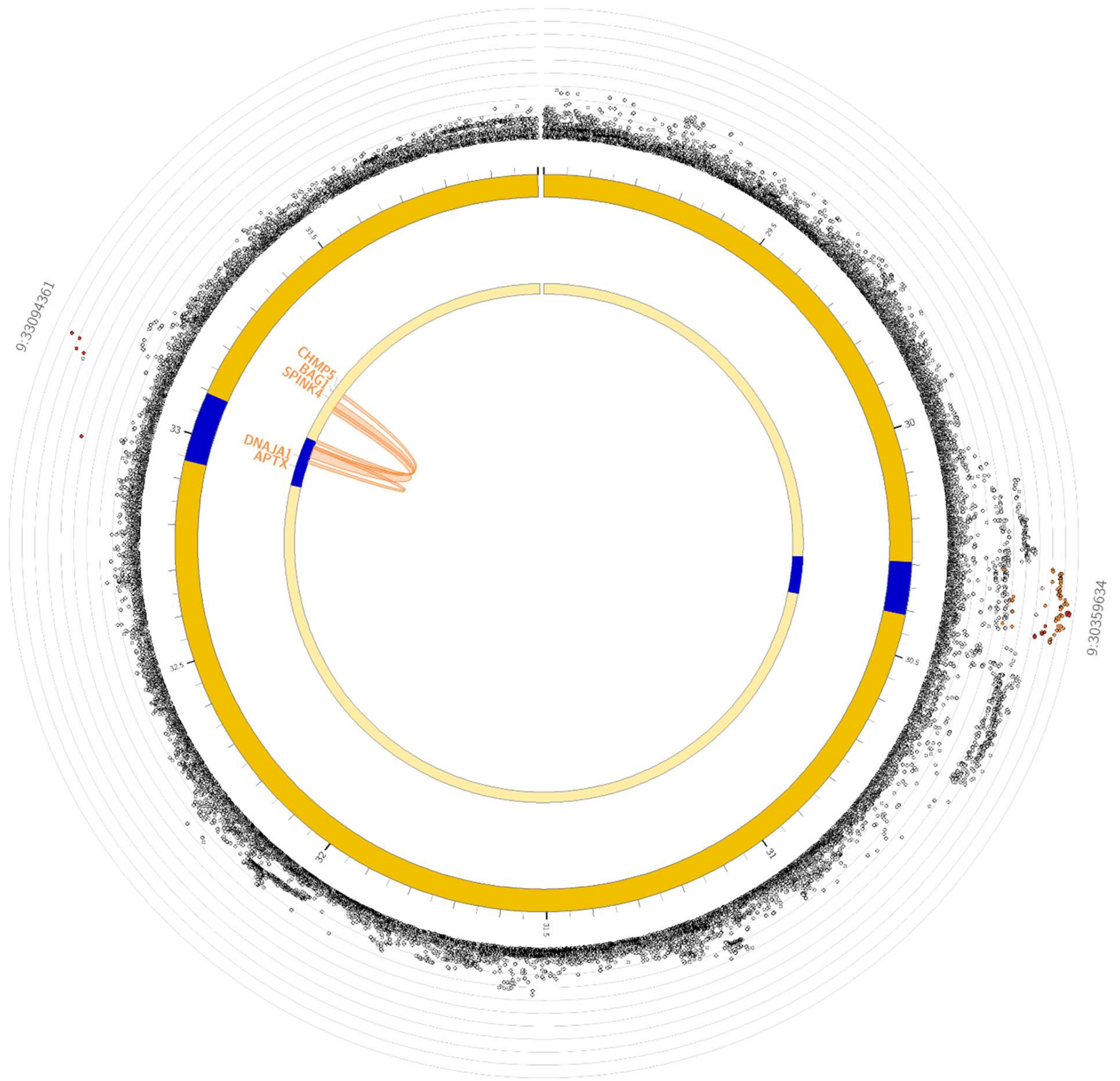

© 2022 Deak JD et al. *JAMA Network Open.*

(f) Chromosome 10

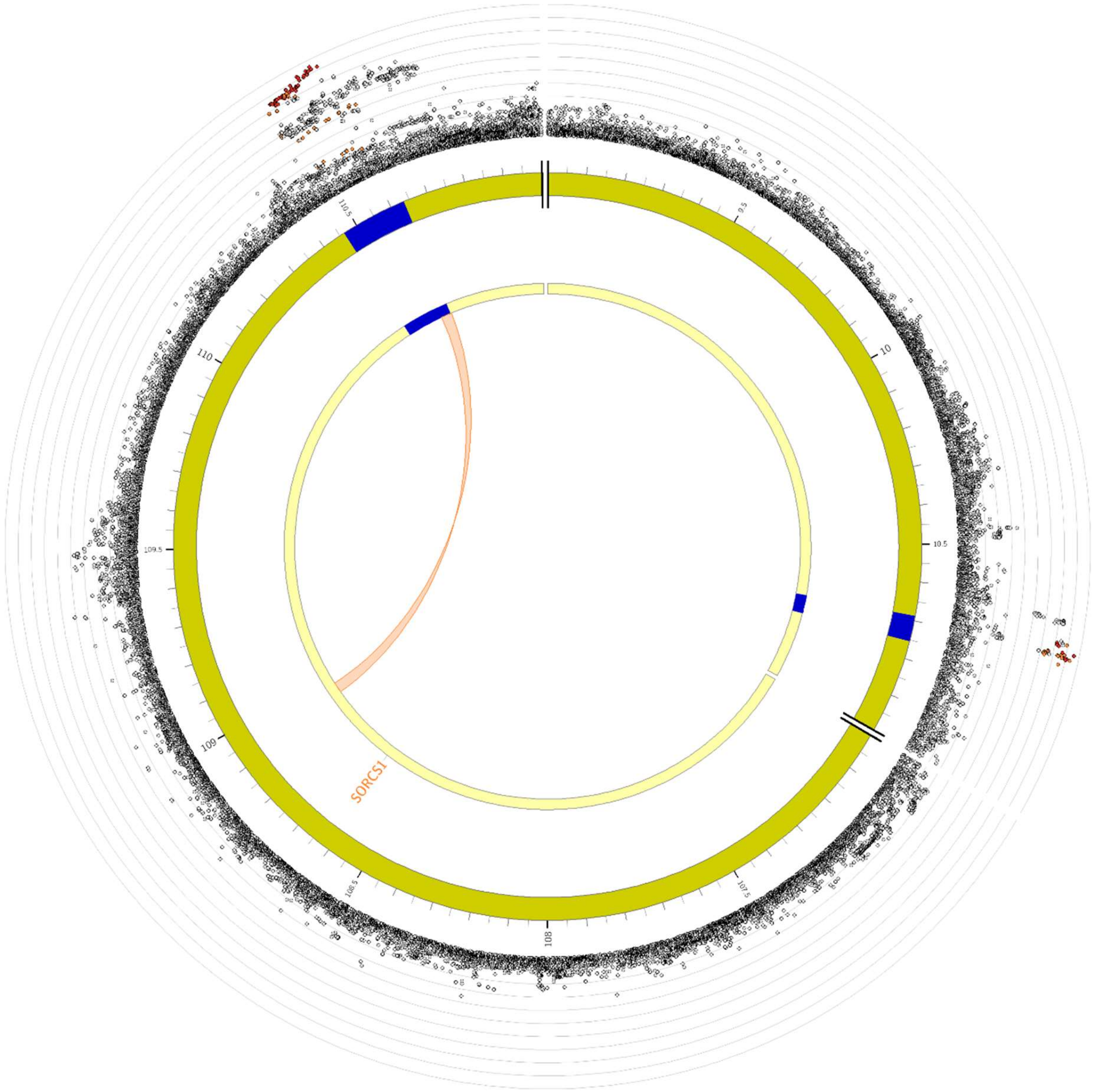

© 2022 Deak JD et al. *JAMA Network Open.*

(g) Chromosome 11

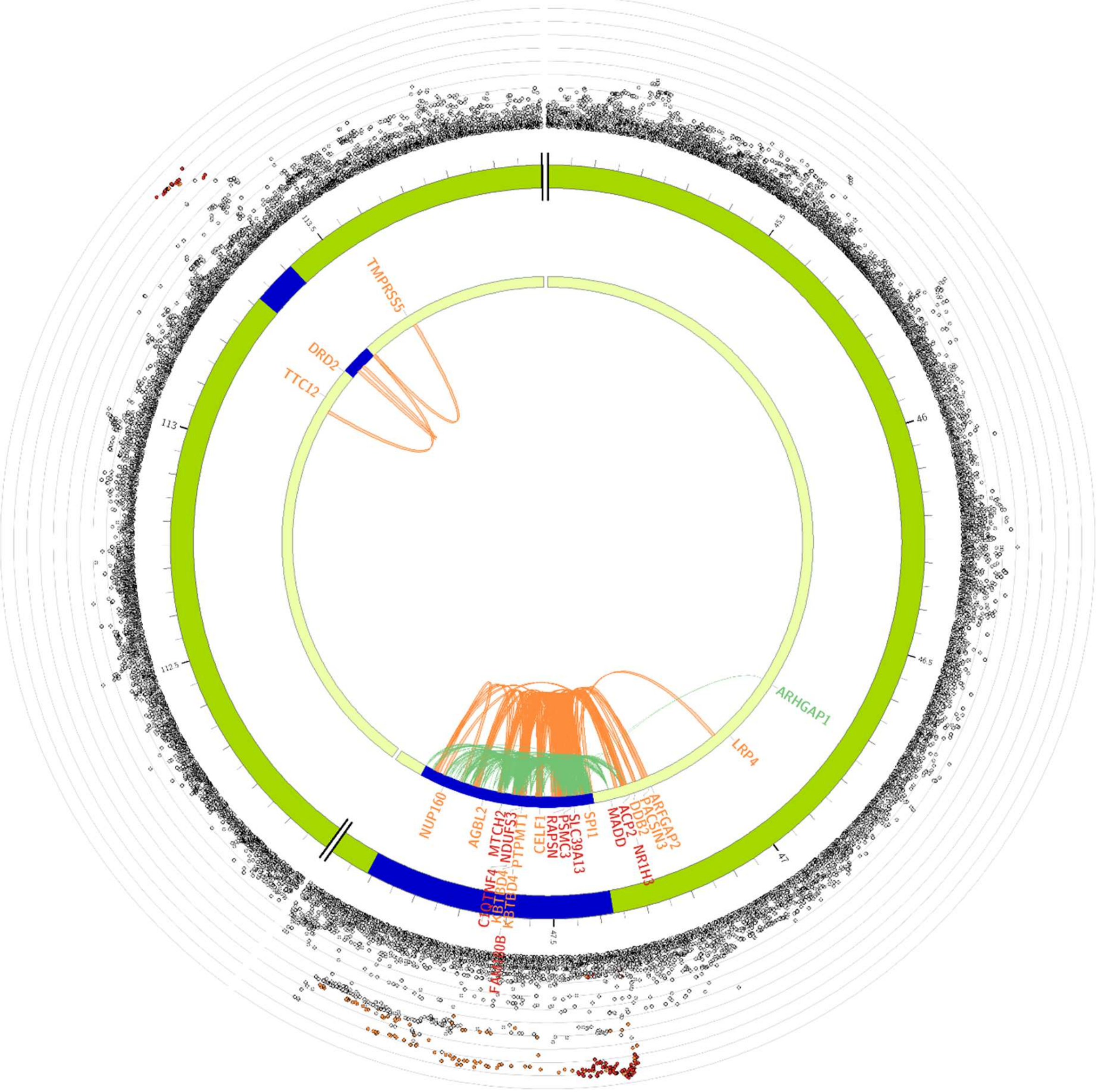

© 2022 Deak JD et al. *JAMA Network Open.*

(h) Chromosome 17

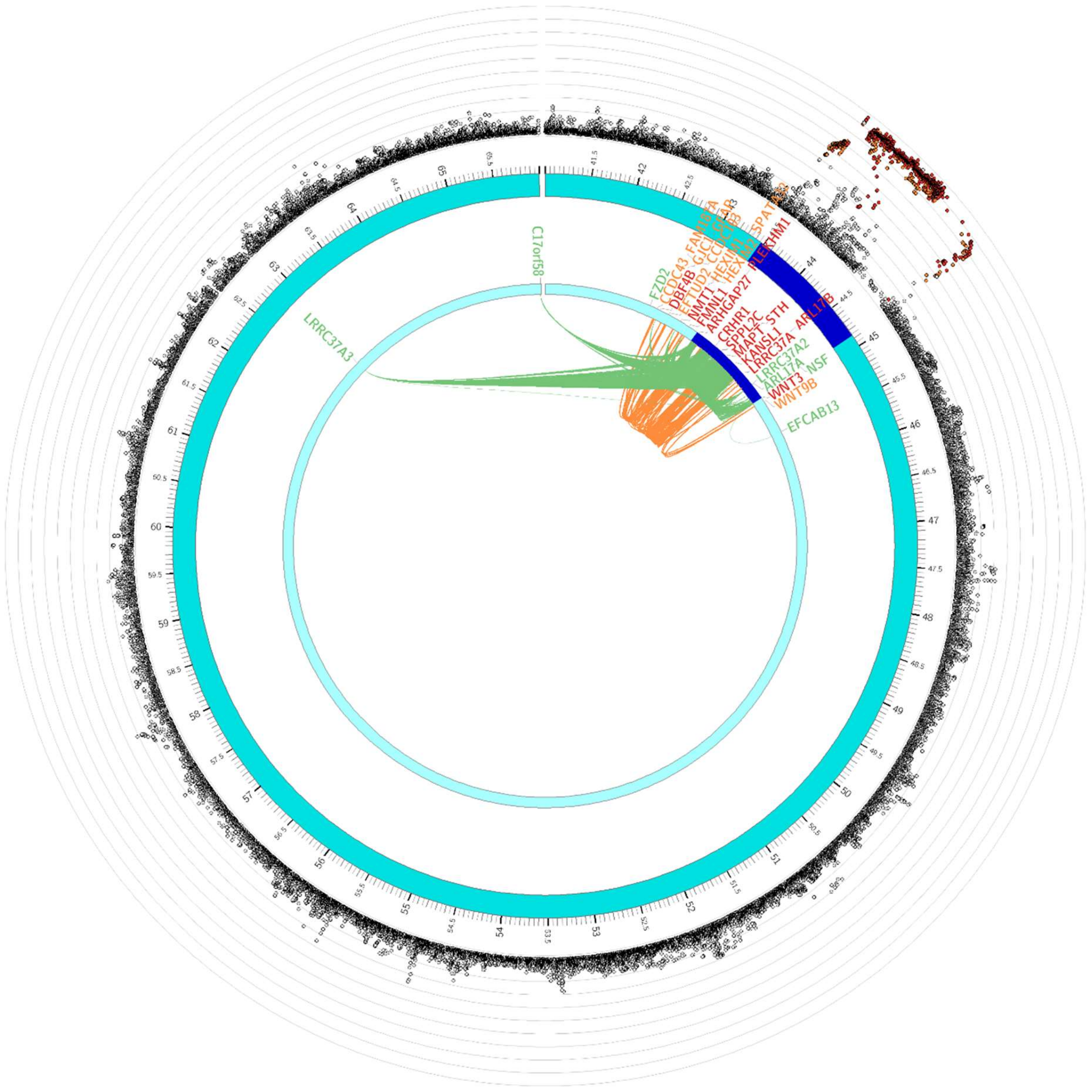

© 2022 Deak JD et al. *JAMA Network Open.*

(i) Chromosome 19

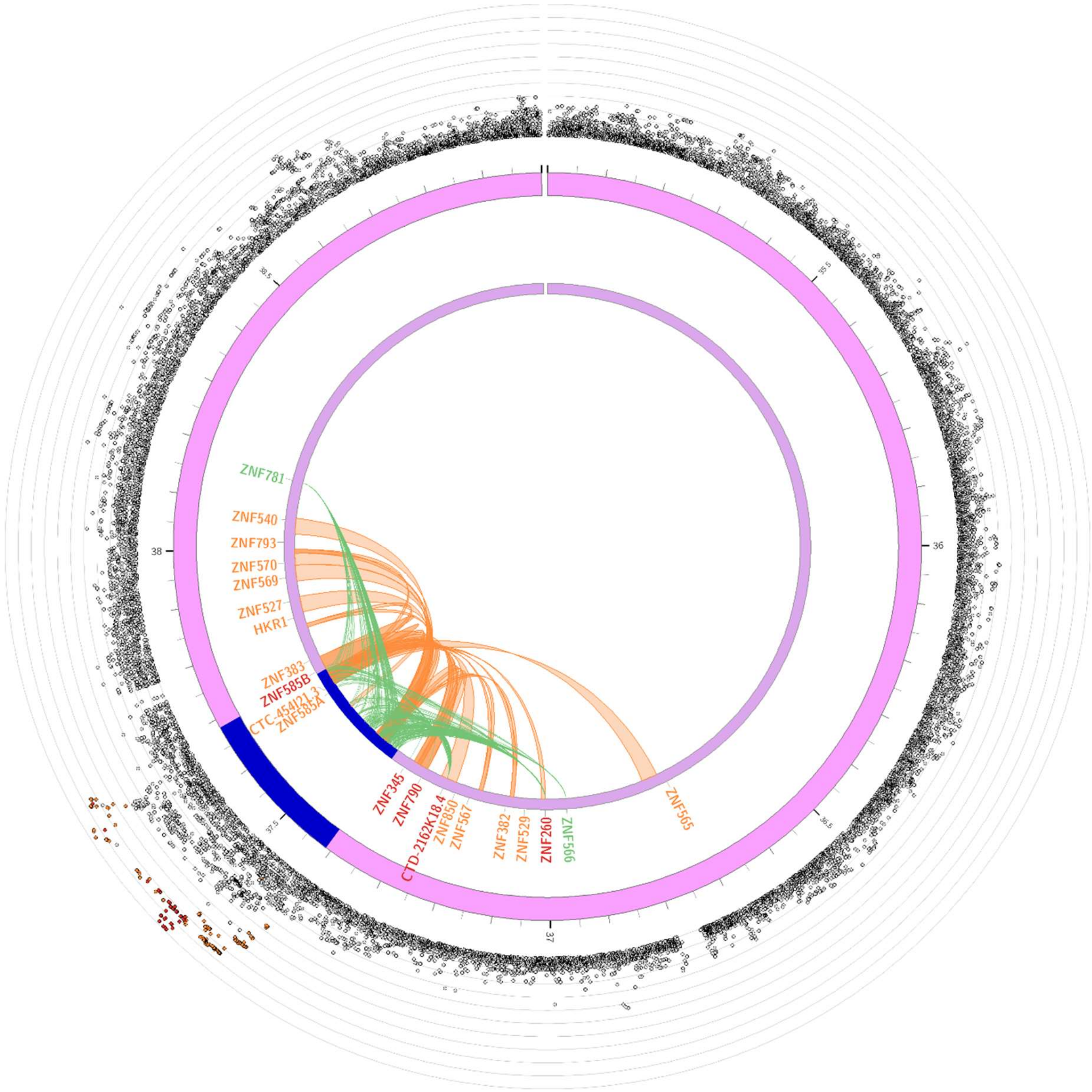

© 2022 Deak JD et al. *JAMA Network Open.*

eFigure 16. Circos Plots for Chromosomes Containing Genome-Wide Significant Loci for the MHAI MTAG GWAS

Note. **Outer most layer** is a Manhattan plot of genome-wide association study (GWAS) single-nucleotide polymorphisms (SNPs) with $p \leq 0.05$. SNPs are plotted by chromosomal position along the *x*-axis with their corresponding $-\log^{-10}$ *p*-value on the *y*-axis. Linkage-disequilibrium (LD) between the identified lead SNP and surrounding SNPs is indicated from $r^2>0.8$ (red), $r^2>0.6$ (orange), $r^2>0.4$ (green), $r^2>0.2$ (blue). SNPs that are not in LD with the lead SNP ($r^2 \leq 0.02$) are gray. **Second layer (chromosome ring):** Chromosomal regions containing identified genomic risk loci are colored in blue. The names of genes implicated based upon variant associations with brain tissue expression quantitative trait loci (eQTLs) are colored green. The names of genes implicated based upon 3D chromatin interactions (Hi-C) are colored orange. Genes that are mapped based upon both eQTLs and Hi-C associations are colored red. **Third layer (chromosome ring):** Variants mapped to genes based upon associations with brain tissue eQTLs are linked in green. Variants mapped to genes based upon Hi-C data are linked in orange.

© 2022 Deak JD et al. *JAMA Network Open.*

(a) Chromosome 1

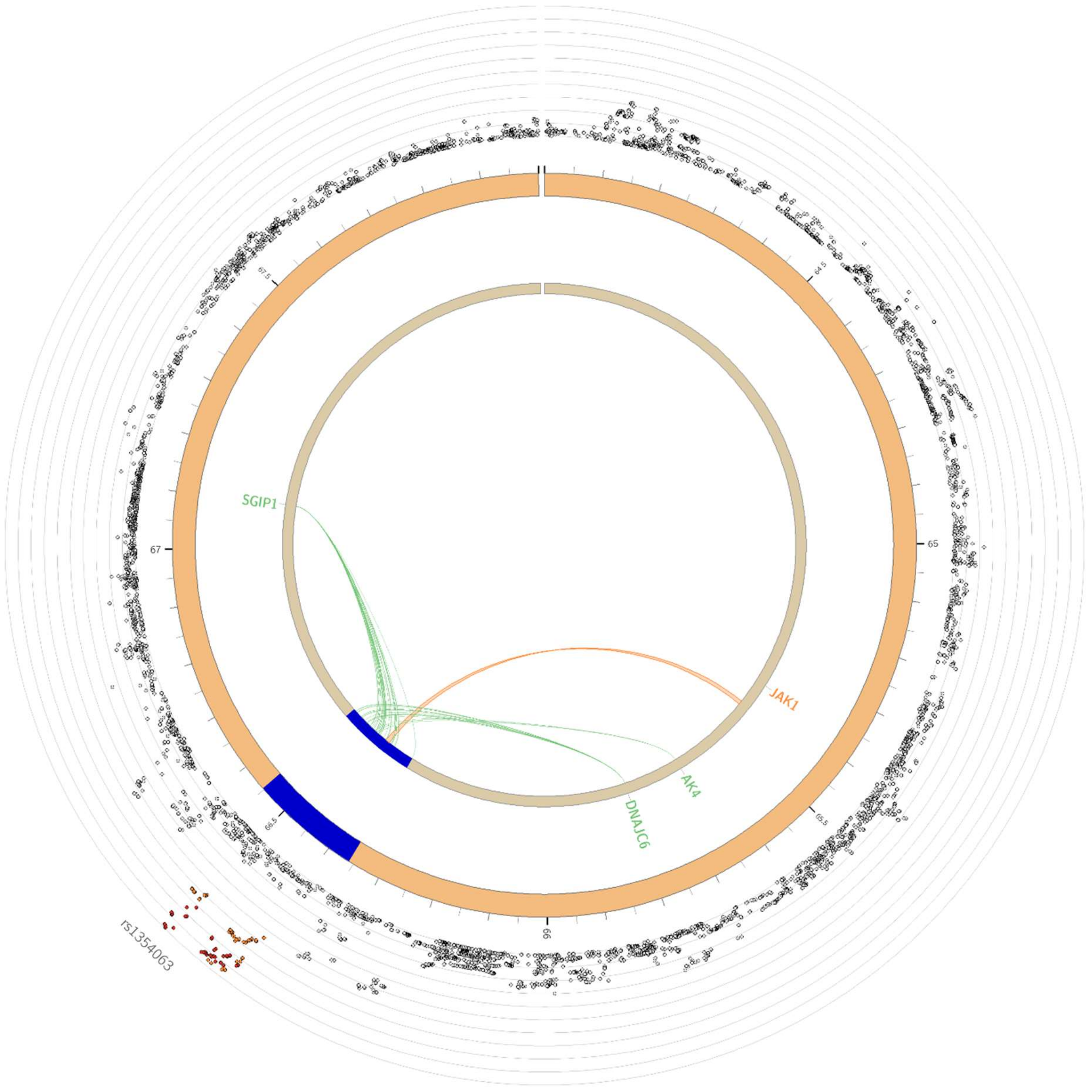

© 2022 Deak JD et al. *JAMA Network Open.*

(b) Chromosome 2

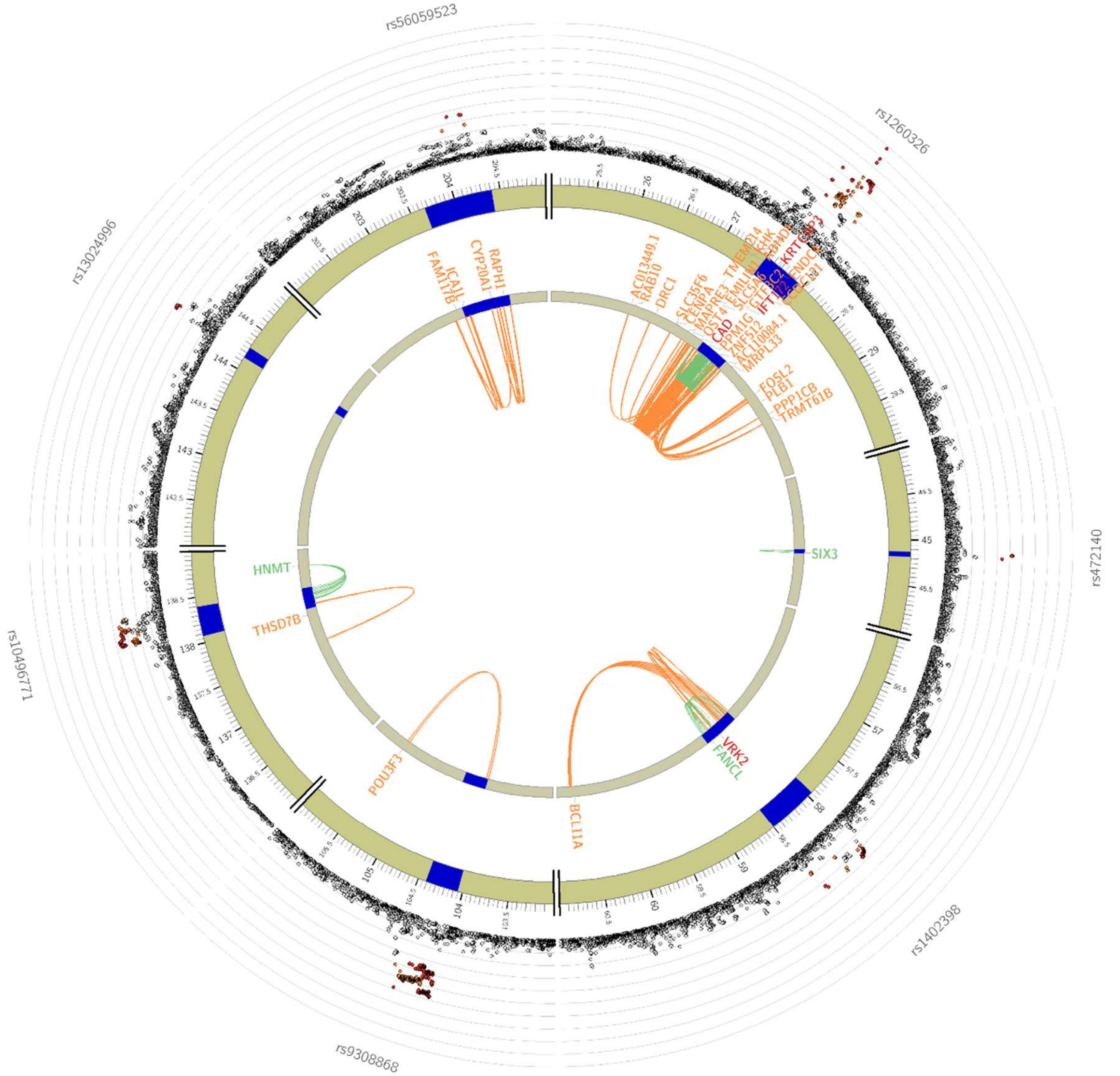

© 2022 Deak JD et al. *JAMA Network Open.*

(c) Chromosome 3

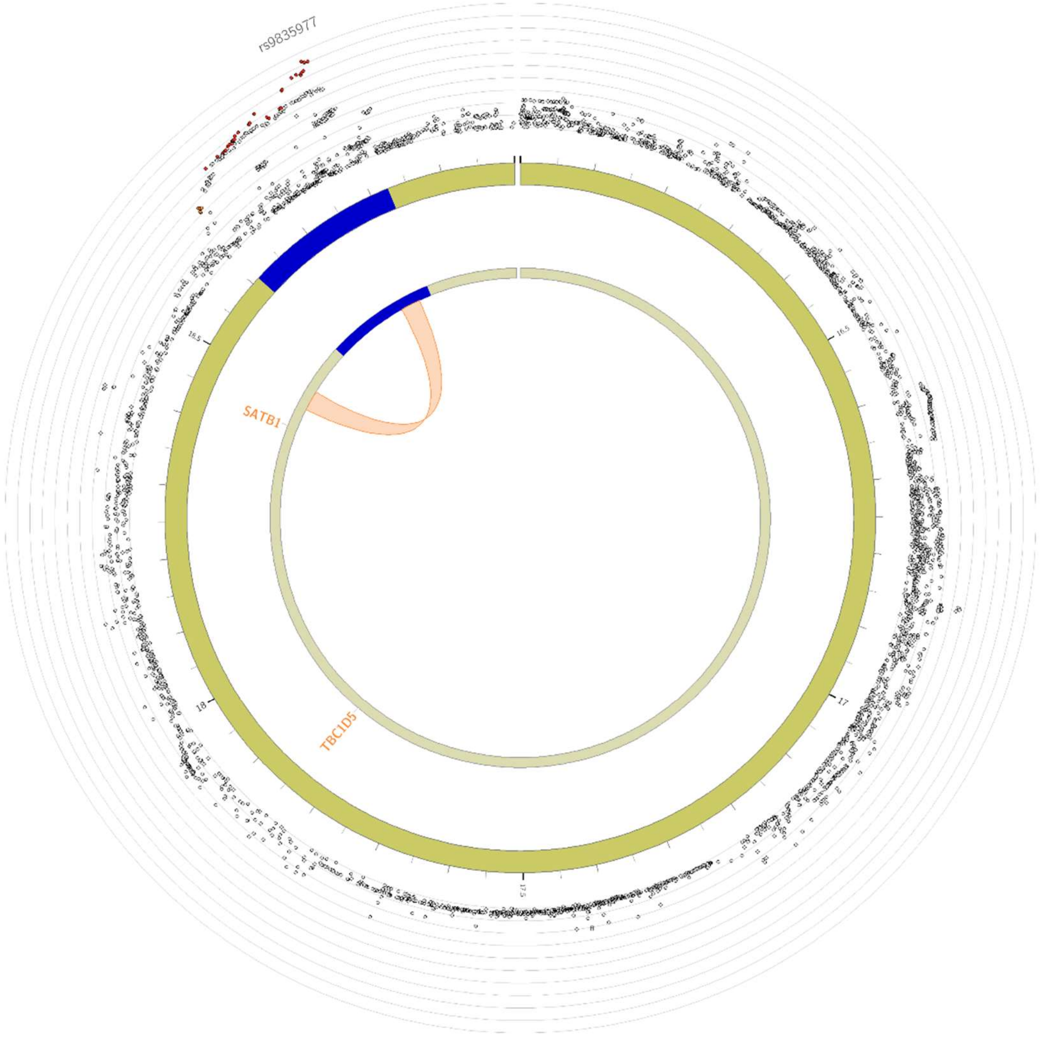

© 2022 Deak JD et al. *JAMA Network Open.*

(d) Chromosome 4

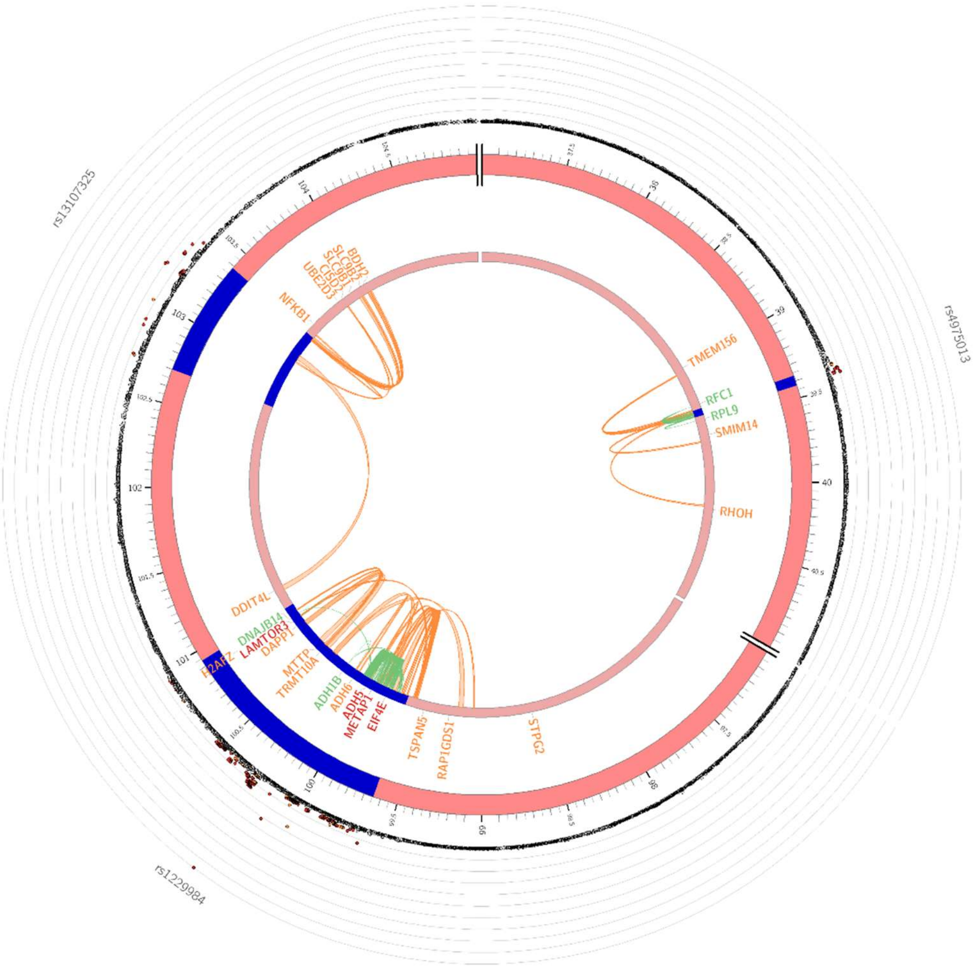

© 2022 Deak JD et al. *JAMA Network Open.*

(e) Chromosome 5

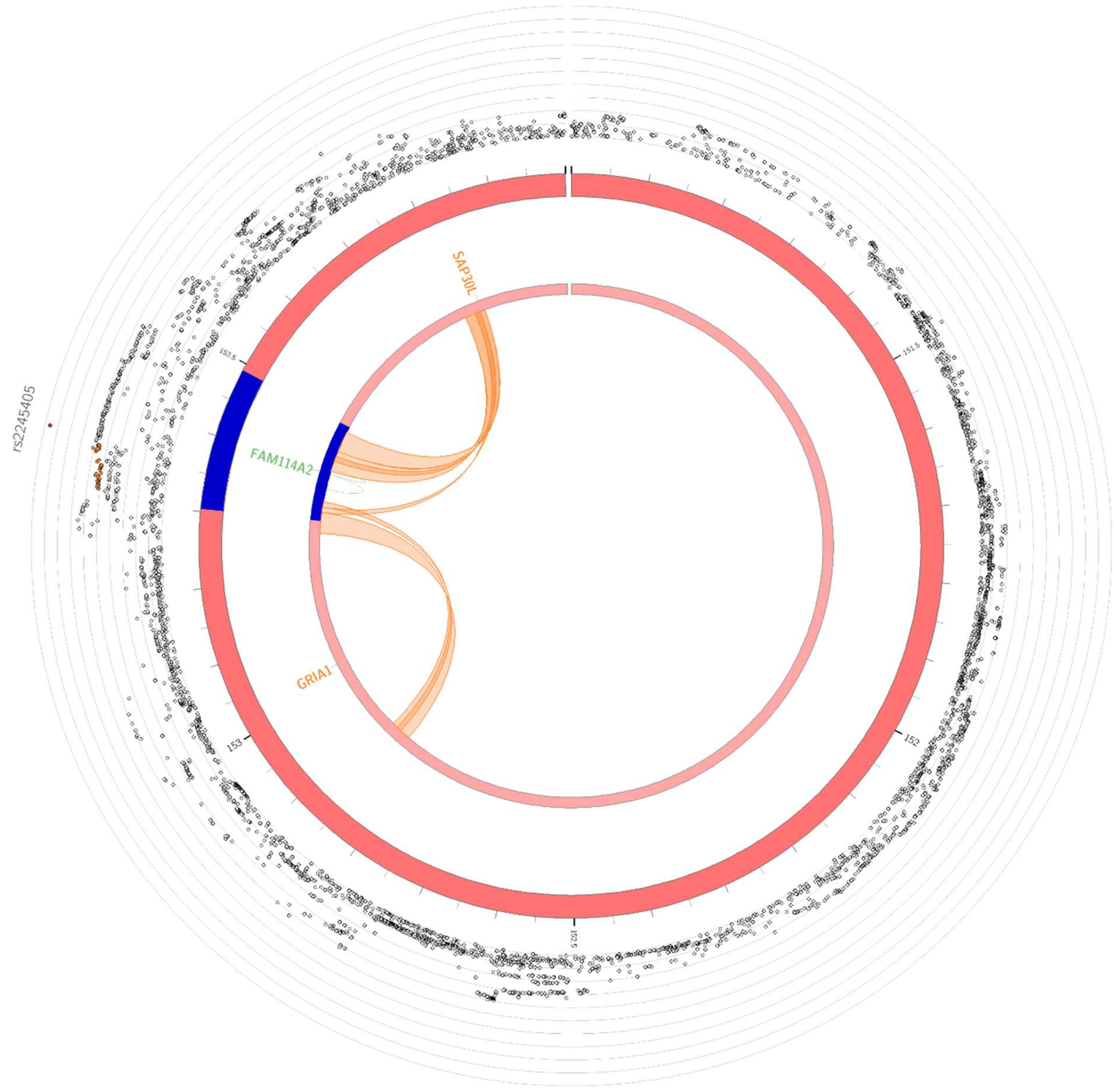

© 2022 Deak JD et al. *JAMA Network Open.*

(f) Chromosome 7

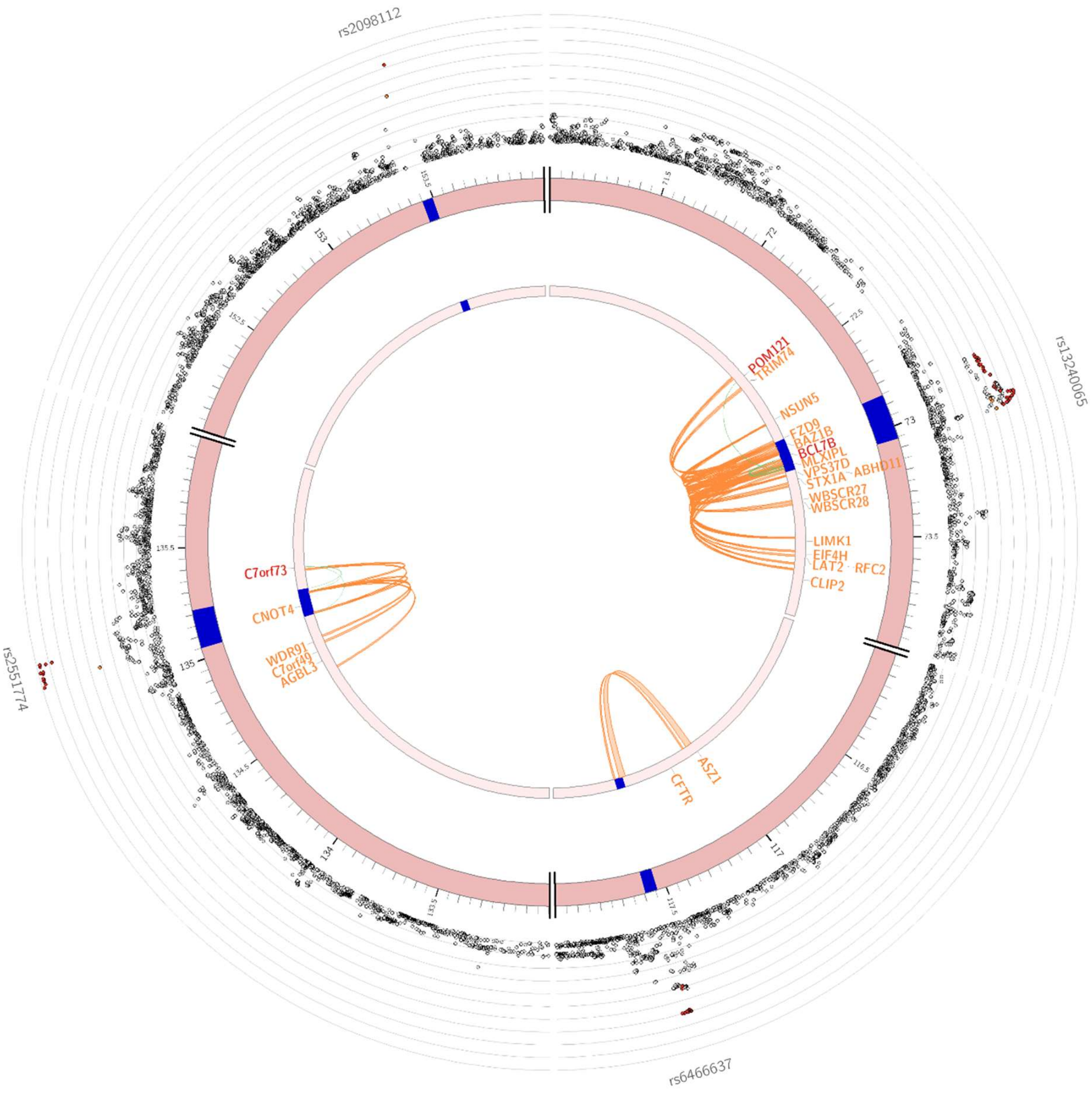

© 2022 Deak JD et al. *JAMA Network Open.*

(g) Chromosome 8

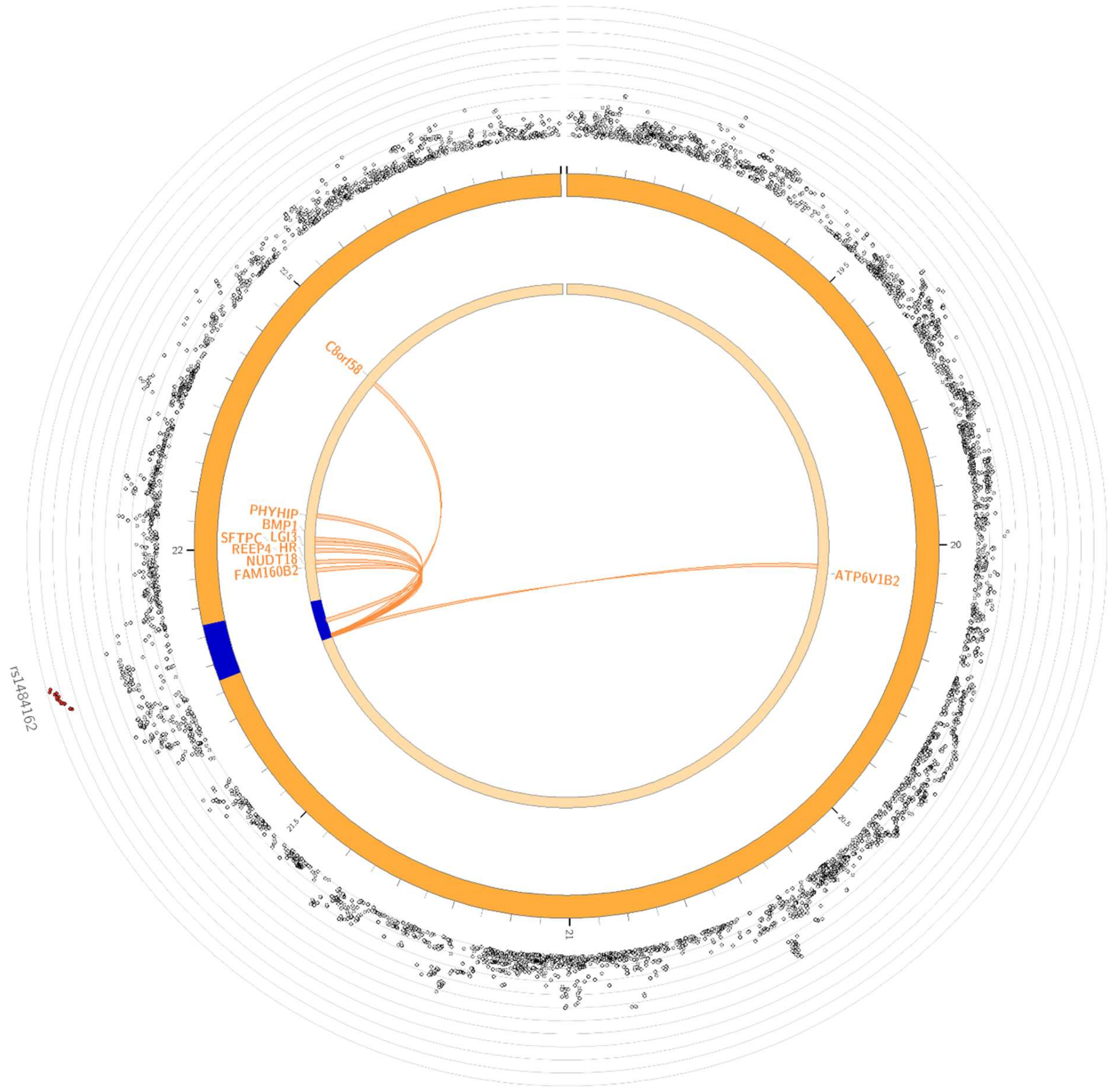

© 2022 Deak JD et al. *JAMA Network Open.*

(h) Chromosome 10

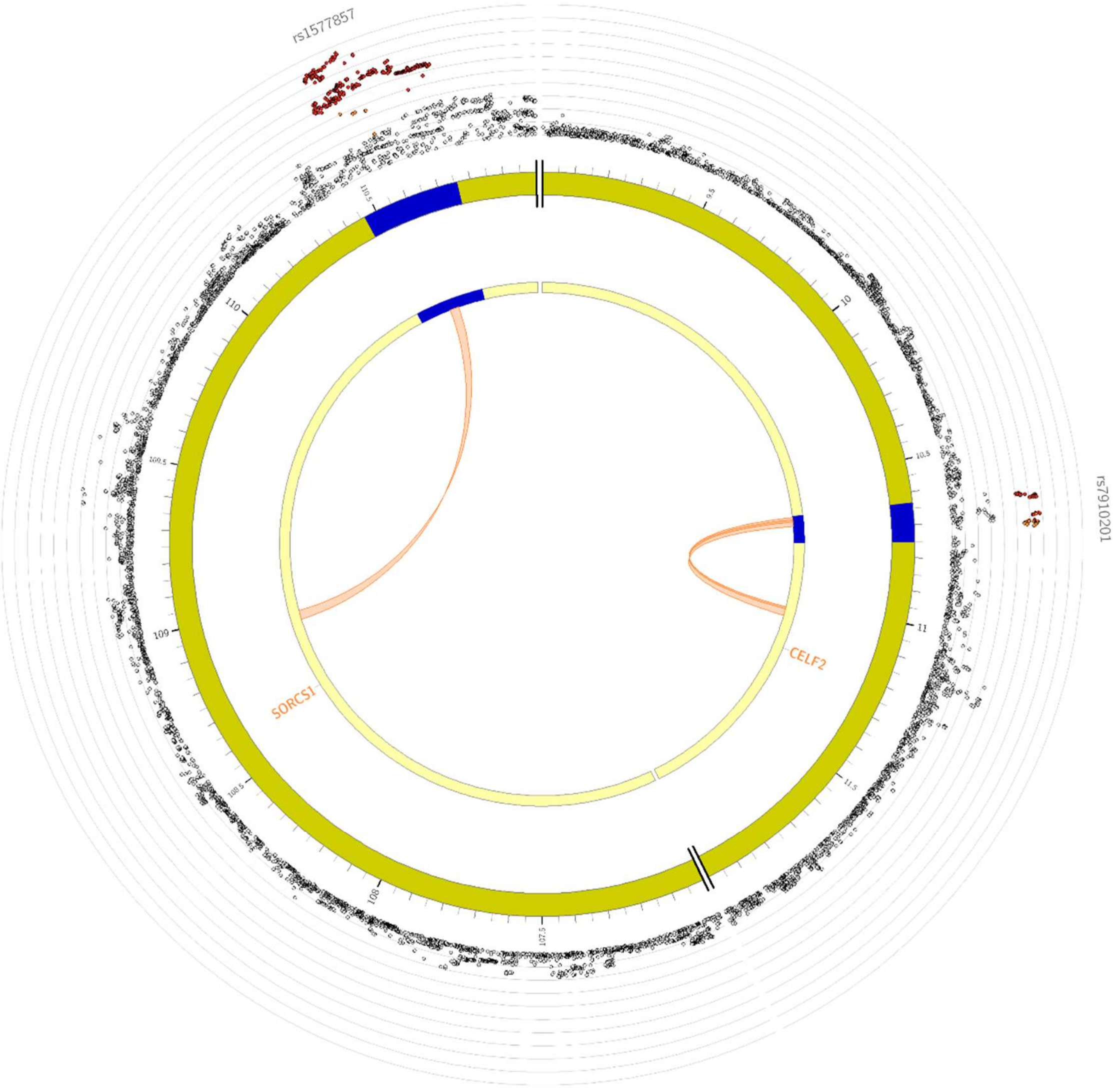

© 2022 Deak JD et al. *JAMA Network Open.*

(i) Chromosome 11

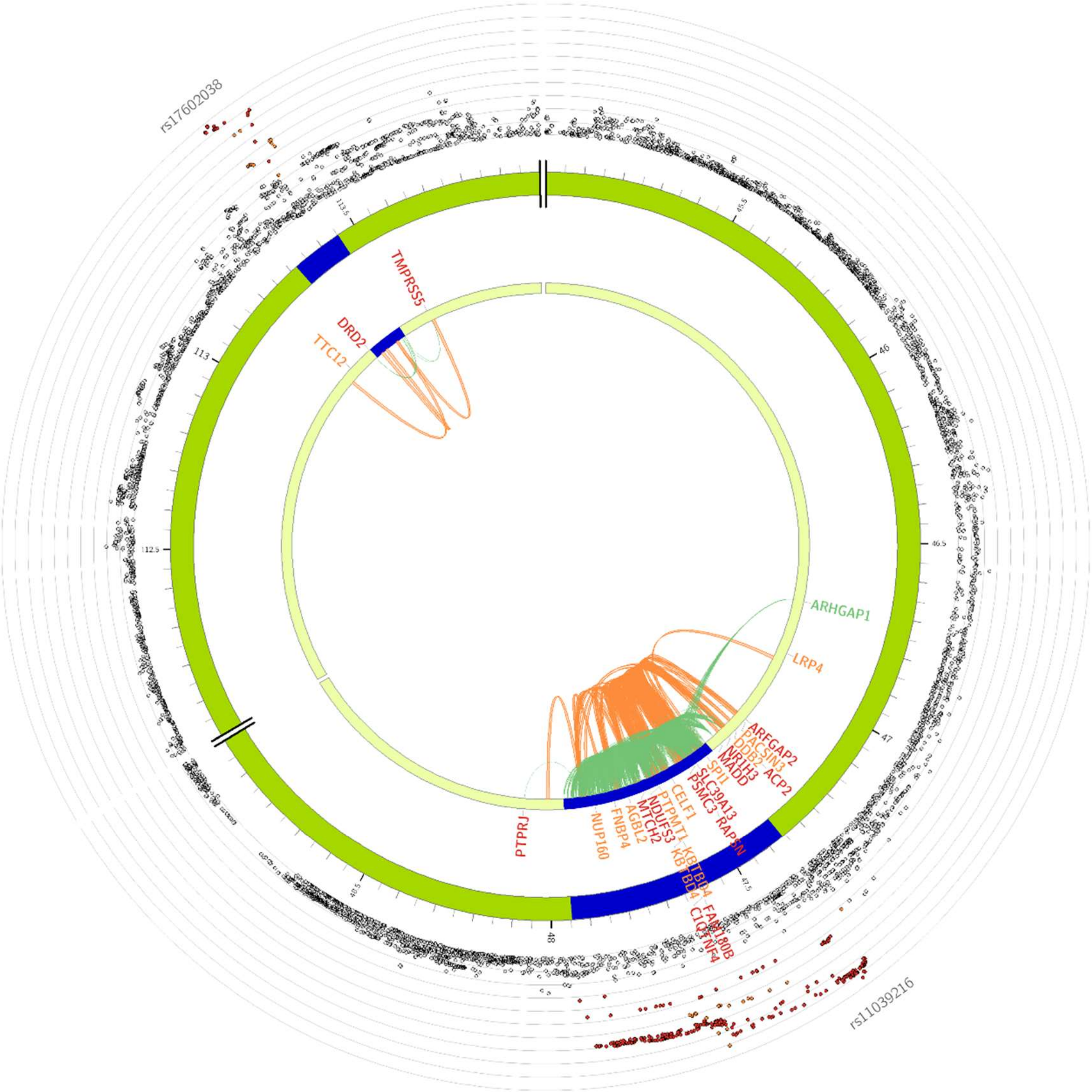

© 2022 Deak JD et al. *JAMA Network Open.*

(j) Chromosome 12

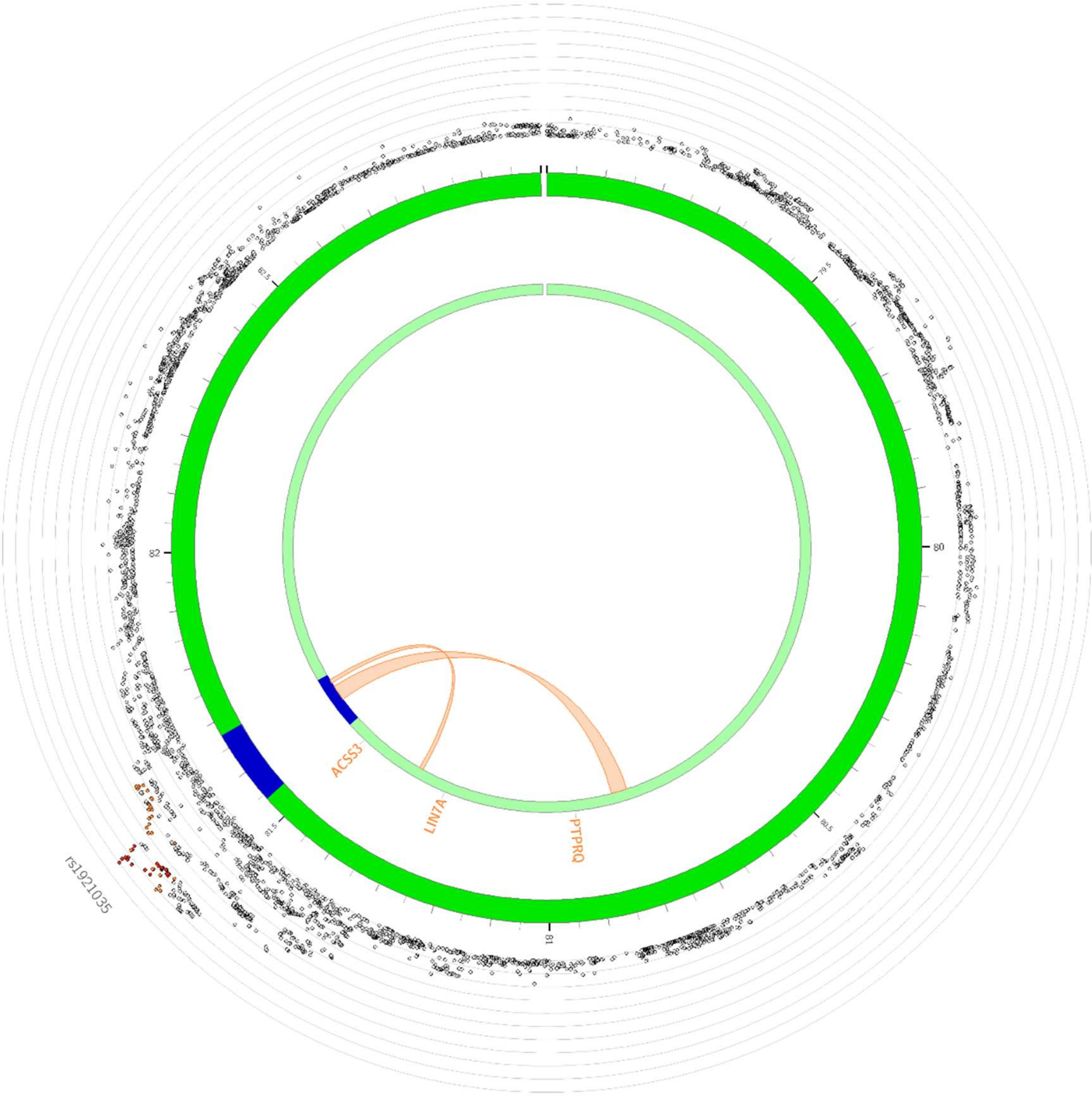

© 2022 Deak JD et al. *JAMA Network Open.*

(k) Chromosome 13

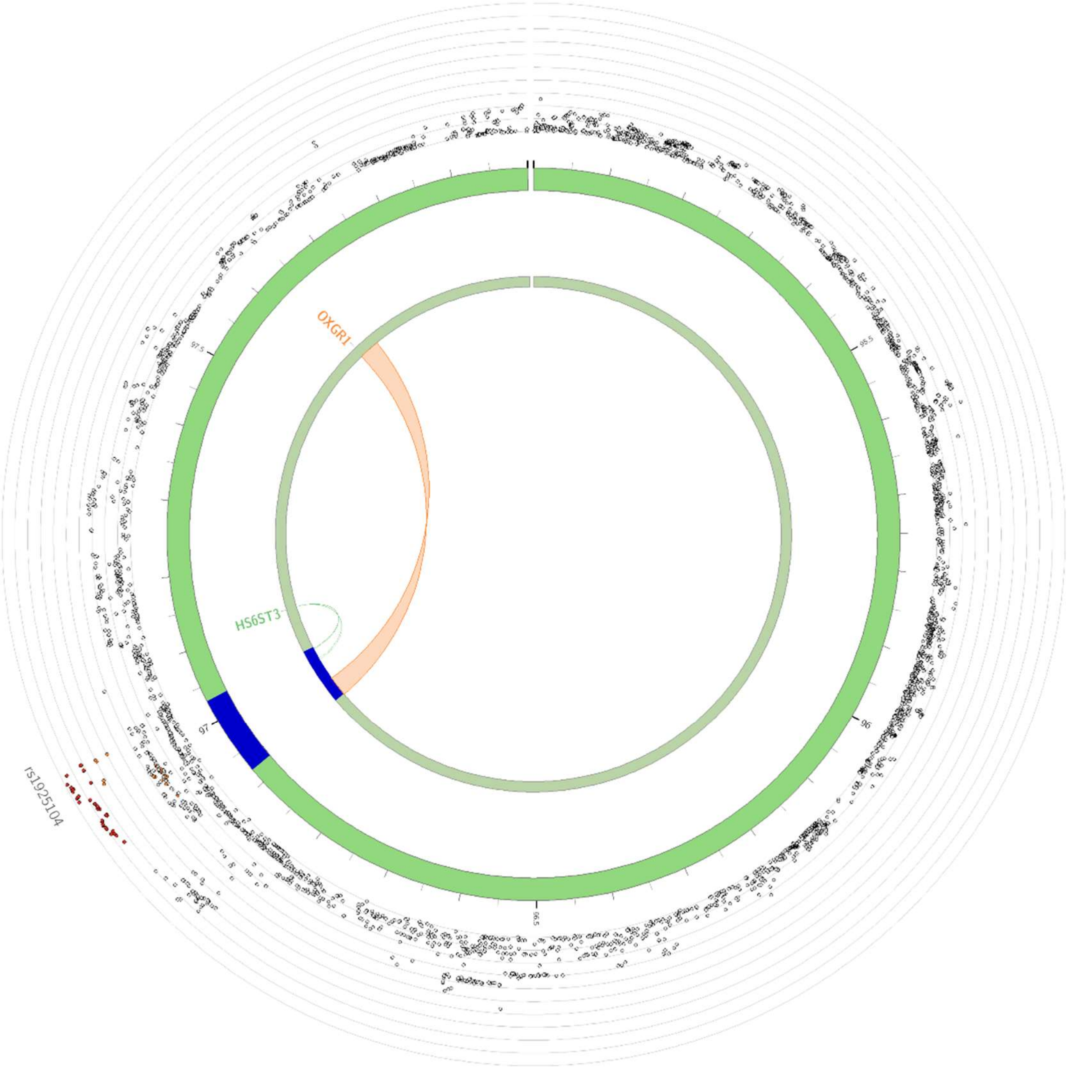

© 2022 Deak JD et al. *JAMA Network Open.*

(l) Chromosome 14

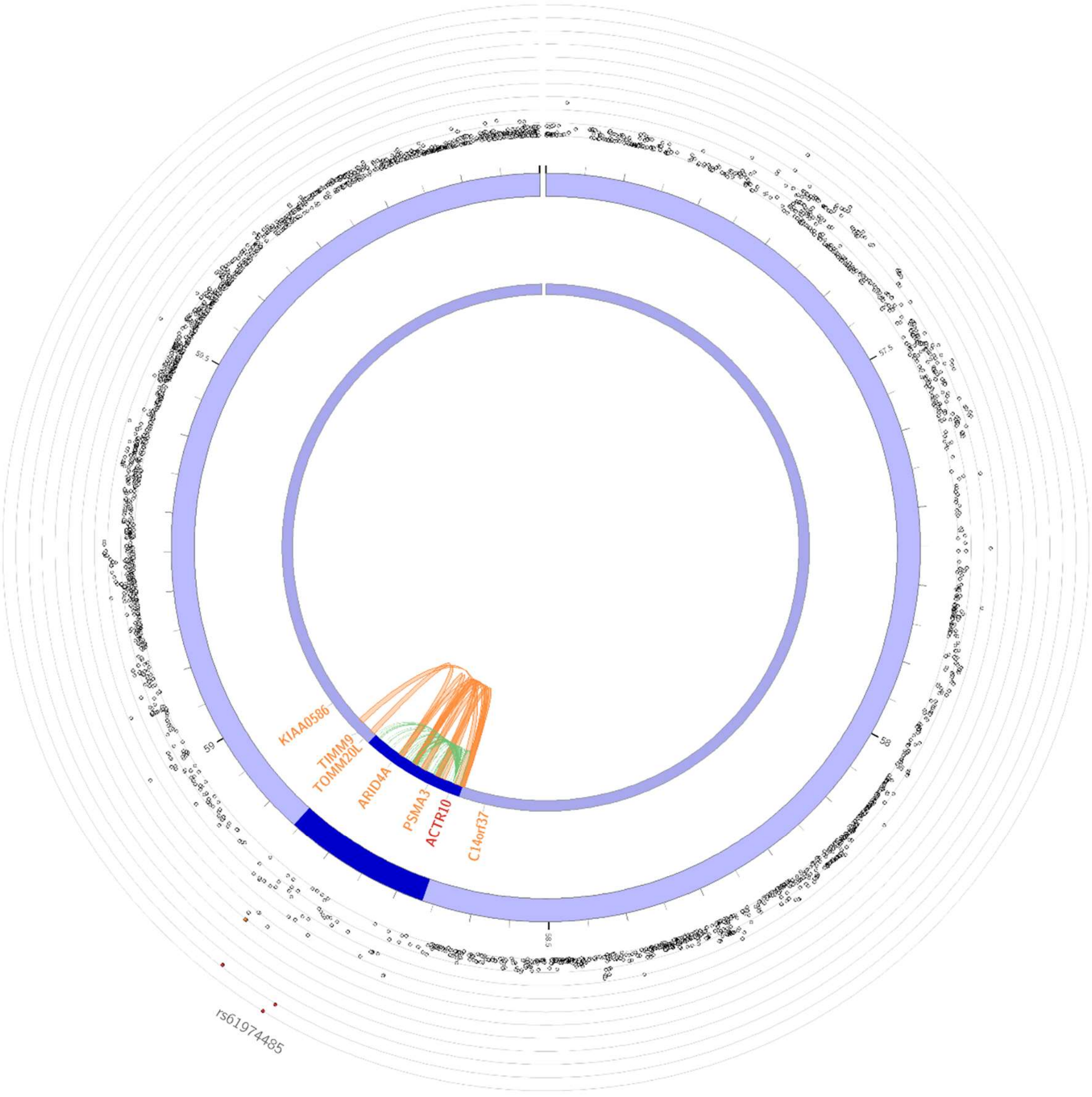

© 2022 Deak JD et al. *JAMA Network Open.*

(m) Chromosome 15

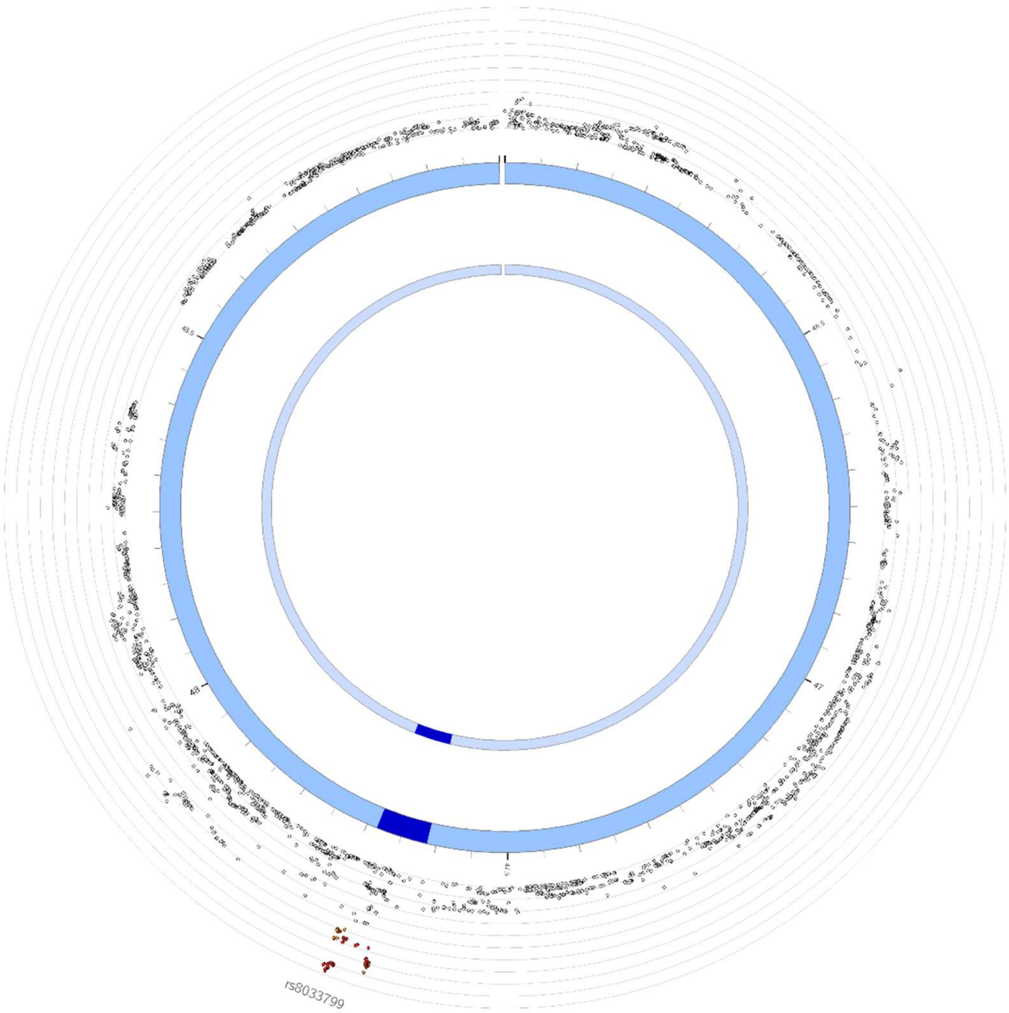

© 2022 Deak JD et al. *JAMA Network Open.*

(n) Chromosome 16

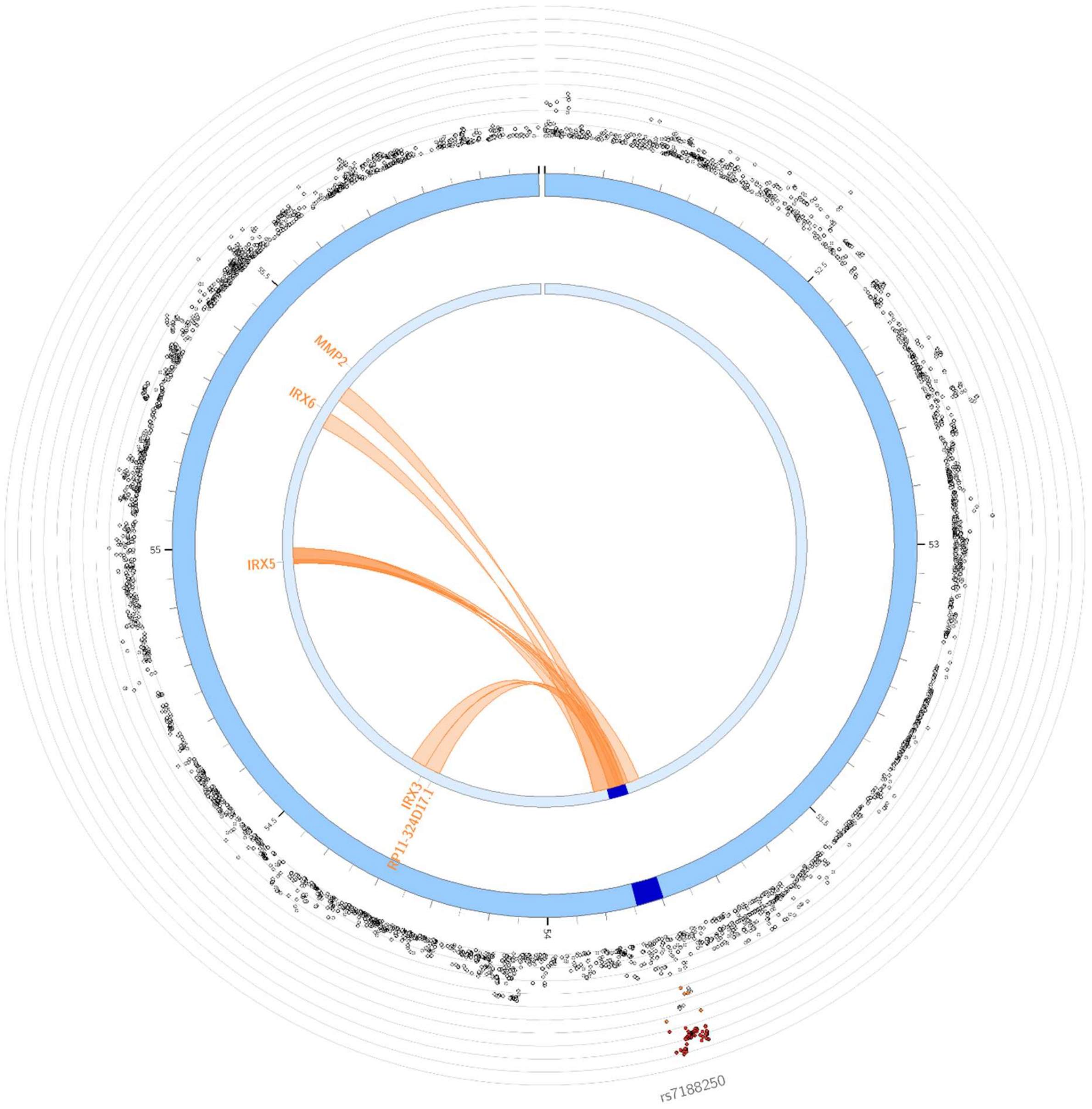

© 2022 Deak JD et al. *JAMA Network Open.*

(o) Chromosome 17

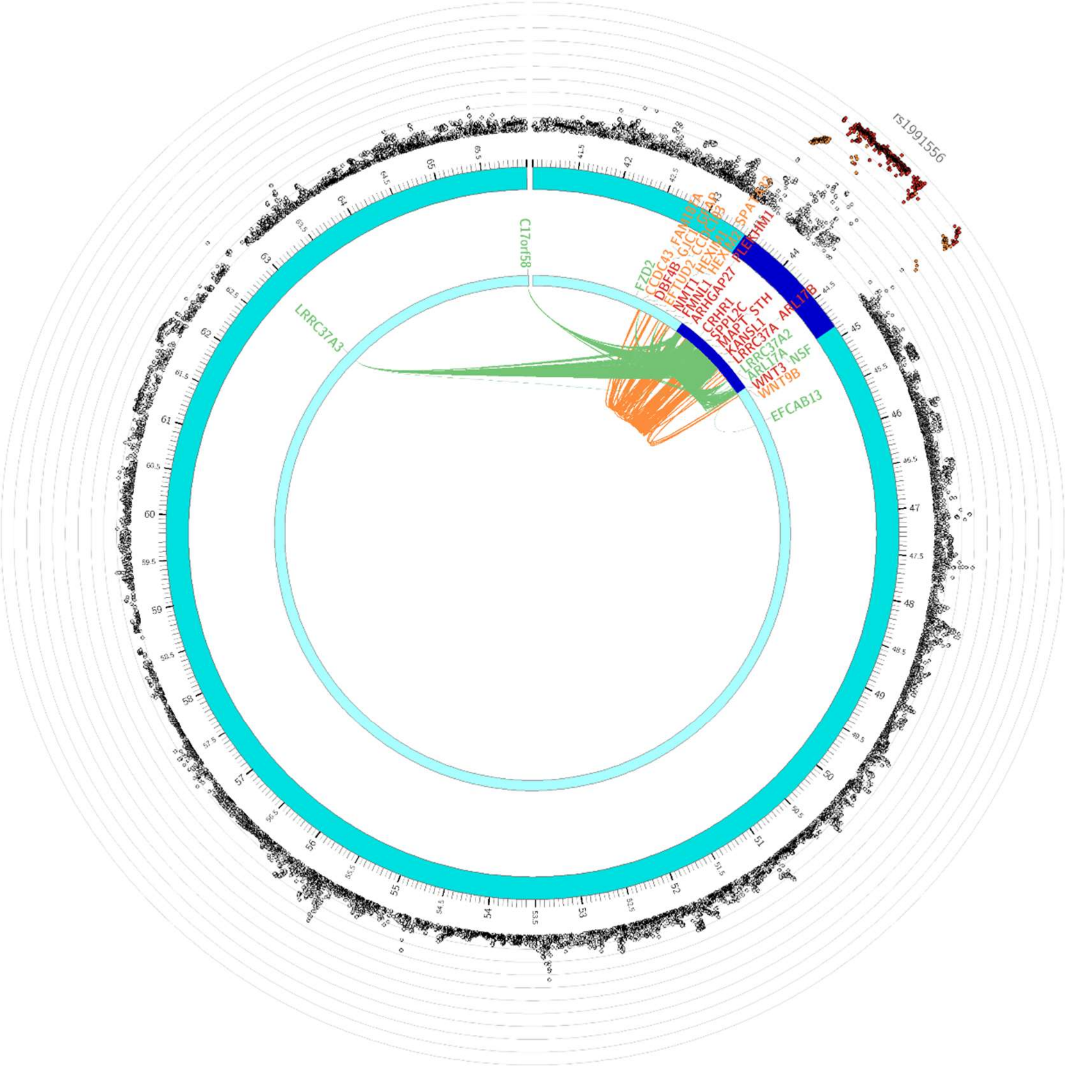

© 2022 Deak JD et al. *JAMA Network Open.*

(p) Chromosome 19

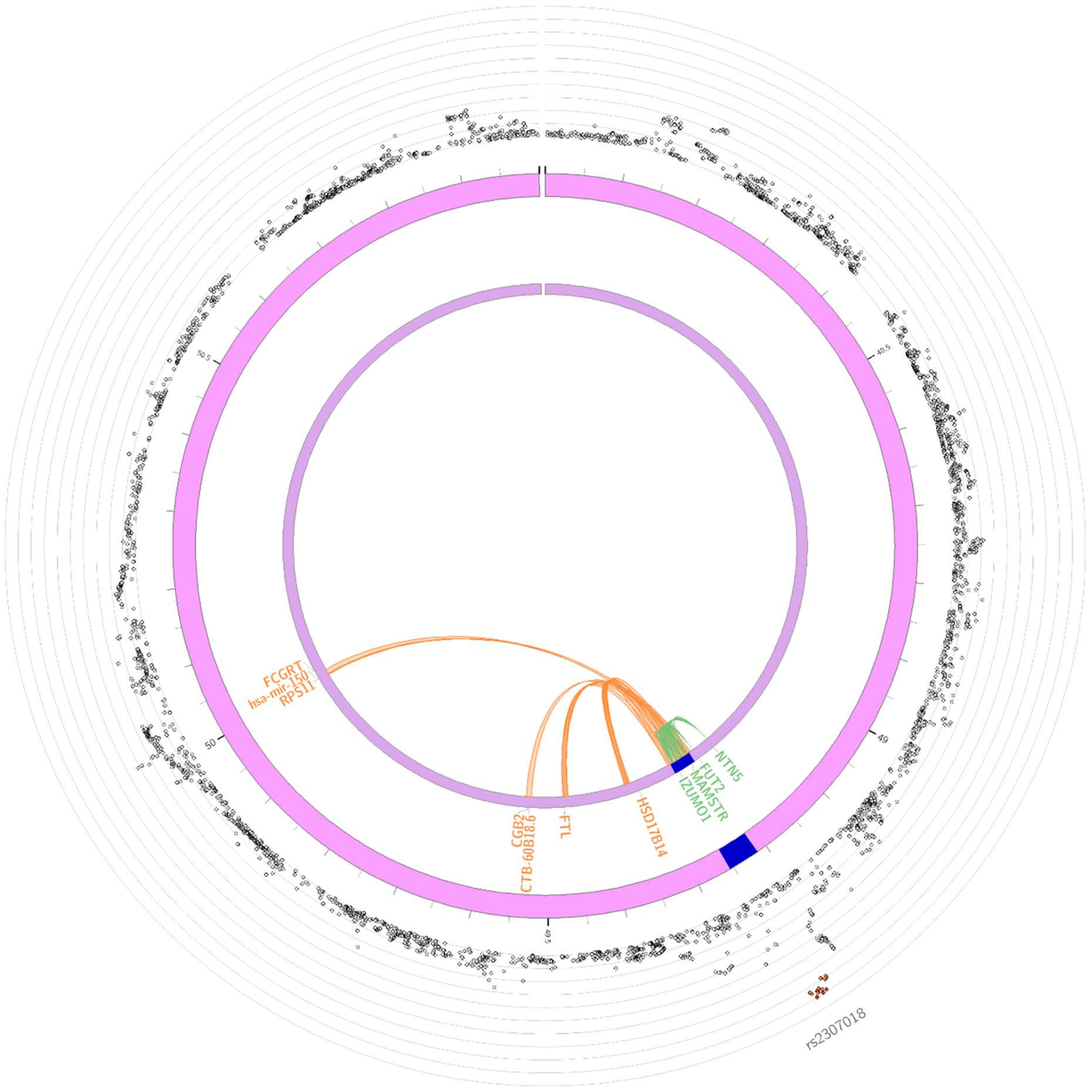

© 2022 Deak JD et al. *JAMA Network Open.*

(q) Chromosome 20

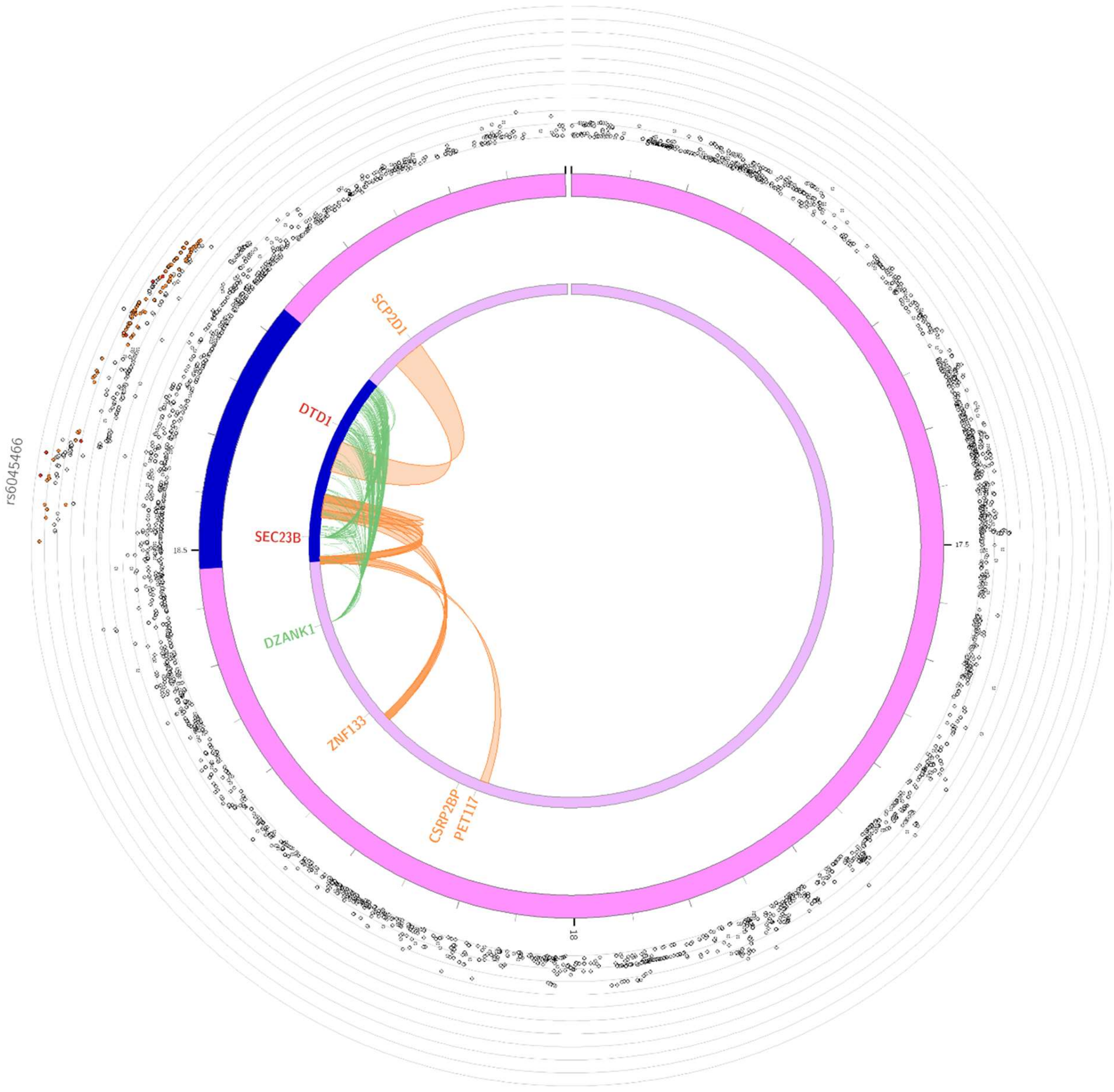

© 2022 Deak JD et al. *JAMA Network Open.*

(r) Chromosome 22

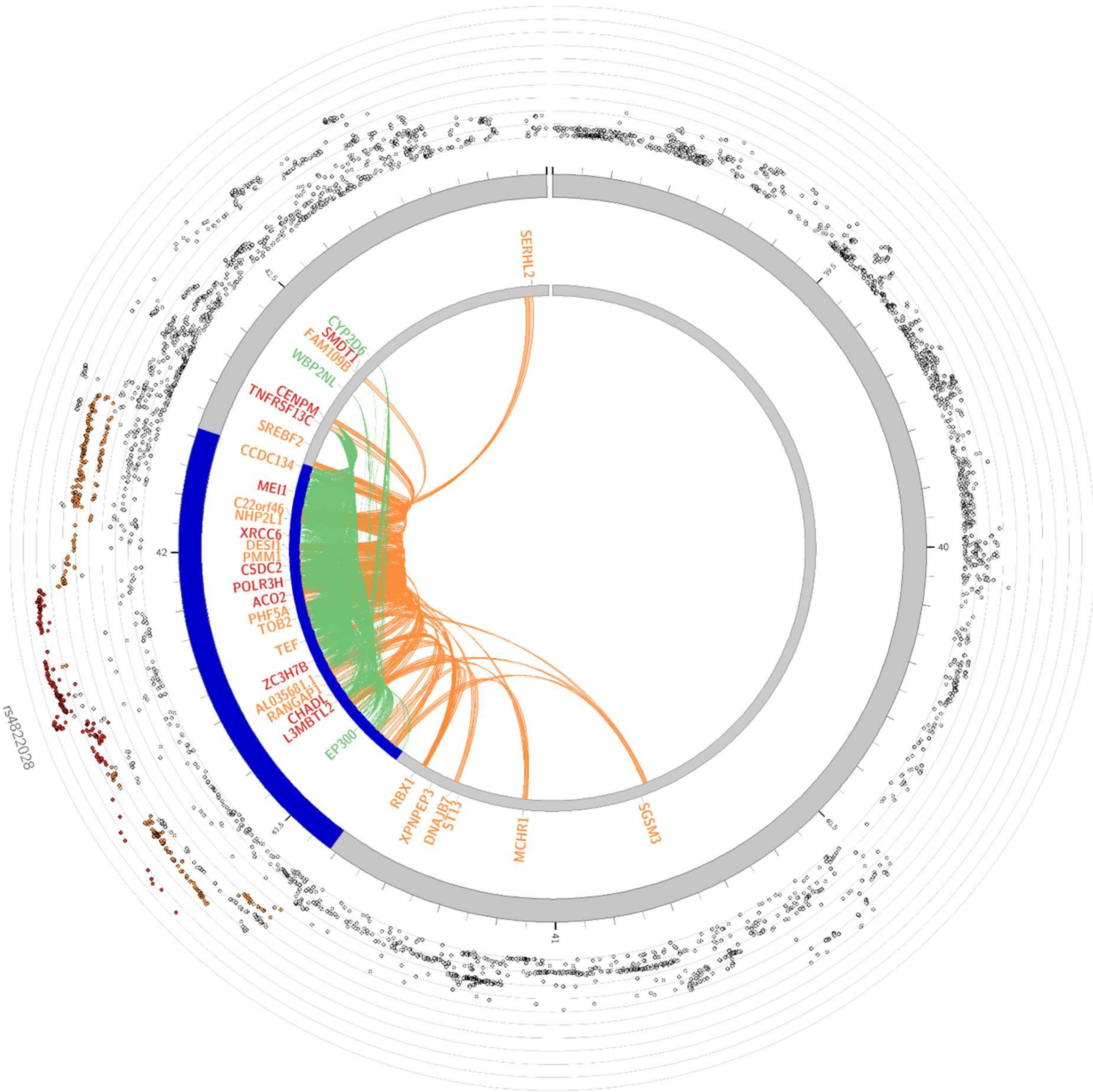

© 2022 Deak JD et al. *JAMA Network Open.*
