# Supplementary material for: Genome-Wide Investigation of Maximum Habitual Alcohol Intake in US Veterans in Relation to Alcohol Consumption Traits and Alcohol Use Disorder
Source: JAMA Netw Open. 2022 Oct 27;5(10):e2238880. doi: 10.1001/jamanetworkopen.2022.38880 (PMC9614582; doi:10.1001/jamanetworkopen.2022.38880)
Supplement: Supplement 3. — Nonauthor Collaborators [file jamanetwopen-e2238880-s003.pdf]

Supplemental Online Content: Nonauthor Collaborators
*First name, last name, and suffix (if applicable) are required and will appear in PubMed.

***Group Name(s): The Million Veteran Program**

***First Name and Middle Initial(s)**	***Last Name**	***Suffix (eg, Jr, III)**	Academic Degrees	Institution	Location (city, state/province, country)	Role or Contribution, eg, chair, principal investigator	Group (if more than 1 Group listed in the byline) and/or Subgroup (eg, Steering Committee)
Sumitra	Muralidhar		Ph.D.	US Department of Veterans Affairs	Washington, DC	Program Director	**MVP Program Office**
Jennifer	Moser		Ph.D.	US Department of Veterans Affairs	Washington, DC	Associate Director, Scientific Programs	**MVP Program Office**
Jennifer E.	Deen		B.S.	US Department of Veterans Affairs	Washington, DC	Associate Director, Cohort Management & Public Relations	**MVP Program Office**
J. Michael	Gaziano		M.D., M.P.H.	VA Boston Healthcare System	Boston, MA	Co-Chair	**MVP Executive Committee**
Sumitra	Muralidhar		Ph.D.	US Department of Veterans Affairs	Washington, DC	Co-Chair	**MVP Executive Committee**
Jean	Beckham		Ph.D.	Durham VA Medical Center	Durham, NC	Commitee member	**MVP Executive Committee**
Kyong-Mi	Chang		M.D.	Philadelphia VA Medical Center	Philadelphia, PA	Commitee member	**MVP Executive Committee**
Philip S	Tsao		Ph.D.	VA Palo Alto Health Care System	Palo Alto, CA	Commitee member	**MVP Executive Committee**
Shiuh-Wen	Luoh		M.D., Ph.D.	VA Portland Health Care System	Portland, OR	Commitee member	**MVP Executive Committee**
Juan P.	Casas		M.D., Ph.D	VA Boston Healthcare System	Boston, MA	Commitee member	**MVP Executive Committee**
Juan P.	Casas		M.D., Ph.D	VA Boston Healthcare System	Boston, MA	Executive Director	**MVP Operations**
Lori	Churby		B.S.	VA Palo Alto Health Care System	Palo Alto, CA	Director of Regulatory Affairs	**MVP Operations**
Stacey B.	Whitbourne		Ph.D.	VA Boston Healthcare System	Boston, MA	MVP Cohort Management Director	**MVP Operations**
Jessica V.	Brewer		M.P.H.	VA Boston Healthcare System	Boston, MA	MVP Recruitment/Enrollment Director	**MVP Operations**
Mary T.	Brophy		M.D., M.P.H.	VA Boston Healthcare System	Boston, MA	Director, VA Central Biorepository	**MVP Operations**
Luis E.	Selva		Ph.D.	VA Boston Healthcare System	Boston, MA	Executive Director for MVP Biorepositories	**MVP Operations**
Shahpoor	Shayan		M.S.	VA Boston Healthcare System	Boston, MA	MVP Informatics	**MVP Operations**
Kelly	Cho		Ph.D., M.P.H.,	VA Boston Healthcare System	Boston, MA	Director, MVP Data Operations/Analytics	**MVP Operations**
Saiju	Pyarajan		Ph.D.	VA Boston Healthcare System	Boston, MA	Director, Center for Computational and Data Science (C-DACS) & Genomics Core	**MVP Operations**
Philip S	Tsao		Ph.D.	VA Palo Alto Health Care System	Palo Alto, CA	Director, Molecular Data Core	**MVP Operations**
Kelly	Cho		Ph.D., M.P.H.,	VA Boston Healthcare System	Boston, MA	Director, Phenomics Data Core	**MVP Operations**
Scott L.	DuVall		Ph.D.	VA Salt Lake City Health Care System	Salt Lake City, UT	Director, VA Informatics and Computing Infrastructure (VINCI)	**MVP Operations**
Todd A.	Connor		Pharm.D.	New Mexico VA Health Care System	Albuquerque, NM	Cooperative Studies Program Clinical Research Pharmacy Coordinating Center	**MVP Operations**
Dean P.	Argyres		B.S., M.S.	New Mexico VA Health Care System	Albuquerque, NM	Cooperative Studies Program Clinical Research Pharmacy Coordinating Center	**MVP Operations**
Mihaela	Aslan		Ph.D.	VA Boston Healthcare System	Boston, MA	Clinical Epidemiology Research Center (CERC) Coordinator	**MVP Recruitment/Enrollment**
Philip S	Tsao		Ph.D.	VA Palo Alto Health Care System	Palo Alto, CA	Genomics Coordinating Center, Palo Alto	**MVP Recruitment/Enrollment**
J. Michael	Gaziano		M.D., M.P.H.	VA Boston Healthcare System	Boston, MA	MVP Boston Coordinating Center, Boston	**MVP Recruitment/Enrollment**
Brady	Stephens		M.S.	Canandaigua VA Medical Center	Canandaigua, NY	MVP Information Center, Canandaigua	**MVP Recruitment/Enrollment**
John	Concato		M.D., M.P.H.	VA Connecticut Healthcare System	West Haven, CT	Commitee member	**CSP#575B Executive Committee**
J. Michael	Gaziano		M.D., M.P.H.	VA Boston Healthcare System	Boston, MA	Commitee member	**CSP#575B Executive Committee**
Joel	Gelernter		M.D.	VA Connecticut Healthcare System	West Haven, CT	Commitee member	**CSP#575B Executive Committee**
Terri	Gleason		Ph.D.	US Department of Veterans Affairs	Washington, DC	Commitee member	**CSP#575B Executive Committee**
Grant D.	Huang		Ph.D., M.P.H.,	US Department of Veterans Affairs	Washington, DC	Commitee member	**CSP#575B Executive Committee**
Karestan C.	Koenen		Ph.D.	Harvard Chan School of Public Health	Boston, MA	Commitee member	**CSP#575B Executive Committee**
Christine	Marx		M.D.	Durham VA Medical Center	Durham, NC	Commitee member	**CSP#575B Executive Committee**
Jennifer	Moser		Ph.D.	US Department of Veterans Affairs	Washington, DC	Commitee member	**CSP#575B Executive Committee**
Krishnan	Radhakrishnan		M.D.	VA Connecticut Healthcare System	West Haven, CT	Commitee member	**CSP#575B Executive Committee**
Nicholas	Schork		Ph.D.	J Craig Venter Institute	La Jolla, CA	Commitee member	**CSP#575B Executive Committee**
Murray	Stein		M.D., M.P.H.	VA San Diego Healthcare System	San Diego, CA	Commitee member	**CSP#575B Executive Committee**
Hongyu	Zhao		Ph.D.	VA Connecticut Healthcare System	West Haven, CT	Commitee member	**CSP#575B Executive Committee**
Joel	Gelernter		M.D.	VA Connecticut Healthcare System	West Haven, CT	Study Co-Chair	**CSP#575B Study Chairs' Offices**
Murray	Stein		M.D., M.P.H.	VA San Diego Healthcare System	San Diego, CA	Study Co-Chair	**CSP#575B Study Chairs' Offices**
Joan	Kaufman		Ph.D.	VA Connecticut Healthcare System	West Haven, CT	Member	**CSP#575B Study Chairs' Offices**
Yaira	Nunez		B.S	VA Connecticut Healthcare System	West Haven, CT	Member	**CSP#575B Study Chairs' Offices**
Robert H.	Pietrzak		Ph.D.	VA Connecticut Healthcare System	West Haven, CT	Member	**CSP#575B Study Chairs' Offices**
Danielle	Beck		M.P.H.	VA San Diego Healthcare System	San Diego, CA	Member	**CSP#575B Study Chairs' Offices**
Shada	Cissell		LCSW	VA San Diego Healthcare System	San Diego, CA	Member	**CSP#575B Study Chairs' Offices**
Mihaela	Aslan		Ph.D.	VA Connecticut Healthcare System	West Haven, CT	VA Clinical Epidemiology Research Center (CERC)	**CSP Epidemiology Centers**
John	Concato		M.D., M.P.H.	VA Connecticut Healthcare System	West Haven, CT	VA Clinical Epidemiology Research Center (CERC)	**CSP Epidemiology Centers**
Patricia	Crutchfield		B.S	VA Connecticut Healthcare System	West Haven, CT	VA Clinical Epidemiology Research Center (CERC)	**CSP Epidemiology Centers**
William	Lance		M.B.A., M.P.A., M.S.	VA Connecticut Healthcare System	West Haven, CT	VA Clinical Epidemiology Research Center (CERC)	**CSP Epidemiology Centers**
Kei-Hoi	Cheung		Ph.D	VA Connecticut Healthcare System	West Haven, CT	VA Clinical Epidemiology Research Center (CERC)	**CSP Epidemiology Centers**
Yuli	Li		M.S.	VA Connecticut Healthcare System	West Haven, CT	VA Clinical Epidemiology Research Center (CERC)	**CSP Epidemiology Centers**
Ning	Sun		Ph.D.	VA Connecticut Healthcare System	West Haven, CT	VA Clinical Epidemiology Research Center (CERC)	**CSP Epidemiology Centers**

Supplemental Online Content: Nonauthor Collaborators
*First name, last name, and suffix (if applicable) are required and will appear in PubMed.

***First Name and Middle Initial(s)**	***Last Name**	***Suffix (eg, Jr, III)**	Academic Degrees	Institution	Location (city, state/province, country)	Role or Contribution, eg, chair, principal investigator	Group (if more than 1 Group listed in the byline) and/or Subgroup (eg, Steering Committee)
Quan	Chen		Ph.D.	VA Connecticut Healthcare System	West Haven, CT	VA Clinical Epidemiology Research Center (CERC)	**CSP Epidemiology Centers**
Krishnan	Radhakrishnan		M.D., Ph.D., M.P.H., C.P.H	VA Connecticut Healthcare System	West Haven, CT	VA Clinical Epidemiology Research Center (CERC)	**CSP Epidemiology Centers**
Nallakkandi	Rajeevan		Ph.D.	VA Connecticut Healthcare System	West Haven, CT	VA Clinical Epidemiology Research Center (CERC)	**CSP Epidemiology Centers**
Frederick	Sayward		Ph.D.	VA Connecticut Healthcare System	West Haven, CT	VA Clinical Epidemiology Research Center (CERC)	**CSP Epidemiology Centers**
J. Michael	Gaziano		M.D., M.P.H.	VA Boston Healthcare System	Boston, MA	Massachusetts Veterans Epidemiology Research and Information Center	**CSP Epidemiology Centers**
Kelly	Cho		Ph.D., M.P.H.,	VA Boston Healthcare System	Boston, MA	Massachusetts Veterans Epidemiology Research and Information Center	**CSP Epidemiology Centers**
David R.	Gagnon		M.D., Ph.D	VA Boston Healthcare System	Boston, MA	Massachusetts Veterans Epidemiology Research and Information Center	**CSP Epidemiology Centers**
Kelly	Harrington		Ph.D.	VA Boston Healthcare System	Boston, MA	Massachusetts Veterans Epidemiology Research and Information Center	**CSP Epidemiology Centers**
Rachel	Quaden		M.A.	VA Boston Healthcare System	Boston, MA	Massachusetts Veterans Epidemiology Research and Information Center	**CSP Epidemiology Centers**
Saiju	Pyarajan		Ph.D.	VA Boston Healthcare System	Boston, MA	Massachusetts Veterans Epidemiology Research and Information Center	**CSP Epidemiology Centers**
Stacey B.	Whitbourne		Ph.D.	VA Boston Healthcare System	Boston, MA	Massachusetts Veterans Epidemiology Research and Information Center	**CSP Epidemiology Centers**
Terri	Gleason		Ph.D.	US Department of Veterans Affairs	Washington, DC	Clinical Science Research and Development Service	**VA Office of Research and Development**
Grant D.	Huang		Ph.D., M.P.H.,	US Department of Veterans Affairs	Washington, DC	Cooperative Studies Program	**VA Office of Research and Development**
Jennifer	Moser		Ph.D.	US Department of Veterans Affairs	Washington, DC	Program Manager, Million Veteran Program	**VA Office of Research and Development**
Sumitra	Muralidhar		Ph.D.	US Department of Veterans Affairs	Washington, DC	Million Veteran Program, Director	**VA Office of Research and Development**
Timothy	O'Leary		M.D., Ph.D.	US Department of Veterans Affairs	Washington, DC	Chief Research and Development Officers	**VA Office of Research and Development**
Rachel B	Ramoni		D.M.D., Sc.D	US Department of Veterans Affairs	Washington, DC	Chief Research and Development Officers	**VA Office of Research and Development**
							**VA Office of Research and Development**
